# Extreme Gradient Boosting Combined with Conformal Predictors for Informative Solubility Estimation

**DOI:** 10.3390/molecules29010019

**Published:** 2023-12-19

**Authors:** Ozren Jovic, Rabah Mouras

**Affiliations:** Pharmaceutical Manufacturing Technology Centre, Bernal Institute, Department of Chemical Sciences, University of Limerick, V94 T9PX Limerick, Ireland; ozren.jovic@ul.ie

**Keywords:** solubility, machine learning, extreme gradient boosting, variable selection, conformal predictor, prediction interval, applicability domain, molecular descriptor

## Abstract

We used the extreme gradient boosting (XGB) algorithm to predict the experimental solubility of chemical compounds in water and organic solvents and to select significant molecular descriptors. The accuracy of prediction of our forward stepwise top-importance XGB (FSTI-XGB) on curated solubility data sets in terms of RMSE was found to be 0.59–0.76 Log(S) for two water data sets, while for organic solvent data sets it was 0.69–0.79 Log(S) for the Methanol data set, 0.65–0.79 for the Ethanol data set, and 0.62–0.70 Log(S) for the Acetone data set. That was the first step. In the second step, we used uncurated and curated AquaSolDB data sets for applicability domain (AD) tests of Drugbank, PubChem, and COCONUT databases and determined that more than 95% of studied ca. 500,000 compounds were within the AD. In the third step, we applied conformal prediction to obtain narrow prediction intervals and we successfully validated them using test sets’ true solubility values. With prediction intervals obtained in the last fourth step, we were able to estimate individual error margins and the accuracy class of the solubility prediction for molecules within the AD of three public databases. All that was possible without the knowledge of experimental database solubilities. We find these four steps novel because usually, solubility-related works only study the first step or the first two steps.

## 1. Introduction

Solvents’ selection in processes plays an important role in pharmaceutical manufacturing, especially in catalysis, synthesis, separation, formulation, drug discovery, pharmaceutical cleaning, etc. Therefore, the estimation of compounds’ solubility has become one of the major challenges in computational chemistry and machine learning (ML) since the last century, considering a wide range of theoretical and statistical approaches [1,2,3]. Theoretical approaches include Hildebrand and Hansen solubility parameters [4,5], COSMO-RS and COSMOtherm [6,7], the Flory–Huggins model [8,9], UNIFAC [10], and others. [11]. For large data sets, ML methodologies link the compound’s experimental solubilities at acceptably narrow temperature ranges as the dependent variable, with the large descriptor, fingerprint, or theoretical chemistry-derived properties (like solvation of Gibbs free energy or chemical potentials) as independent variables and build a regression model for solubility prediction [11,12,13]. The majority of published publications mostly focus on water solubility prediction. Very recently, researchers have started predicting solubility in other organic solvents [11,12,14].

What has been shown up to now is that nonlinear methodologies such as gradient boosting (GB) [11,15] and random forest (RF) [11] significantly outperform linear methodologies such as partial least-squares regression (PLS) in solubility predictions [11,12]. However, there are still two major issues not addressed at all or insufficiently explained. The first issue is the expert variable and algorithm selection and the second is a reliable extrapolation of solubility prediction models to large databases of unknown experimental solubilities using both applicability domain (AD) [16,17,18,19,20] and conformal prediction [21,22,23,24]. While the former is to some extent elaborated [11,12], to the best of our knowledge, the latter has not been applied in the known literature on in silico solubility prediction. For drug-like properties such as the water–octanol distribution coefficient (Log*D*), there has already been a large-scale confidence predictor introduced with support vector machines which predicts all PubChem compounds [23] but without the use of a necessary applicability domain test [20] for supporting that extrapolated predictions to the public domain.

Regarding solubility prediction using quantitative structure–property relationship (QSPR) [25], in the Drugbank database [26], water solubilities have already been calculated using the AlogPS method [27]. But when referencing the source of such calculation in the Drugbank, one is only forwarded to Ref. [27] which references the solubility study from 2001 [28]. Now, we see the following issues:

(1) On the reference web page [27], among many stated articles, one is related to the applicability domain [29] from 2008. The independent literature search results found only a web link [30] last updated in 2014, where they cite Ref. [29] stating that the prediction accuracy of an unknown set is estimated using “ASNN-STD” under the AD section. However, that link [30] was not cited by Drugbank. Instead, under Drugbank publications, the last update related to solubility prediction was in 2007 [31] and they only referenced the AlogPS2.1 program from 2002 [32]. In Ref. [32], only similarity measures between compounds were considered.
(2) Ref. [28] displays in one of its figures the dependency of the prediction accuracy on the compound’s number of nonhydrogen atoms. This means that based on their own used reference for solubility extrapolation to the Drugbank database, molecules with different molecular properties (e.g., molecular mass) must have a different difficulty of being predicted. When prediction models are extrapolated to large databases, this must be accounted for each compound. Thus, besides the predicted solubility, there should also be a difficulty class related to that prediction. But there are no signs of any such solubility classification in Drugbank or any other database [33,34].
(3) There is no approximative statistic (e.g., median prediction interval at a certain confidence level) given for the successfulness of the whole solubility extrapolation to large databases.

Obviously, the literature related to solubility prediction is too focused on proving that the prediction accuracy of a novel method should outperform prior methods on small data sets [11,35]. There is no clear and informative up-to-date attempt for extrapolation of an accurate and/or robust methodology to large databases, which would informatively serve the scientific community.

Based on solubility experimental data, predictions of organic solubilities can be off by at least 0.5 Log(*S*/mol L^−1^) (in later text Log(S)), while the typical range is 0.5–0.7 [12]. As computational accuracy using QSPR depends on the compound’s molecular properties [12,28], equal-width prediction intervals for all samples produced with conventional regression methods [21,23] would imply that the calculated solubility of each particular substance carries the same error margin within the test set. This generally cannot be expected to be true, especially if the training set is not composed of balanced fractions of compounds of similar properties when compared to the test set, as is the case with model prediction of large databases from the much smaller training model. In that context, a conformal predictor (CP) can improve data informativeness by obtaining a half-width margin for each sample. The idea that stands behind employing conformal prediction is to increase the informativeness of each prediction by narrowing its prediction interval. This has to be supported by AD rules from the start, by carefully choosing the appropriate train–test split and determining that the test set is within the AD of the training set [36,37] before ML [3,38,39,40] or deep learning [2,41,42] is conducted. The initial train–test split can be obtained with a good initial guess for molecular descriptors as original variables used for building AD rules [37] so that later selected optimal molecular descriptors from the ML algorithm reconfirm priorly the AD-established train–test split and use it for extrapolation to large databases. A superficial selection of the train–test split or a random selection [43] could result in the later weaker training set AD coverage of the targeted database compounds.

For the prediction of solubilities in this study, we introduce and apply a methodology that has not been used before in solubility studies, called extreme gradient boosting (XGB), one of the best-performing classification and regression methodologies [15,44,45]. The advantage of XGB when compared to artificial neural networks [28] is that XGB does not need to be supported by a multilinear methodology when selecting optimal descriptors. XGB uses its importance metric to select informative variables and filter off unimportant ones similarly to GB and RF [11], but XGB uses residuals of the previous iteration for the initial guess of the new iteration, while at the same time, XGB also minimizes the overfitting problems that might appear in GB [45]. Deep learning methodologies [46], including transfer learning (TL) [43], have also become popular in the recent years for estimating drug-like properties [47].

Therefore, the aim of this study is very clear; it is to predict water solubilities in external databases of a total of ca 500,000 compounds as accurately and informatively as possible using the AqSolDB set [48] of only 9982 compounds. Among the whole AqSolDB set, only a smaller curated fraction has an experimental standard deviation of at most 0.5 Log(S). In doing so, we use XGB coupled with variable selection, applicability domain rules, and conformal predictors. We prove that using conformal prediction, the database compounds can be classified into different accuracy groups using an estimate of accuracy in the terms of percentage of predictions within ±1.0 Log(S) (%LogS ± 1.0) from experimental values. This measure represents the limits of applicability of the model as a guiding tool for process/product development [12]. Such large database classification certainly has not been conducted before, to the best of our knowledge. In this study, we provide consistent answers to all three points (1–3) raised above. Namely, in conjunction with proving the superiority of XGB over non-deep learning RF recommended in one of the very recent solubility articles [49] and a comparison of XGB with the TL methodology, we also: (1) define the AD and train–test split with initial AqSolDB descriptors and display the final AD extrapolation results to the public databases using later-selected optimal descriptors on the training set; (2) assess all our independent test data sets of water and organic solvents’ solubilities with error margin statistics at certain confidence levels and classify predicted solubilities to different accuracy classes on large compound databases; (3) display and explain the general statistics of our extrapolation results to large databases.

## 2. Results and Discussion

### 2.1. Model Accuracy and Variable Selection on Data Sets (1–6)

Table 1 and Table 2 present a comparison between RF and XGB methodologies on two large AquaSolDB data sets using the same variables for both methods. No variable selection in this case was performed, in order to directly compare RF with XGB. From both tables can be clearly seen that XGB outperforms RF (and its fine-tuned version) in all accuracy indices of both data sets. As has already been stated, the AqSolDB-n model built on only the training set using a 20-fold CV was tested by two independent test sets, one internal of 220 compounds and the other external Water-wide data set. In all 15 indices, XGB was stronger, and the differences were not negligible. A two-tailed paired *t*-test on the squared residuals of the RMSEP (T) set showed a statistically significant difference between RF and XGB (*p* < 0.01). Similar results held between fine-tuned RF and XGB methods (*p* = 0.011, i.e., *p* < 0.05) (Table 1). Regarding the second AquaSolDB-w set (Table 2), in all eight indices, XGB also attained higher performance, with a significant *t*-test between RMSEV sets (*p* < 0.05), while the RMSECV difference was even more radical as the F-test on the residuals showed a high statistical significance (*p* < 0.001). Supplementary Info Tables S1a and S1b in Supplementary_documentation.pdf display the results of XGB and fine-tuned RF on four other smaller data sets (1, 4–6) (see also comments below Tables S1a and S1b). As the task of this work was to use the large data set AqSolDB-n for building the prediction model and extrapolating it to large databases, XGB had to be selected as a method of choice. XGB in this form was a complex model of at least 532 variables and had to undergo a variable selection before model extrapolation to external databases. Supplementary Figure S1 displays the predictions obtained on the XGB modelling of the AqSol-DB-w set; its median absolute residuals for the test validation was 0.4110 Log(S). The average absolute residuals for the CV and test validation were 0.6349 and 0.6221 Log(S), respectively.

Table 3 displays the performance of all our FSTI-XGB models in organic solvents on different variable sets, with the aim of determining how the use of different variables impacts the predictive performance of the obtained optimized models. From the table, it can be observed that models with both Padel and QM variables (Section 3.1) produced the strongest models. For all three organic solvent sets with the FSTI-XGB model, the difference between QMvars and Pvars + QMvars regarding cross-validated training residuals was significant at *p* < 0.05 (based on an equal variance two-tailed *t*-test on squared residuals). Thus, models with more diverse variables generally led to stronger performance. It is therefore difficult to expect that a model containing ca. 20–35 variables should be higher or very similar in accuracy compared to 532 (or 1146) variable models, but only that it does not lose too much predictive strength.

**Figure 1:**
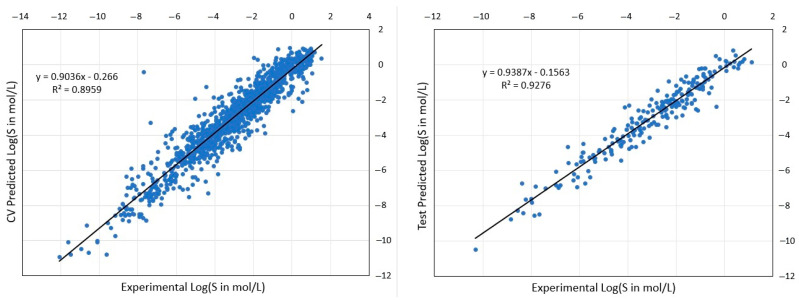
Prediction performance of the FSTI-XGB model on the AqSolDB-n set (3), with 26 molecular descriptors. The left plot shows statistics of 1399 compounds from the CV training set, and the right plot shows those of 220 compounds from the test set.

Figure 1 displays our FSTI-XGB results on the AqSolDB-n data set (3), while Figures S2 and S3 show the FSTI-XGB results on other data sets (1–2, 4–6). The predicted vs. experimental Log(S) on internal test sets for every compound is presented in Supplementary_data_sets_table.xlsx for all six test validation sets (1–6). Table 4 presents our FSTI-XGB results on both AqSolDB data sets (2–3) and their comparison with Refs. [3,25] results, while Table 5 presents the model performance of our four smaller data sets (1, 4–6) and their analysis with Ref. [12]. Below the tables are reported the fine-tuned parameters: max_depth (md), eta (eta), and nrounds (nr), so that given the exact train–test split labelled in Supplementary_data_sets_table.xlsx and given the selected descriptors named below and in Supporting Info Section S1 of Supplementary_documentation.pdf (with obtained importance values), our models can be exactly reproduced using R software version 4.1.2. More details with a simple code file “Test_repr_code.R” can be found on GitHub. Our three larger data sets, AqSolDB-w, AqSolDB-n, and Water-wide data set [12] selected larger max_depth parameters (range of 4–6) than our other three smaller data sets (of max_depth range 2–3), as was similarly reported for the XGB methodology [44] that for smaller sets, a smaller max_depth is expected to avoid overfitting.

FSTI-XGB selected the following 32 descriptors (sorted top-down in importance) on AqSolDB-w: MolLogP, XLogP, TpiPC, BalabanJ, BertzCT, AATS1i, MolWt, GATS1s, AATSC2e, piPC1, GATS2c, TPSA, piPC3, ZMIC1, Mv, MATS1e, MolMR, AATS7p, AATS3v, AATS6v, GATS1m, MWC3, TPC, MDEO.11, MDEC.33, nAtomP, AATS1v, AATS4v, AATS4m, AATS7v, AATS0v, nAcid.

For AqSolDB-n, FSTI-XGB selected the following 26 descriptors (sorted top-down in importance) from AqSolDB-n: MolLogP, ATS0p, XLogP, ZMIC1, GATS2c, piPC2, MPC7, AATS1i, MDEC-33, piPC3, AATS6v, TpiPC, nH, AATS5p, AATS1e, ATS1m, ZMIC2, TWC, piPC6, MPC8, AATS4v, MolMR, piPC1, piPC10, Mi, piPC4.

FTSI-XGB for the Methanol set (1) selected ΔHfus..kJ.mol.1 and deltaG.aver as QMvars in conjunction with 32 Pvars. Thus, our calculations with ORCA DFT on the Solvation Gibbs free energy did contribute to the solubility estimations in organic solvents. For the Water set (3), the selected QMvars were MP and HOMO, while for the Acetone set (5), they were MP, Asp1, and Area3. The full list of selected FSTI-XGB descriptors sorted by top-down importance for the Methanol set (1), Water set (3), Ethanol set (4), and Acetone set (5) can be found in Supporting Info Section S1.

As FSTI-XGB used at most up to 34 descriptors, contrary to XGB and RF with hundreds of descriptors (Table 1 and Table 2), it is interesting to note that the CV-set statistics of FSTI-XGB even somewhat outperformed XGB on smaller data sets (1, 4–6), while the *t*-test showed no significant overfitting. For AquaSolDB-w, which was later used to predict a smaller fraction of database compounds, the paired *t*-test on squared RMSEV residuals showed a significant difference between XGB and FSTI-XGB (*p* < 0.01), but the F-test on residuals did not show that (*p* > 0.05). Therefore, there was some difference in performance as could be expected from the analysis and discussion of Table 3, but it was still acceptable after the loss of many molecular descriptors. This was not the same as a direct comparison between RF and XGB using the same variable set. For AqSolDB-n, which was later used to predict most database compounds, there was no statistically significant difference between XGB and FSTI-XGB, concerning both paired *t*-test and F-test RMSEV and RMSEP residuals (*p* > 0.05) (e.g., XGB RMSEP = 0.755 Log(S) vs. FSTI-XGB RMSEP = 0.764 Log(S)). This means our FSTI-XGB managed to carry out solid variable selections with a low or insignificant loss in accuracy. This is important when applied to large databases. One cannot use the 532- or 1146-variable XGB model because the calculation of some of these descriptors for too many compounds in external databases would result in missing values, making the models not applicable for use.

Seven compounds outside the AD for AqSolDB-w, if excluded from the test set, would only slightly but insignificantly impact accuracy statistics in Table 4. If excluded, the test statistics would be the following: RMSEV = 0.962 Log(S), %LogS ± 0.7 = 67.6%, %LogS ± 1.0 = 80.0%.

We now compare our results with Refs. [3,25] in Table 4. FSTI-XGB on the larger AquaSolDB-w data set clearly outperformed the SolTranNet methodology described in Ref. [3] on both the CV-set and V-set. Other studies did not consider the use of the whole AquaSolDB for ML prediction to the best of our knowledge. Related to Ref. [25], we explain in Supporting Info Section S2 that test and validation subsets utilized in Ref. [25] were part of their optimization and final model selection, so their subsets were not independent of building and selecting the final solubility prediction model. That is different from our approach, as both internal test and external test sets we used were independent of the optimization and final model selection. Nevertheless, even if that and other details discussed in Info Section S2 were disregarded, our RMSEtot would still slightly outperform theirs. We also achieved a stronger *R*^2^(V) (Table 4). Other studies did not use the curated AquaSolDB for ML Log(S) prediction to the best of our knowledge.

Our FSTI-XGB results of smaller data sets (1, 4–6) can be directly compared with two top models in Ref. [12] that produced the lowest RMSECV for each data set (Table 5). These are SVM and GP for the Water, RF and Bag for the Ethanol, and SVM and Bag for the Acetone data sets (see their RMSECV in the supplementary information of Ref. [12]). It can be seen that our FSTI-XGB outperformed these mentioned methodologies in most statistics displayed in Table 3. The average of the %LogS ± 0.7 and %LogS ± 1.0 validation statistics was also advantageous with our FSTI-XGB methodology. The RMSE performance on the Acetone set (RMSECV = 0.62 and RMSEV = 0.7) was even comparable with the top predicted RMSE values using non-TL methods ranging from 0.56 to 0.75 Log(S) [11,12,25,49]. The predicted vs. experimental Log(S) is presented in Figure S3 for the test set of our data sets (1, 4–6).

We have observed in ML references that solubility predictions are often conducted without an independent test set, i.e., without a set completely independent from the fine-tuning of the calibration set or from the final model selection based on validation indices [11,25,49]. For example, the whole set is split into the training and validation sets and after fine-tuning the validation set, a five-fold CV on the whole set is considered as the final test performance [11]. That can hardly be compared to our approach, since in our test set (V-set), not even a single test validation sample had any role in the training, optimization, or final model selection in the accuracy optimization. The reason is that we needed an independent test set for the later inductive conformal prediction [22]. Ref. [12] had an independent training set, to which a 10-fold CV was applied, and an independent test set, so we could compare our results with the reference. We also needed an external test set (i.e., Water-wide) for the AqSolDB-n model for an additional validation of CP error rates.

Regarding ALOGPS, Ref. [28], using artificial neural networks, obtained an RMSE on the test set as low as 0.67 Log(S) [28,35] on only 21 independent test compounds, while other training and optimization sets had altogether 1291 compounds. The test set’s RMSE coincided with their optimized validation set of 412 molecules. However, any model performance on only 21 cases can be obtained by simple chance. For example, we could state that ridge regression as a linear method had the second top performance (e.g., better than RF or ET [12]) on 20 unseen solutes and was close to the top-performing lightGBM in Table 4 of Ref. [11], but the conclusion of the article is that the nonlinear methodologies outperformed ridge regression. Usually, if any method outperforms another methodology, a test for statistical significance on the residuals should confirm that. However, we have not noticed such statistical tests (e.g., *t*-test or F-test) on residuals in many ML solubility articles. For instance, Ref. [12], when referencing its supplementary information uses the term “significant” on several occasions in its main text. But in the whole supplementary information, that term is only used once in Table S30′s caption, where they talk about a significant improvement of the nonlinear models when amines and carboxylic acids are excluded from the test samples. The content of Table S30 regards a test set of 75 compounds with amines, carboxylic acids, and the rest of the compounds with the four-method RMSE span of 1.09–1.23 Log(S). When all compounds containing amines and acids were removed from the test set, the RMSE of all models dropped to 0.6–0.79 Log(S) but on only 14 test compounds, as only 14 compounds remained in that test set. There is no mention of a statistical test between residuals of the 75-compound set and 14-compound set examples, neither in the table nor in the whole supplementary information.

Still, Ref. [28] explained in depth significant indices, and it was right about the advantage of nonlinear methodologies against linear methodologies. Linear methodologies usually underperform in water solubility predictions with a high RMSE >1.15 [12,25], while the average CV statistics of six nonlinear methods on an 805-sized compound set of the Water-wide set of Ref. [12] was RMSECV = 0.897 Log(S) with a span of 0.85–0.95 Log(S). For the 95-sized test set, the span was 071–0.85 [12], with an average of 0.823 Log(S) [12]. That is why we did not consider linear methods. An additional reason is that XGB can do its own variable selection using its importance metric on evaluated variables, by selecting only the top important variables, so that PLS regression coefficients are unnecessary. On the contrary, Ref. [28] needed a multilinear regression for the variable selection to obtain an improved performance with an ANN, and this is an advantage of an XGB methodology such as FSTI-XGB. Still, in QSPR, linear methodologies can have a solid performance, such as the RMSE 0.84–0.873 Log(S) obtained in Ref. [50] for 3664 compounds, similarly to Ref. [36], which involved an intrinsic solubility prediction with QSPR on fewer sample data sets.

The transfer learning (TL) models were able to achieve an average Log(S) RMSE of only 0.47–0.60 Log(S) on the curated data sets of four solvents and 2511 solutes [47], although the exact train–validation–test sample split for the solubility data sets was not detailed in Ref. [47]. The TL methodology used QM-calculated solvation free energies from the external database CombiSolv-QM to firstly pretrain their curated data sets in an unsupervised manner and later to fine-tune them in a supervised manner while making solubility predictions and producing fingerprints. The main difference in their approach compared to ours is that in addition to the fact that no AD rules were considered in Ref. [47], no descriptor input parameters are disposable for an unsupervised ML masked language model. This means that initial guess features for the AD train–test split are practically not available. Thus, one has to start conducting the TL approach on random splits [43] and later obtain fingerprints in the pretraining process to make an initial AD guess for the appropriate train–test split. This complicates the introduction of the AD. Also, it is unknown how fine-tuned fingerprints obtained later in the fine-tuning step alter AD results relative to pretraining fingerprints, but MinHash fingerprints appear to be too different from SolvBERT-built [47] fingerprints to be considered as an initial guess, at least regarding Figure 7 of Ref. [47]. Our FSTI-XGB-selected descriptors successfully verified the initially selected train–test split of 17 AqSolDB descriptors. Besides obvious AD issues for the TL method at the start, more importantly, cross-validation (CV) was not carried out for SolvBERT’s fine-tuning TL methods. CV or bootstrap is essential for conformal prediction, but CV was only performed in Ref. [43]’s TL, which worked solely with solvation free energy predictions, not with a Log(S) prediction through the CombiSolv-QM data set. The result of the CV approach in Ref. [43] (Figure 6 of the same reference) shows a negligible difference between the pretrained models and experimental models of a directed message-passing neural network (D-MPNN), which is neither in accordance with the results of Table 3 in the same article nor to most conclusions raised in the paper. Also, the D-MPNN in [43] used a feature-based approach where features learned in the pretraining phase were fixed in TL and were not fine-tuned as in Ref. [47]. Therefore, it would be useful to test the CV performance for the Log(S) prediction using fine-tuned TL, as one would see if it could increase its CV prediction performance for Log(S) relative to non-TL models. However, there are no records of any transfer learning CV performance on Log(S) nor any record of fine-tuning the TL CV performance on other drug-like properties [43,47,51]. In Table 1, Table 2, Table 3, Table 4 and Table 5, we constantly use CV-related statistical metrics for later CP. The Log(S) performance in TL was conducted on train–test–validation splits of SolvBERT and GROVER models [51], but to fully pretrain and fine-tune the model, SolvBERT took 12 h and GROVER took 2.5 days [47], without utilizing a more expensive 10-fold CV approach. Indeed, conformal prediction is even more demanding, it requires a 10-fold CV repeated 10 times, which actually means it is an almost 100 times more computationally expensive procedure for the evaluation of predicted error margins. All that solely refers to the application to small data sets (of ca. 2000 compounds), not for the use of such models to extrapolate them to large external databases of ca. 100,000 compounds void of experimental Log(S). The practical applicability of the TL methodology is therefore very limited, if at all possible, regarding the potential utilization of the AD and CPs for an external database analysis. Our FSTI-XGB can use its already optimized hyperparameters for each cycle of 20 20-fold CV when calculating prediction intervals using CP. The full duration of the variable selection and expensive grid fine-tuning of AqSolDB-n and ARSS prediction interval is only two hours on a four-core i7 processor. It is very feasible to obtain ARSS prediction intervals within 20 min for the COCONUT database, although with prior ca. 12 h for calculating all necessary molecular descriptors using Padel software, version 2.21. Finally, concerning both Refs. [43,47], the significant model accuracy gain for TL methods is proved only on the smaller experimental data sets with more solvents. The Solvation-Exp data set has (at most) a total of 1368 solutes and 10,145 solute–solvent pair combinations [43] (actually, it has 8780 pairs [47]), while the significant difference in performance for TL was obtained for data sets with less than 20% of Solvation-Exp data [47]. Therefore, it is recommended to use TL only for small data sets with more different property predictions. In that context, it is not expected that we could significantly enhance our accuracy performance by using transfer-knowledge databases for one-solvent data sets, AqSolDB-w having 9709 solutes and AqSolDB-n having 1619 solutes. Only a significant gain on smaller data sets (1, 4–6) could be anticipated, but these data sets were not used for the Log(S) estimation of databases due to their small set size to cover AD of databases. For all the mentioned reasons, we could not consider the TL application in our approach.

We obtained an accuracy on the curated AqSolDB-n set and external Water-wide set stronger than all cited non-TL performance in the paragraph before the previous paragraph. On uncurated AqSolDB-w, there were two objective sources of errors. One was related to ca. 8200 compounds (i.e., the difference in the size between AqSolDB-w and AqSolDB-n) with either a high experimental solubility standard deviation or only one experimental measurement of Log(S). The other source of error was the approximation that SMILES of compounds composed of two or more molecular fragments can be simplified to SMILES of one contiguous largest fragment when creating molecular descriptors, which in some cases might lead to a high prediction error. The AquaSolDB-n set had neither of these two sources of errors, and that was the difference between an RMSEtot of 0.71 and 0.97 Log(S). We needed the less accurate AqSolDB-w to capture more compounds within the AD for Log(S) prediction. Thus, the removal of more fragment compounds and/or removal of any other less accurate solubility group would narrow our AD, making it less applicable to public domains. An expert compromise in obtaining a higher accuracy while narrowing the AD was not the focus of this study. Therefore, we found our FSTI-XGB models successful in attaining good accuracy performance, so our models can be used for the solubility prediction of large external databases.

### 2.2. Applicability Domain Results on External Databases

**Figure 2:**
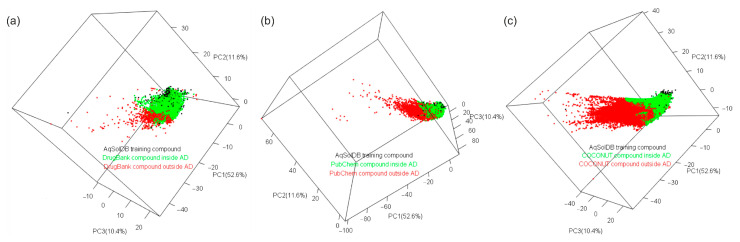
AD results displaying the PCA with the first three PCs, which cover 74.6% of the total variance of the AqSolDB-n training set for the (**a**) Drugbank database, (**b**) PubChem database, and (**c**) COCONUT database.

The AD results showed that 2.17% of Drugbank, 1.33% of PubChem, and 4.13% of COCONUT compounds were outside of the AqSolDB-w training set; the rest were inside the AD. Regarding AqSolDB-n, 413 Drugbank compounds were excluded at the start as they were two or more fragment compounds, so they could be only assessed with the AqSolDB-w model. Concerning the remaining 10,957 compounds, 10,413 were in the AD of the AqSolDB-n training set, i.e., 4.97% were outside the AD. Related to PubChem and COCONUT, outside the AqSolDB-n AD were 5.78% and 12.45% of the compounds, respectively. Figure 2 displays the AD results when the principal component analysis was carried out on 26 selected molecular descriptors of the AqSolDB-n training set and when the same descriptors of all compounds of three databases were projected on the axes of the first three principal components (PCs). Any database compound that was farther than the threshold of its closest AqSolDB-n training set compound was considered to be outside the AD. Therefore, all database compounds that were within the AD of AqSolDB-n were calculated only with its more accurate FSTI-XGB model, while all those compounds that were not within the AD of AqSolDB-n but that were inside AD of AqSolDB-w predicted Log(S) with the FSTI-XGB model of AqSolDB-w, as the difference in AD-covered compounds between our two models was significant.

### 2.3. Conformal Predictor (CP) Results on Data Sets (1–6)

The final results of all performed CP computations on all data sets can be found in detail in Tables S2a–S2p. In Table 6 (which is only a part of Table S2a), we display the important CP results on the FSTI-XGB model of AqSolDB-w. If the AR is compared to mean values of any normalized CP functions, then all normalized CPs obtained significantly (*p* < 0.01) narrower intervals at the 99% and 95% confidence levels, with the only exception of kNN-EuD for *β* = 0. The combination of the CP and sensitivity coefficient, *β,* is important to obtain the narrowest intervals for each specific confidence level. Hence, the tuning of *β is* necessary to achieve an “optimal” result for each specific CP function [22]. In this case, using the average of all eight statistics, the CP EM-N with *β = 0.3* achieved that (Table 4). Thus, italicized is the final result for a general combination of CP methodology and *β* for AqSolDB-w, while bolded are optimal solutions for each confidence level related to the minimum mean values among all combinations of CP functions and *β* values. We also display median values as they are very common in the literature [22,23,24]. For example, ARS *β* = 0.2 at the 80% confidence level has a mean of 0.959 log(S), and no other CP(*β*) has a lower mean value at the 80% confidence level. Therefore, EM-N at *β* = 0.3 is the general method of choice, but specifically at the 80% confidence level, ARS *β* = 0.2 is to be selected [23].

**Figure 3:**
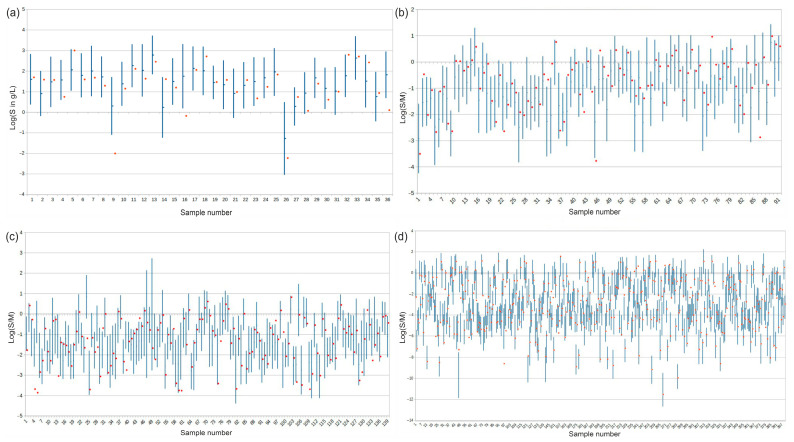
Solubility prediction intervals for test samples at the 90% confidence level for the (**a**) Methanol data set, (**b**) Acetone data set, (**c**) Ethanol data set, and (**d**) Water-wide data set. Blue line—prediction interval, red point—experimental Log(S). Details in Supp. Data Sets Tables.

However, not necessarily all combinations of CP functions, *β*’s, and confidence levels passed the validation on all data sets with experimental Log(S) values. There are several examples of 99% and 95% confidence levels of EM-N and EM-Log CPs on the AqSolDB-n external validation set in Table S2h where the error rate was too high (*t*-test, *p* < 0.01). In other cases, experimental Log(S) values fell within the predicted intervals with the statistically expected frequency. This means that almost all calculated intervals (Table S2) were successfully validated (*t*-test). An example of that is shown in Figure 3a displaying the methanol prediction intervals (blue line) at the 90% confidence level of methanol in pair with their experimental Log(S) (red dots).

Table 7 displays the final results for all data sets with the CP optimal solutions for each confidence level related to minimum mean values which passed the validation. Details related to which “optimal” *β* was selected in combination with CP can be found in Tables S2a–S2p. As Table 7 points out, normalized CP functions improved the efficiency by narrowing the median interval half-widths by more than 5% relative to the AR in 25 out of 28 total cases. A *t*-test at a *p* < 0.01 significance would show a statistically significant decrease in interval half-widths in 19 out of 25 cases, and in 4 cases at *p* < 0.05. The efficiency gain (calculated as [Q2(AR)/Q2(CP)]-1) was the highest for the AqSolDB-w data set (48–17%) when compared to other data sets. The obtained efficiency gain on AqSolDB-w and AqSolDB-n can be roughly compared with a drug-like similar property, Log*D* in Ref. [23] (Table 7), at least concerning the similarity in 99% and 95% confidence level gains. At the 90% and 80% confidence levels, the gain in this work was higher. The study which built the prediction interval for Log*D* only considered one *β* value for EM-N, of zero, and two *β*‘s of zero and one for the EM-Log methodology [23]. As we were analysing similar drug-like properties to solubility, which also used molecular descriptors as independent variables, we used more CP functions and *β* values, so we could significantly improve the efficiency by obtaining narrower intervals also at the 90% and 80% confidence levels.

Once the prediction intervals are produced for each compound, they can be used to approximatively assess %LogS ± 1.0 statistics for certain databases without the knowledge of experimental solubilities. For example, since the median prediction intervals at the 90% confidence level for AqSolDB-n internal test set equal 0.992 Log(S), this means that half of the compounds with narrower intervals (than 1 Log(S)) have at least 90% accuracy rate of being predicted within 1 Log(S) unit from the predicted Log(S) value. The other half with the wider intervals have less than a 90% accuracy rate. The mean is 1.029, so the overall accuracy can be roughly estimated to be at or slightly below 90%. A similar result is true for the Methanol data set with mean intervals of 1.038 Log(S) at the 90% confidence level. If Table 4 and Table 5 are reviewed one more time, one would see that for the V-set of AqSolDB-n %LogS ± 1.0 is 92.7%, which is slightly higher than 90% due to only a 5.45% error rate instead of the expected 10% at 90% confidence level. In Table 5, one can see that the Methanol data set’s %LogS ± 1.0 equals 86.1%. Thus, a similar thing can be stated for the yield lower than 90% for the Methanol set, as the error rate there (of 13.9%) exceeds 10%, but it is still statistically insignificant (Table S2n) due to a very small number of test samples for the CP validation (36). In the long run, when assessing large databases, for valid CPs, error rates do not exceed the chosen confidence threshold [24]. As for the Methanol data set, the Acetone and Ethanol sets also have considerable error rates (Table S2) that are not statistically significant. Ref. [23] did not consider any error rate analysis, but in this analysis, the importance of calculated error rates can be seen. For AqSolDB-w, there is a higher discrepancy between the mean and the median at 80% confidence (Table 7), and in that case, the 80% accuracy (when seven outliers out of the AD are excluded) coincides with the 80% accuracy roughly estimated from the mean (0.959 Log(S) at the 80% confidence level). This CP approach is even more useful for the classification of two different parts of the data set by compounds below and above the Log(S) half-width margin of one. The merit is high because such classification can be conducted on databases that are void of compounds’ experimental solubilities.

That is why we produced so many tables in Supporting Info Section S3, to select the most efficient CP at each confidence level and to prove that the CPs were valid (i.e., that the error rate was within the expected statistical frequency at the given confidence level in most cases). With that proof on both internal and external data sets, we used the “optimal” combination of CP and sensitivity factor (*β*) to produce informative prediction intervals on database molecules which were able to estimate the accuracy of our extrapolated predictions both individually (i.e., for each compound) and generally on entirely external databases. When the FSTI-XGB-ARSS model was extrapolated to the Drugbank database, each compound obtained its ordered pair of predicted Log(S) and its prediction interval at a certain confidence level, so we could classify the compounds into those whose prediction intervals were below ±1 Log(S) margin and to those above it. It is anticipated that different compounds should have different difficulties in being predicted, e.g., heavier compounds have a higher RMSE [28] and their Log(S) is more difficult to predict accurately, so they are expected to obtain wider intervals.

We established a positive significant *R*^2^ between absolute residuals and normalized NC interval half-widths of 0.174 (*p* < 0.001) on the AqSolDB-w set and of 0.241 (*p* < 0.001) on the AqSolDB-n set. A similar result was also observed for the Acetone (*p* < 0.001), Ethanol (*p* < 0.05), Methanol (*p* < 0.01), and Water-wide (*p* < 0.001) data sets. Individual molecular descriptors of both AqSolDB-s sets were less correlated with the absolute residuals than the obtained prediction intervals. On the AqSolDB-w set, the highest *R* had BalabanJ on the CV-set, *R* = −0.212 and MolWt on the V-set, *R* = +0.174, while the *R* value between prediction intervals and absolute residuals was 0.4219. This is still higher than the literature average correlations presented in Ref. [24] (see Table 8 of Ref. [24]), where for the best methodology, the average correlation coefficient value was 0.377. Therefore, the produced wider prediction intervals correctly statistically forecast a higher prediction error. This is how the informativeness of normalized NCs is advantageous over the AR. In the absence of experimental solubilities, the half-width can be individually produced to assess every test sample. Although wider than average prediction intervals (than AR) are less desirable, they indicate a lower reliability of the solubility prediction. That indication can informatively discriminate samples through the test set, and it would not be available with a non-normalized NC measure (AR) which produces a constant absolute residual for the whole set. Figure 3b–d display top CP intervals (blue line) at the 90% confidence level of the Ethanol, Acetone, and Water-wide data sets in pair with their experimental Log(S) (red dots). The 95% and 99% confidence levels would only produce linearly wider intervals for every test case. In praxis, one is not always interested in the general prediction performance of a validation set as a whole, but in the error margin of an individual solubility prediction. This is illustrated with 1-pentanol, which has an experimental Log(S) = −0.6, predicted Log(S) = −0.42 in water, and a normalized 90% confidence half-width interval of only 0.802. This means, in colloquial terms, that its solubility has been predicted more precisely, i.e., with a smaller error margin than with “conventional regression” [23], so that its 90% prediction interval is between −1.222 and 0.382. If a non-normalized constant interval half-width for all samples is used (i.e., AR) then its half-width would be 1.229, so the error margin would be between −1.649 and 0.809, i.e., wider by a factor of two. Another example is carbazole in acetone with an interval half-width of 0.75 Log(S). The true merit is that since prediction intervals are proven to be significantly correlated with absolute residuals, CP allows not only a more precise estimation but also a more accurate individual estimation of solubilities. However, note that the introduction of normalized CPs has nothing to do with a general increase in accuracy or predictions, as neither RMSE nor %LogS ± 1.0 is changed. Only individual error margins are introduced.

**Scheme 1:**
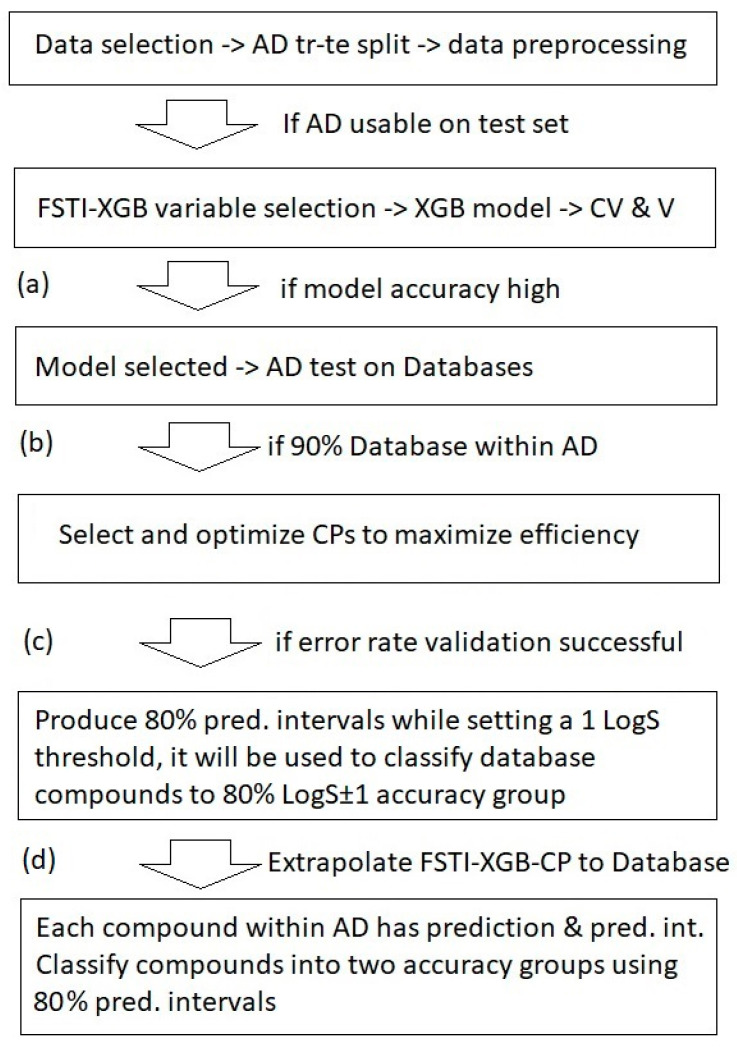
Research strategy from building AqSolDB models for solubility prediction to extrapolating them to external databases.

Scheme 1 displays our research strategy of informative solubility estimation of external databases. Concretely, after AD rules approve the train–test split, the first task (a) of the mandatory objectives is testing and proving model accuracy. The second task (b) is to test whether the AD of the training set covers the most compounds of external databases; usually if the data set contains too few compounds or if the training set is composed of a large number of too similar compounds, the second step already results into a weak AD cover of database compounds. The third point (c) is to obtain narrow prediction intervals using the “optimal” combination of CP and sensitivity coefficient, *β,* and then successfully validate these intervals using the error rate described in Section 3. If that validation is successful, only then can the informative extrapolation to the external database be carried out. It produces both predictions and prediction intervals for each compound of the database. The very last step (d) is the successfulness of the whole extrapolation, which is described in the subsequent subsection because it also depends on the database where built models are extrapolated. Other ML solubility references carry out the first task (a) and provide a detailed descriptor analysis and method discussion. But in most cases, they stop there or are oriented toward the AD assessment of relatively small test sets at best [36].

### 2.4. Extrapolation of AqSolDB Models to External Databases

**Figure 4:**
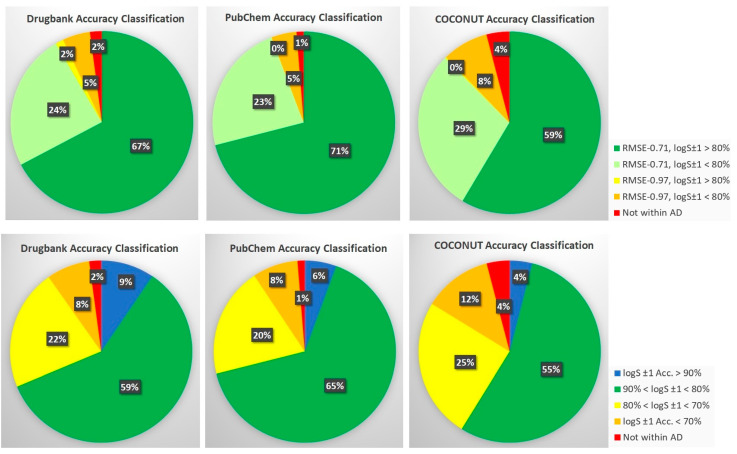
Accuracy classification. Upper pies only use an 80% confidence level to classify databases into two accuracy groups while also discerning the AqSolDB-n model from AqSolDB-w, giving four classes. Lower pies display statistics of more confidence levels according to the analysis in Info Section S4.

FSTI-XGB extrapolation results from AqSolDB-w (EM-N) and AqSolDB-n (ARSS) models to three databases are presented in Figure 4. The first three upper pies display the yields of these two models concerning the fraction of compounds that fall in and out of the LogS ± 1 > 80% accuracy statistic. Thus, altogether, there are 2 × 2 accuracy classes obtained. The lower three pies are merged results of two models while taking into account more confidence levels (not just 80%). Therefore, four accuracy groups are also obtained, satisfying these conditions: (1) logS ± 1 > 90% accuracy level, (2) 90% > logS ± 1 > 80%, (3) 80% > logS ± 1 > 70%, (4) logS ± 1 < 70%.

**Figure 5:**
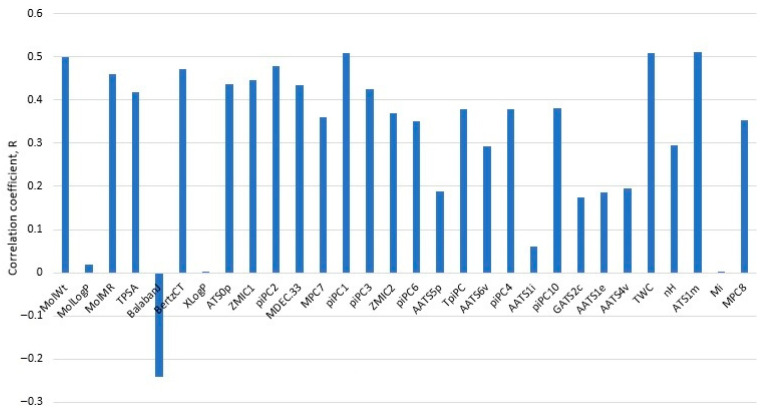
Pearson’s R correlation coefficient between produced prediction intervals of the COCONUT set (356,277 compounds within AD) and 26 AqSolDB-n ML model molecular descriptors plus MolWt, TPSA, BalabanJ, and BertzCT (these four mentioned were not part of the FSTI-XGB model but are still correlated).

The general accuracy statistics are presented in Table 8. The total size column gives the overall number of considered compounds (within the AD and outside the AD), and the column to the left lists median molecular weights for the data sets. The “In AD Assessed” column for AqSolDB-n presents the number of compounds within its AD for which calculated statistics are displayed in the next three columns to the right. For example, 0.888 represents the median prediction interval half-width at the 80% confidence level for 10,413 compounds. Next, 73.6% is the fraction of Drugbank compounds in AqSolDB-n AD that fall within the LogS ± 1.0 > 80% accuracy group (i.e., 7649 compounds). That is not a fraction of all Drugbank compounds, which is 67% (Figure 4). Moreover, 73.6% is obtained in a per mill analysis of the prediction interval as the top 736th per mill had a 1 Log(S) half-width at the 80% confidence level. The last column is the total estimated accuracy of 10,413 Drugbank compounds assessed by the AqSolDB-n ML model. It is calculated using a detailed percentile (and per mill) calculation fully elaborated in Info Section S4. In a few sentences, the task was to determine the percentiles at which interval half-widths were almost equal to 1 Log(S) at different confidence levels. Besides the 80% confidence level, 90%, 70%, and other confidence levels were considered too. In total, 96% of AqSolDB-n had narrower prediction intervals than 1 Log(S) at a 70% confidence level. Thus, the accuracy rate of 70% was valid for at least 96% of the compounds. Similarly, the accuracy rate of 80% was valid for at least 73% of the compounds, and the accuracy rate of 90% was valid for at least 9% of the compounds. The accuracy rate of 92% was valid for 4% of the compounds. This means that 64% of the compounds (the difference between the 73% and 9% fractions with accuracies of 80% and 90%, respectively) had an average accuracy between 80% and 90%, which was approximated to be 85% LogS ± 1. Similarly, for 23% of the compounds (difference between 96% and 73%), the average accuracy was ca. 75% LogS ± 1, and 5% of the compounds had an average accuracy of 91% LogS ± 1. The linear combination of the average accuracies and fractions was then 100% × (0.91 × 0.05 + 0.85 × 0.64 + 0.75 × 0.23)/(0.05 + 0.64 + 0.23) = 82.8% (Table 8). Similarly, this was calculated for PubChem and COCONUT (Info Section S4). That is, for AqSolDB-n, the AqSolDB-w ML model only worked with the compounds that were not covered by AD of AqSolDB-n. The AqSolDB-w model assessed 717 Drugbank, 3273 PubChem, and 34,061 COCONUT compounds whose median weight was higher than 500 Da. It is no wonder why the LogS ± 1.0 > 80% accuracy group was very small for AqSolDB-w. The final result for AqSolDB-w was obtained by searching at which confidence level the mean interval half-width equalled 1 Log(S). The last row presents the linear combination of two ML models and their accuracies (e.g., for Drugbank (10,413 × 82.8% + 717 × 63.7%)/(10,413 + 717) = 81.6%). It can be seen that the final accuracy for all three studied databases was approximately 80%. This result is actually very satisfactory when taking into account that the median molecular weights of the AqSolDB-n and AqSolDB-w data sets were 1.6–2.1 factors lower than the studied databases, although prediction intervals were established to be significantly correlated with the molecular size (Figure 5 and Figure S4).

The correlation analysis between the prediction interval values and molecular descriptors of all COCONUT compounds within the AD is presented in Figure 5. That analysis proves that prediction intervals are positively correlated with molecular weights (Figure S4) [28] but also negatively correlated with the BalabanJ descriptor. We have already stated in our prior section (Section 2.3) that any individual molecular descriptor is less correlated with absolute residuals than prediction intervals are correlated with absolute residuals. This both explains the difference in the successfulness of the prediction of different weighted data sets and at the same time proves the merit of the production of prediction intervals using normalized CPs. With normalized CPs, it is possible to classify compounds into different accuracy classes. That is why normalized CPs are necessary for the informative assessment of solubility prediction for each compound in the database extrapolation set. In other words, without CPs, one would simply extrapolate FSTI-XGB to compounds within the AD and claim the accuracy of the obtained predictions to be the same as its internal (or external) test set. This study proves that such a claim would not be correct as the training data have 1.7–2.1 factors less weighted compounds and the accuracy rate for heavy-weighted databases is accordingly lower than for its internal or external data sets. In that context, we just recall about the three points raised in Section 1, as we were right: we established the importance of CPs for the solubility estimation of public databases and produced 473,276 solubility values, along with corresponding prediction intervals and accuracy classes of three analysed data sets.

**Scheme 2:**
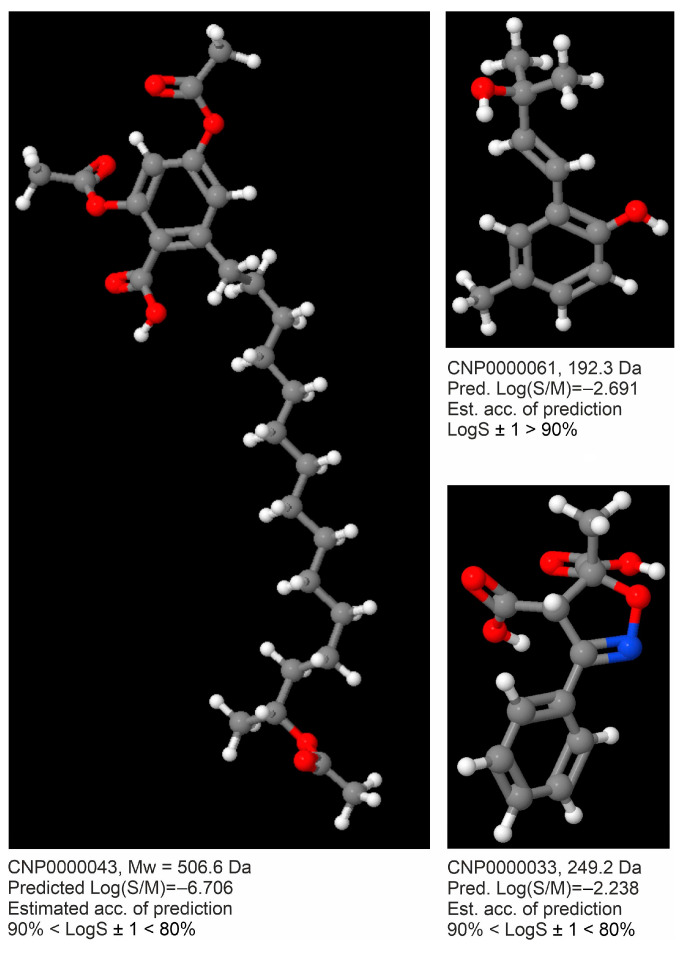
Final results for three selected COCONUT compounds. See Supplementary_databases_predictions.xlsx.

We put all database predictions in the Supplementary_databases_predictions.xlsx file, which contains the ID of the compound, SMILES, molecular weight, and four prediction columns, namely, Log(S/M) as the fourth column, and the ML model built on either AqSolDB-n (Aq-n) (RMSE = 0.71 LogS) or AqSolDB-w (Aq-w) (RMSE = 0.97 LogS) as the fifth column. In the sixth column are the obtained 80% prediction interval half-widths, while the seventh and last column gives the four accuracy classes according to the lower pies presented in Figure 4. For example, compounds with prediction intervals below 1 Log(S) at a 90% confidence level fall in the strongest acc. > 90% class, while those with intervals above 1 Log(S) at a 70% confidence level fall into the weakest accuracy group labelled with acc. < 70%. Scheme 2 displays the most important simplified form of our final results for three COCONUT compounds.

Although the exact RMSE after extrapolating cannot be established, the rough estimation of RMSE for public databases can be made if the produced LogS ± 1 (>80%) is compared with AqSolDB-w’s LogS ± 1 of 80% which has RMSEP = 0.962 Log(S); then the obtained RMSE of high-molecular-weight databases is likely slightly below 1.0 Log(S) unit. The result is remarkable since for intrinsic solubility prediction, the external test set span was 0.89–1.05 Log(S) [36], and our estimates were likely within that range. SolTranNet fast solubility prediction of less-weighted AqSolDB compounds obtained RMSECV = 1.459 and RMSEP = 1.71 [3]; in that context, our result proves how important XGB can be both individually and combined with the AD and CP for external databases’ solubility prediction. We can also compare our predictions with the already presented AlogPS [32] in the Drugbank database. FSTI-XGB’s Log(S) predictions follow AlogPS’s predicted Log(S) with *R*^2^ = 0.7587 (Figure S5) for 10,861 Drugbank compounds. These solubility estimations for natural compounds in the COCONUT database might pave the way for the future recognition of new drugs.

## 3. Materials and Methods

### 3.1. Data Sets, Data Curation, and Preprocessing

In the current study, we used our own (1) Methanol data set of 135 experimental solubilities derived from our experimental measurements and five already utilized experimental solubility data sets from Refs. [12,48]. These are: (2) The AqSolDB-wide (AqSolDB-w) data set consisting of 9709 noncurated compounds with at least four heavy atoms SMILES string of the largest contiguous fragment (heavy atom count (HAC) > 3) and composed of nine smaller data subsets with all experimental solubilities in water [48]. HAC < 4 substances were discarded due to many missing values produced when calculating molecular descriptors, and external databases have plenty of heavy-weighted compounds for which HAC < 4 substances certainly would not be useful when building an ML model for prediction. (3) AqSolDB-narrow (AqSolDB-n) with HAC > 3 consisting of 1619 curated compounds with two or more experimental solubility values of standard deviation (SD) < 0.5 Log(S) and having strictly one molecular fragment, i.e., all 149 compounds of AqSolDB-n with two or more fragments were discarded, because, except for the Drugbank database having ca. 4% more-than-one-fragment compounds, neither PubChem nor COCONUT database set contained more-than-one-fragment compounds. We wanted to prevent any potential negative influence of using the largest compound’s fragment SMILES on the accuracy of our models when predicting Log(S) of more-than-one-fragment compounds. (4) The Water set as “Water_set_wide” taken from Ref. [12], consisting of 900 water compounds. (5) The Ethanol data set comprising 695 experimental solubilities in ethanol taken from Ref. [12]. Finally, (6) the Acetone data set having 452 experimental solubilities in acetone taken from Ref. [12]. The input for the dependent variable for data sets (2–6) was Log(solubilities in mol L^−1^), while for data set (1) was Log(solubilities in g L^−1^) with a Log(S/g) range of −2.2–(+3). Log(S/M) ranges for data sets (2–6) were: (2) −13–(+2), (3) −12–(+2), (4) −12–(+2), (5) −4–(+1), and (6) −4–(+1). We treated each data set independently and separately, so for the sake of simplicity, we continued to generally refer to these quantities as Log(S) (in later text). All data sets were used for the accuracy testing of our XGB methodology and for the conformal prediction, and every data set had a training set for the cross-validation (CV) and an independent test set. The large data sets (2, 3) were also used for the solubility prediction of external databases. Also, the whole Water data set (4) was utilized as an external test set for data set (3).

The preprocessing included removing all variables with a missing value, infinitive value, and only one unique value. All the variables were scaled and mean-centred, which was performed according to the training data.

#### 3.1.1. AD for Train–Test Split

The AD was defined using Ref. [37], which defines the AD for quantitative structure–activity relationship (QSAR) studies [36]. In short words, after data preprocessing, the AD was defined only by the training set by exactly following five steps in Ref. [37] regarding training a *k*-nearest neighbours model. In doing so, 17 scaled AqSolDB rdkit variables were considered to check the AD between the training set and test set for all (1–6) data sets as initial guess variables. Later, after the XGB variable selection method determined optimal descriptors on already AD-established training sets, the AD for the test sets was retested with such scaled XGB descriptors and confirmed the prior results of 17 AqSolDB variables for all six data sets. Scaled XGB-variable-selected descriptors were used for the final AD coverage between data sets (2–3) and large databases. To determine whether the test set compound was inside the AD, its scaled Euclidean distance from all the training samples was calculated and simultaneously compared to be less than or equal to the priorly calculated training set thresholds defined in Ref. [37]. If this condition was true with at least one training sample, then the test sample was considered to be inside the AD for that train–test split. Otherwise, the prediction for that test sample was outside the AD. Parameter *k* was optimized in this study to be 37.5% of the training set as that resulted in the least outliers for AqSolDB-w and was very close to the percentage determined to be optimal in Ref. [37]. The train–test split was conducted in way of the well-organized sequential split of initially taken input data sets with the AD test carried out after the splits. The selected sequential train–test splits for data sets (1–2,4–6) were approved if less than 0.5% of test samples were outside the AD. For data set (3) it was approved if both (a) less than 0.5% of test samples were outside the AD for parameter *k* being only 5% of the training set, and (b) all test compounds were within AD for *k* = 37.5%. For our data set (1), the correct split was selected from the second attempt with 99 training and 36 test compounds. For data set (2), there were 8091 training compounds and 1618 test validation compounds, so each sixth compound starting from the second compound was used as the test compound. Seven compounds were outside the AD and a later analysis revealed only a slightly statistically negative impact on accuracy indices (more details in the Section 2), but the equal-variance *t*-test on the squared residuals between these 7 compounds and the remaining 1611 was insignificant (*p* > 0.05), so we decided to keep them at that time in the test set. For data set (3), 1399 training samples and 220 test compounds were selected on the eighth attempt. Each seventh compound starting from the seventh was a middle set, the other 6/7 were training, and from the middle set, each twenty-second example starting from the eleventh was also a training compound, while the rest were test compounds. For data set (4), every ninth compound starting from the second, the fourth, the sixth, and the eighth were test compounds, and all compounds were within the AD, so that 500 training samples and 400 test compounds were selected, and all test samples were within the AD. For data sets (5) and (6), each fifth sample starting from the second was taken as a test for performance modelling estimations, so set (5) had 139 and set (6) 91 test cases. All test compounds were within the AD. All data sets and training–test split details are provided on the GitHub repository, s (accessed on 29 November 2023), and in detail in file Supplementary_data_sets_table.xlsx.

#### 3.1.2. Molecular Descriptors

Molecular variables Padel 1D and 2D descriptors (altogether 1444 variables) [52] and 17 rdkit descriptors (in the later text all these variables are regarded as “Pvars”) for all six data sets were calculated on the largest contiguous fragment SMILES which can be found for each data set in Supplementary_data_sets_table.xlsx on the GitHub repository (see also Supporting Info Section S5 of Supplementary_documentation.pdf on calculation instructions). Besides Padel descriptors, Padel Fingerprints (16092 variables) were also taken into account but did not improve the model performance for data sets (1, 3–6). For data set (2), XGB preselected Padel fingerprints using a Boruta selection of variables. These were accounted for by including the top 75% of important variables and were utilized only for all-variable RF and XGB methodologies. Thus, in addition to preprocessed Pvars, 623 additional Padel fingerprints were added to data set 2. All variable names ordered for the input of data set (2) (AqSolDB-w) can be found on the GitHub repository, https://github.com/ojovic985/XGB-solubility (accessed on 29 November 2023). For data set (1), we obtained the melting points, fusion enthalpies, molecular weights, and number of donor and acceptor atoms from Pharmacopoeia [53]. We also calculated the solvation Gibbs free energy using the continuum implicit solvation model of density (SMD) [54] in the ORCA DFT program [55] (see Supporting Info Section S6 for code details). We obtained a total of 13 physicochemical descriptors “QMvars” (Supporting info Section S7) for data set (1). For data sets (4–6), a list of 41 physicochemical descriptor set “QMvars” (Info Section S8) derived from quantum-mechanical computations were downloaded from Ref. [12] and used for the data sets. For data sets (1, 4–6), all QMvars files are stored in the given GitHub repository as methanol_set_descriptors.csv, water_set_wide_descriptors.csv, acetone_set_descriptors.csv, and ethanol_set_descriptors.csv.

### 3.2. Machine Learning

The methodologies used for the minimization of the root-mean-square error of the CV (RMSECV) were an RF [49,56], XGB in R [57], and our variable selection algorithm using XGB. The fine-tuning of the RF was conducted with the command “tuneRF” and input parameters ntreeTry = 500, stepFactor = 10, improve = 0.05, trace = TRUE, and doBest = TRUE. We also considered a non-fine-tuned RF with the command “randomForest” and input parameters forest = TRUE, confusion = TRUE, ntree = 500, and mtry = 100. For XGB, the normal grid search optimization was carried out using “xgb.cv” and “max_depth” between 1 and 6 with one as an increment, the “eta” parameter between 0.1 and 1 with 0.1 as an increment, and the number of rounds up to 200 for data sets (1, 4–6). A finer grid search included 60 equidistant eta values between 0 and 0.4 for all data sets except the Methanol set (1) for the NC estimation with 99 training samples. For data set (2), the number of rounds utilized was up to 600 when using a finer grid search. For data set (3), 300 rounds were used. For both RF and XGB, a 20-fold CV was performed.

#### 3.2.1. Variable Selection Procedure for XGB

1. Upon obtaining a grid-optimized XGB model on all variables, sort out the descriptors based on their statistical importance in decreasing order.
2. Starting from the top three variables, perform a 5-fold CV in each step by parallelly adding the next top important variable in the forward stepwise (one by one) manner, and limit such forward addition to the top *m* variables (*m* = 30). Use the normal grid search in XGB for each addition of a variable.
3. After adding *m* variables, continue the forward stepwise addition until RMSECV(new step) > RMSECV(ex-step), then stop and select the number of variables of the RMSECV global minimum.

The selected variables can then be used to build a new XGB model. This is the forward stepwise top-importance XGB (FSTI-XGB). It was carried out after the proof of all-variable XGB outperforming the all-variable RF (see later). Otherwise, if the RF had yielded a higher accuracy, we would have used an analogous methodology for the RF instead of XGB. FSTI-XGB-selected variables were then used to build a new XGB model which was further parametrized using the finer-grid-based method and with a 20-fold CV. For data set (3), between point (1) and point (2), a 0.9 correlation threshold was also considered to exclude all mutually correlated descriptors in the top-importance-sorted descriptor order, and instead of 30, at most 40 descriptors were considered.

When the hyperparametrized model having the minimum RMSECV was achieved with a 20-fold CV, the 20-fold CV sample split was saved and used for the conformal prediction calculation. In addition, the root-mean-square error of the validation on the test set (RMSEV) was calculated, together with R^2^ values for the cross-validated training set (CV-set) and the test validation set (V-set) using experimental Log(S). Accuracy measures already considered besides RMSE and R^2^ were %Log(S) ± 0.7 and %Log(S) ± 1.0, where %Log(S) ± 0.7 represents the maximum accuracy of the model based on the available data [12].

#### 3.2.2. Conformal Predictors

Conformal predictors (CPs) are models that associate each of their predictions with a measure of confidence. For a test sample and significance ε, a conformal predictor (CP) outputs a prediction region containing the target test sample with probability 1 − ε. For regression, the prediction region is the prediction interval containing the predicted value at the centre of the interval [22]. The efficiency of a CP is inversely proportionally related to the half-width of the prediction interval [21,24]. Nonconformity measures (NC) assess the degree to which the new test sample deviates with its attribute–label relationship from the old examples.

The non-normalized NC measure is a simple absolute residual (AR) of a certain percentile for the corresponding confidence half-width region [21]:

(1) $$AR=\left|y-\hat{y}\right|$$

The normalized NC is then the absolute residual divided by the normalization value sigma (σ), which is an estimate of the difficulty of predicting solubility. A sensitivity parameter *β* is usually added to *σ* so that we obtain the equation for a normalized NC measure (Equation (2)):

(2) $$\alpha =\frac{\left|y-\hat{y}\right|}{\sigma +\beta }$$

For a cross-conformal predictor [58], using *k* folds the standard deviation of more predictions for a test sample can also be used as a normalization value, σ [21,22]. For any regression, if one repeats any fold CV *n* times, one obtains *n* cross-validated predictions for each calibration example. Then, one simply divides the AR (or the mean of the AR) by the standard deviation of *n* predictions for each sample. By dividing the AR by the standard deviation of the residuals, one obtains the absolute residual stability (ARS).

The term “stability” has already been similarly used in uninformative variable elimination regression algorithms, where each regression coefficient (i.e., the mean) of the regression vector is divided by the standard deviation of its varying values obtained by CV [59,60]. For the NC measure, either the mean of *n* samples or one single value can be used in Equation (2), which defines two different but similar CPs. For the ARS, the mean is used. The variant of the ARS instead of the mean has a single value in the numerator and is called absolute residual semistability (ARSS).

##### Absolute Residual (AR)

From the saved hyperparametrized model having obtained the minimum RMSECV from the former section, the absolute residuals were utilized for all calibration samples and sorted in descending order. The 99th percentile of absolute residual produced a 99% half-width prediction interval for all calibration samples, similar to the 95th percentile for a 95% half-width, 90th percentile for a 90%, and 80th percentile for an 80% prediction interval. These were also utilized for the AR when assessing the frequency of test samples’ absolute residuals being outside of the half-width prediction intervals.

##### Absolute Residual Stability (ARS) and Absolute Residual Semistability (ARSS)

A twenty-time-repeated 20-fold CV was conducted on all training samples so that every CV training sample had 20 predictions used to obtain the mean *μ* and standard deviation. Equation (3) defines the absolute residual stability (ARS).

(3) $$\alpha (ARS)=\frac{\left|y-\hat{\mu }\right|}{\sigma +\beta },$$

where in this case, σ = *s*.

(4) $$s=\frac{\sum_{i}^{k}\left(\hat{\mu }-\hat{y_{i}}\right)}{k-1},\hat{\mu }=\frac{\sum_{i}^{k}\hat{y_{i}}}{k}$$

*β*, as the sensitivity coefficient, can be adjusted to reach optimal efficiency for the analysed data set. It is usually equal to 1 [23] or 0.5 [22] for the normalized data having all the attributes between −1 and 1 [61]. In this work, such prenormalization was not applied because that would have meant having some preknowledge on independent test sets. What if the maximum value of the test set for any considered molecular descriptor is above the maximum of the training set? The test value would have to be higher than 1. Therefore, instead of doing such a normalization, we considered values of *β* relative to the median of all calibration sample estimates of σ.

Equation (3) is different from Equation (2) in the numerator. Instead of *μ* for 20 predictions, only 1 prediction could be used. In that case, the absolute residual from Equation (1) is only divided by the standard deviation of the CV predictions, which defined the absolute residual semistabilities (ARSS):

(5) $$\alpha (ARSS)=\frac{\left|y-\hat{y_{1}}\right|}{\sigma +\beta },\sigma =\frac{\sum_{i}^{k}\left(\hat{\mu }-\hat{y_{i}}\right)}{k-1}$$

α(ARS) and/or α(ARSS) are normalized NC measures that can be obtained for every training sample in the cross-conformal prediction. For cross-conformal predictors, all training samples are also calibration samples [58]. When α(ARS) for all calibration samples were obtained, these were then sorted in descending order, and the 99th, 95th, 90th, and 80th percentiles were taken as referent NC measures. The 20-fold CV training split obtained in the former section for the hyperparametrized XGB was used for a 20-model prediction to calculate 20 Log(S)’s for each test sample. The standard deviation of these 20 predictions defined σ_TEST_ for every test example. Obtaining σ_TEST_ with these 20 models, each using 95% of the training set, had nothing to do with one “ordinary” test prediction using 100% calibration samples at once, which predicts Log(S). Both were performed without the knowledge of the true (i.e., experimental) test set solubilities. When referent NC measures were multiplied by each correspondingly obtained test sample’s denominator of Equation (3) (i.e., *β* + σ_TEST_), they produced 99%, 95%, 90%, and 80% half-width prediction intervals for every test sample (Equation (6)).

Test Interval half-width (99% conf) = (*β* + σ_TEST_) × *α*(ARSS) (99th perc.) (6)

**Scheme 3:**
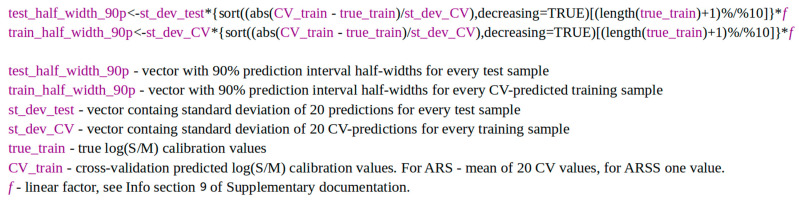
Equations in an R program for producing ARSS, in this case, 90% prediction half-width intervals for test and training samples’ solubilities. The number in {} brackets is normalized the NC. (Simbol above (*) in R program represents multiplication (×) simbol).

Such a half-width is then added and subtracted at the same time from the corresponding test sample’s prediction so that the final prediction interval has a full width of 2× half-width with the test sample’s prediction in the centre of the prediction interval. For details on how these NC measures were obtained with certain confidence levels concerning already described data sets of the specific number of calibration samples, see Info Section S9. See also Scheme 3.

##### Error Model and Log-Error Model

Besides the ARS, the already used CP for a drug-like property is the error model (EM) [23]. After obtaining the AR, the EM builds a new model for the prediction of ARs as a new dependent variable using the same independent variables. The NC for the EM is the AR divided by such an EM prediction. Instead of predicting absolute residuals, one can also predict the logarithm of absolute residuals [23] or obtain the most efficient variant of EM CP.

Using preoptimized XGB hyperparameters, the first 20-fold CV run was carried out to produce absolute residuals. These absolute residuals were further utilized as a new dependent variable (instead of the initial Log(S)). Thus, the second 20-fold CV was performed using the same descriptor-independent variable matrix on the new dependent variable [23]. This produced error predictions for all training and test samples, as the second 20-fold CV sample split was saved and also used on the test samples. This procedure was repeated 20 times, producing 20 error predictions for each training sample and 400 predictions for each test sample. Then, the mean of the absolute residuals produced after the first CV run was divided by the term (*β* + σ), where σ is the mean of the residuals of the second run for each NC measure (Equation (7)). The obtained NC measures on the whole training (i.e., calibration set) were sorted in descending order and the 95th percentile was used for the production of the 95% prediction interval. The error model half-width prediction interval for each test sample was obtained when the 95th percentile NC measure was multiplied by the corresponding (denominator) term (of Equation (7)) calculated for the test set samples.

(7) $$\alpha (EM\text{-}N)=\frac{\left|y-\hat{\mu }\right|}{\sigma +\beta },\;\sigma ={\hat{\mu }}_{E},$$

$${\hat{\mu }}_{E}=\frac{\sum_{i}^{k}{\left(\hat{y_{i}}\right)}_{E}}{k}$$

As in the ARS, *β* can be optimized for higher efficiency. The term EM-N means an EM for a normal function. Similar to EM-N, there is an EM-Log CP which uses the same procedure, except for the second step, where instead of a prediction of the absolute residual for the dependent variable, one predicts the logarithm of the absolute residual. Later, σ is equal to the mean of these exponential (Log) predictions (Equation (8)).

(8) $$\alpha (EM\text{-}Log)=\frac{\left|y-\hat{\mu }\right|}{\sigma +\beta }$$

$$\sigma =\frac{\sum_{i}^{k}e^{\hat{{\left(yi\right)}_{logE}}}}{k}$$

The exponential EM (EM-Exp) was considered too, but only related to the end-predictions in both steps of EM-N, not in predicting the exponent of the absolute residual in the second CV step. In addition to the mean of the numerator and σ in Equation (7), the standard deviation was also considered. But these two additional NC measures taken into account attained less efficient, wider intervals. EM-N and EM-Log were applied to all data sets combined with FSTI-XGB. See also EM-N_code.R in the GitHub repository for how the EM-N prediction interval half-widths were produced.

##### k-Nearest Neighbours from Euclidean Distances (kNN-EuD)

Attempts to use other normalized NC functions such as *k*-nearest neighbours (kNN) [22] were made on FSTI-XGB-selected molecular descriptors. These were used to calculate mutual scaled Euclidean distances (kNN-EuD) between each calibration sample and its closest *k* training samples in the 20-time 20-fold CV-loop. Equation (9) was used where the definition of σ included the average of 20 cross-validated calibration values for the mean Euclidean distance. Except for the mean, the sum and the standard deviation were also explored, but the mean was the most efficient.

(9) $$\alpha (kNN\text{-}EuD)=\frac{\left|y-\hat{\mu }\right|}{\sigma +\beta },\;\sigma ={\hat{\mu }}_{EuD},$$

##### RMSE × t-Value

We also considered the definition of prediction interval half-width as a *t*-value at the corresponding significance level and degrees of freedom times the RMSECV [62,63]. However, such a definition led to cases with a significant number of misclassifications on the AqSolDB-w set (*t*-test, *p* < 0.001).

#### 3.2.3. Validation of Prediction Intervals

After building the prediction intervals, the error rate was calculated. The error rate for every confidence level is defined as the number of predictions that fall outside of prediction intervals (i.e., misclassifications). Cases when the error rate exceeds the significance level threshold can be expected, especially on small data sets. Yet, they are not expected if the error rate is significantly higher than the significance threshold. To measure that, an equal variance *t*-test was carried out using two vectors with dummy variables (0—inside, 1—outside interval). Two vectors of the same test sample size between the predicted and the expected error rate vector were tested. If *p* < 0.01, it meant that the methodology failed on validation for that combination of CP, *β* value, and confidence level.

### 3.3. Extrapolation to the Drugbank, PubChem and COCONUT Databases

The latest “structure links.csv” (4 January 2023) file was downloaded from Drugbank [26]. It contained 12,227 compounds among which 11,583 had SMILES. In total, 212 SMILES files were structures with either a HAC lower than four for the largest fragment, or they had valence atom issues. Thus, the FSTI-XGB model on AqSolDB was used to predict Log(S) for 11,370 Drugbank compounds for which Pvars were calculated. The best combination of CP function and *β* value was used to compute the prediction intervals. A similar FSTI-XGB-based AqSolDB extrapolation using canonical SMILES was conducted for PubChem crystal structures [33] and the COCONUT database [34], where log(S) and prediction intervals were calculated for 72,739 PubChem and 406,919 COCONUT compounds. The AD rule using Ref. [37] and already described in Section 3.1.1 was carried out to exclude all compounds from databases that did not satisfy it for both FSTI-XGB-variable-selected training sets of AqSolDB-w (2) and AqSolDB-n (3). See final results in Section 2.2.

## 4. Conclusions

Here, we summarize our contributions:

(1) The FSTI-XGB model, having at most 34 descriptors, outperformed in accuracy five different data sets which included two ML studies of uncurated and curated AqSolDB sets. The obtained accuracy for the Acetone data set could be put in line with other top-performing non-TL Log(S) prediction results. For the AqSolDB-n data set, the obtained total CV and test accuracy (RMSEtot) was lower than any water Log(S) CV literature train(all-CV)–test(all-independent) split’s RMSEtot known to date, if very small independent test-size data of ca. 20 compounds and CV results on all data sets as “final tests” are neglected. It was obtained only with Pvars, which underperformed when QMvars (e.g., melting points) were also available (Table 3). Additionally, we proved that XGB was a stronger ML method than RF, and that FSTI-XGB correctly selected important descriptors.
(2) For the first time, conformal predictors were extensively calculated to produce prediction intervals for Log(S) in water and organic solvents using the XGB algorithm. This study revealed that they could be utilized to approximatively assess the important %LogS ± 1 accuracy measure of the data set. The calculations was feasible with the ARSS when *β* was targeted between 0.5 and 1.5 median(σ) if the data of molecular descriptors were scaled when used to predict Log(S). The ARSS significant interval efficiency gain was successfully validated on experimental solubility data sets (1–6) and on one external test set (900), ranging from 135 examples (data set (1)) to 9709 cases (data set (2)).
(3) The normalized half-width intervals were not only correlated with the absolute residuals but were more correlated with the absolute residuals than any single molecular descriptor in the model. That information marks the background support of CP application to the informative solubility estimation of any targeted compound. The informativeness of varying individual half-width intervals offers either reliable estimates of individual Log(S) for narrow intervals or at least carries an indication of less reliable estimates for the cases of wide intervals. This is important for individual compound Log(S) estimation in the process and product development of potential new drugs and with regards to any solvent.
(4) We predicted 473,276 Log(S)’s (11,130 DrugBank + 71,808 PubChem + 390,338 COCONUT) values, evaluated their individual error margins, and classified them into four accuracy groups. We also assessed the approximative general accuracy of the databases. The obtained final estimated LogS ± 1 was in the range 79.9–81.6% while the RMSE for the three databases was <1.0 Log(S), which was very solid when considering the difference in molecular weights between our model data set and public domains.

Compromises between the accuracy and AD could not be fully covered, because this article had to introduce CPs to sharply show their contribution to Log(S) solubility estimation. Future perspectives might consider utilizing expert outlier analysis to further enhance accuracy while not impacting AD coverage significantly. That might be one direction. The second direction might work with very recently emerging TL methods for solubility prediction by combining them with the AD and CP, if possible, for a potential investigation of additional model improvements.

Predicted water solubilities of Drugbank compounds, PubChem crystal structures, and natural compounds (COCONUT) can be important for new drug recognition studies.

## Figures and Tables

**Table 1:** The comparison of XGB and RF methods on AqSolDB-n (size of 1619) training (CV) set (1399) plus internal independent validation (V) set (220), and Water-wide external test (T) prediction set (900) on a total of 532 descriptors.

**Method**	**RMSECV**	***R*** **^2^** **(CV)**	**CV-%LogS** **± 0.7**	**CV-%LogS** **± 1.0**	**RMSEV**	***R*** **^2^** **(V)**	**V-%LogS** **± 0.7**	**V-%LogS** **± 1.0**
RF	0.757 *	0.890	73.5	84.3	0.596	0.929	77.7	90.9
RF fine-t.	0.753 *	0.890	73.8	84.5	0.594	0.929	79.5	90.0
XGB	0.711	0.901	77.1	88.3	0.558	0.936	83.6	92.7
**Method**	**RMSEP (T)**	***R*** **^2^** **(T)**	**T-%LogS** **± 0.7**	**T-%LogS** **± 1.0**	**Method**	**RMSEtot**	**%LogS** **±** **0.7tot**	**%LogS** **± 1.0tot**
RF	0.790 *	0.899	72.1	84.4	RF	0.755	73.1	84.9
RF fine-t.	0.781 **	0.900	73.1	84.3	RF-fine-t.	0.749	74.0	84.9
XGB	0.755	0.905	75.2	86.0	XGB	0.713	77.0	87.9

* A statistically significant difference between RF model performance values with XGB, paired *t*-test, *p* < 0.01. ** *p* < 0.05.

**Table 2:** The comparison between XGB and RF methods on AqSolDB-w (9709) training (CV) set (8091) plus internal independent validation (V) set (1618) on a total of 1146 descriptors.

Method	RMSECV	*R*^2^(CV)	CV-%LogS ± 0.7	CV-%LogS ± 1.0	RMSEV	*R*^2^(V)	V-%LogS ± 0.7	V-%LogS ± 1.0
RF fine-t.	1.060 *	0.798	62.9	75.6	0.958 **	0.836	66.9	79.2
XGB	0.943 *	0.838	69.0	80.9	0.924 **	0.845	69.8	81.5

* A significant difference between the model performance values, F-test on residuals, *p* < 0.001. ** Paired *t*-test, *p* < 0.01.

**Table 3:** The comparison of the performance of FSTI-XGB using different variables (P or QM or P + QM), RMSECV as CV performance on the training set in Log(S), and RMSEV as test performance on the validation set.

Data Set, vars Type as P/QM/P + QM (Number of Variables)	FSTI-XGB	
	RMSECV	RMSEV
Methanol, P (17)	0.7007	0.8480
Methanol, QM (6)	1.0541 *	0.8471
Methanol, P + QM (34)	0.6893 *	0.7883
Ethanol, P (32)	0.7303	0.8923
Ethanol, QM (16)	0.7363 *	0.8727
Ethanol, P + QM (33)	0.6503 *	0.7902
Acetone, P (30)	0.7337	0.7969
Acetone, QMvars (12)	0.7097 *	0.6975
Acetone, P + QMvars (31)	0.6164 *	0.7018

* A significant difference in RMSECV between QMvars and P + QMvars for *p* < 0.05 (*t*-test on squared residuals).

**Table 4:** This work’s FSTI-XGB vs. result of Ref. [3] for AqSolDB-w and the top two results of Ref. [25] for AqSolDB-n.

Data Set	Set Size	Method	nvars	RMSECV/ RMSE-Test *	RMSEV	RMSEtot	*R*^2^(V) ** CV	%LogS ± 0.7, CV-Set/V-Set		%LogS ± 1.0, CV-Set/V-Set	
AqSolDB-w	9982	SolTranNet [3]	-	1.459	1.711	-	0.68 **	-	-	-	-
AqSolDB-w	9709	FSTI-XGB *	32	0.966	0.966	0.966	0.831	68.6	67.2	79.7	79.7
AqSolDB-n	1674	NN-A [25]	7	0.72 *	0.76	0.74 *	0.88	-	-	-	-
AqSolDB-n	1665	NN-D [25]	22	1.07 *	0.96	1.00 *	0.80	-	-	-	-
AqSolDB-n	1619	FSTI-XGB *	26	0.730	0.594	0.712	0.928	76.3	76.8	87.3	92.7
AqSolDB-n	1619	FSTI-XGB *	Ext. test set, RMSEP = 0.764, *R*^2^(T) = 0.902, %LogS ± 0.7 = 75.7, %LogS ± 1.0 = 85.3%								

AqSolDB-w FSTI-XGB: md 6, eta 0.06, nr 583; AqSolDB-n FSTI-XGB: md 5, eta 0.1067, nr 300. * In this work, all training samples were part of a CV, different from Ref. [25], where they used two internal (dependent) test sets. RMSEtot displayed (0.74 of NN-A and 1.00 Log(S) of NN-D) with regards to the average of their two test sets (see Info Section S2).** *R*^2^ obtained in cross-validation.

**Table 5:** This work’s FSTI-XGB vs. the top two (CV) results of Ref. [12] for the Water-wide, Ethanol, and Acetone data sets. The table also displays results on our own Methanol set, which seem to approximately follow other model performance values.

Data Set	Set Size	Method	nvars	RMSECV	RMSEV	RMSEtot	*R*^2^(V)	%LogS ± 0.7, CV-Set/V-Set		%LogS ± 1.0, CV-Set/V-Set	
Water-wide	900	SVM [12]	41	0.85	0.85	0.85	0.89	68.3	71.6	81.7	78.9
Water-wide	900	GP [12]	41	0.86	0.89	0.86	0.88	67.0	68.4	79.2	73.7
Water-wide	900	FSTI-XGB	33	0.779	0.833	0.803	0.883	70.4	70.5	83.2	82.2
Ethanol	695	RF [12]	41	0.76	0.79	0.76	0.53	68.5	64.8	82.2	82.4
Ethanol	695	Bag [12]	41	0.76	0.80	0.77	0.52	69.1	65.5	82.7	79.6
Ethanol	695	FSTI-XGB	33	0.650	0.790	0.678	0.528	78.1	67.6	89.7	82.0
Acetone	452	SVM [12]	41	0.69	0.84	0.72	0.42	76.3	72.8	84.3	81.5
Acetone	452	Bag [12]	41	0.70	0.83	0.72	0.41	73.7	62.0	84.9	80.4
Acetone	452	FSTI-XGB	31	0.616	0.702	0.633	0.558	78.9	71.4	88.9	83.5
Methanol	135	FSTI-XGB	34	0.689	0.788	0.715	0.524	70.7	66.7	84.8	86.1

Ethanol FSTI-XGB: max_depth (md) 3, eta 0.1467, nrounds (nr) 198; Acetone FSTI-XGB: md 2, eta 0.14, nr 133; Water FSTI-XGB: md: 4, eta 0.0733, nr 198; Methanol FSTI-XGB: md 2, eta 0.1, nr 199.

**Table 6:** CP results for FSTI-XGB model with 32 variables, presented as mean/median half-width intervals in Log(S).

Method		99%		95%		90%		80%		Average of 8 Statistics	Average of All 4 Means
CP	β	Mean	Median	Mean	Median	Mean	Median	Mean	Median		
AR	-	3.439	3.439	2.043	2.043	1.489	1.489	1.006	1.006	1.994	1.994
ARS	0	3.193	2.733	1.856	1.588	1.425	1.220	1.033	0.885	1.742	1.877
ARS	0.2	2.906	2.720	1.751	1.639	1.350	1.264	**0.959**	0.898	1.686	1.742
ARS	0.5 × Q2(σ)	2.905	2.612	**1.717**	1.544	1.364	1.226	0.977	0.879	1.653	1.741
ARSS	0.5 × Q2(σ)	2.806	2.524	1.744	1.568	1.390	1.250	0.993	0.893	1.646	1.733
EM-N	0	2.811	2.359	1.830	1.536	1.428	1.198	1.031	0.866	1.632	1.775
EM-N	0.5 × Q2(σ)	**2.657**	2.355	1.735	1.537	**1.350**	1.196	0.971	0.860	1.583	1.678
*EM-N*	*0.3* × *Q2(σ)*	*2.667*	*2.323*	*1.742*	*1.517*	*1.360*	*1.185*	*0.987*	*0.860*	*1.580*	1.689
EM-Log	0.5 × Q2(σ)	2.734	2.461	1.761	1.585	1.350	1.215	0.984	0.886	1.622	1.707
knn-EuD	0	3.454	2.815	2.167	1.766	1.633	1.331	1.125	0.917	1.901	2.095
knn-EuD	1× Q2(σ)	3.123	2.805	1.968	1.768	1.489	1.768	1.022	0.918	1.858	1.901

Bolded value is the minimum mean value in the mean column. Italic values represent minimum average of 8 statistics among all other methods in rows.

**Table 7:** FSTI-XGB’s prediction interval half-width means and medians (Q2) of the most efficient CP at a given confidence level for all data sets (**1**–**6**) vs. AR (see Table S2). Each first row represents CP and efficiency gain as AR/CP(Q2).

Confidence Level:	99%	95%	90%	80%
AqSolDB-w, AR(Q2), AR/CP(Q2)	3.439, 48.0%	2.043, 32.3%	1.489, 20.3%	1.006, 12.0%
CP, CP mean, CP(Q2)	EM-N, 2.657, 2.323 *	ARS, 1.717, 1.544 *	EM-Log, 1.342, 1.238 *	ARS, 0.959, 0.898 *
AqSolDB-n (int. t. set), AR, AR/CP	2.335, 32.9%	1.534, 27.9%	1.176, 18.5%	0.783, 7.1%
CP, CP mean, CP(Q2)	EM-N, 1.831, 1.757 *	EM-N, 1.267, 1.199 *	EM-N, 1.034, 0.992 *	ARSS, 0.755, 0.731 **
AqSolDB-n (ext. t. set), AR, AR/CP	2.335, 24.3%	1.534, 23.9%	1.176, 17.7%	0.783, 6.1%
CP, CP mean, CP(Q2)	ARS, 1.935, 1.878 *	ARS, 1.276, 1.238 *	ARSS, 1.029, 0.999 *	ARSS, 0.751, 0.738 **
Water-w (int. t. set), AR, AR/CP	2.367, 15.5%	1.496, 7.6%	1.230, 8.6%	0.918, 8.0% *
CP, CP mean, CP(Q2)	ARS, 2.177, 2.05 *	ARSS, 1.445, 1.391 *	EM-N, 1.155, 1.133 *	knn-EuD, 0.879, 0.85
Ethanol (int. t. set), AR, AR/CP	2.034, 17.1%	1.361, 11.7%	1.040, 11.1%	0.732, 0%
CP, CP mean, CP(Q2)	EM-N, 1.832, 1.737 *	ARS, 1.318, 1.228 **	EM-N, 1.014, 0.936	AR, 0.732, 0.732
Acetone int. test set, AR, AR/CP	1.801, 3.7%	1.344, 11.4%	1.047, 8.8%	0.741, 8.7%
CP, CP mean, CP(Q2)	ARSS, 1.797, 1.737	EM-N, 1.240, 1.206 *	EM-N, 1.001, 0.962	EM-N, 0.701, 0.682 **
Methanol int. test set, AR, AR/CP	2.392, 26.8%	1.351, 11.1%	1.194, 15.0%	0.810, 1.1%
CP, CP mean, CP(Q2)	EM-N, 1.938, 1.886 *	EM-N, 1.232, 1.216 *	ARS, 1.041, 1.038 *	ARS, 0.802, 0.801
Log*D:* AR, AR/CP ^(a)^ CP, CP(Q2) [23]:	3.841, 32.8% EM-N, 2.892	2.237, 35.7% EM-N, 1.649	1.245, 3.8% EM-N, 1.200	0.843, 6.8% EM-N, 0.789

* Significantly narrower half-widths of normalized CP against AR, *t*-test, *p* < 0.01. ** *p* < 0.05. ^(a)^ According to Ref. [23], these are interval widths, not half-widths, but that does not affect the efficiency gain calculation. More details in Table S2.

**Table 8:** Estimate of total extrapolation accuracy obtained on three databases using two models, FSTI-XGB-ARSS for AqSolDB-n and EM-N for AqSolDB-w. The first four rows display AqSolDB-n statistics, and the second four rows show statistics of compounds that are within the AD of AqSolDB-w but not within the AD of AqSolDB-n. See Info Section S4 *.

Data Set	Q2(MW)	Total Size	In AD, Assessed	80% Q2(h-w)/Mean	LogS ± 1.0 > 80%	Est. LogS ± 1.0 *
AqSolDB-n	186.6	1619	900 (ext. test set)	0.738/0.751	93.3%	85.3% (true)
DrugBank	338.2	11,370	10,413	0.888/0.906	73.6%	82.8%
PubChem	323.4	72,739	68,535	0.885/0.912	75.4%	82.7%
COCONUT	390.5	406,919	356,277	0.918/0.946	66.9%	81.7%
Data set	Q2(MW)	In AD	Assessed (MW)	80% Q2(h-w)/mean	LogS ± 1.0 > 80%	Est. LogS ± 1.0 *
AqSolDB-w	232.2	9709	1611 (int. test)	0.860/0.971	65.51%	80.0% (true)
DrugBank	340.4	11,130	717 (585.9)	1.478/1.493	22.6%	63.3%
PubChem	329.8	71,808	3273 (689.0)	1.779/1.790	1.25%	55.3%
COCONUT	406.4	390,338	34,061 (863.3)	1.491/1.546	2.86%	61.7%
				Drugbank	PubChem	COCONUT
Final estimated LogS ±1.0 (Aq-n + Aq-w): *				81.6%	81.5%	79.9%

## Data Availability

All data presented in this study are openly available in the GitHub repository https://github.com/ojovic985/XGB-solubility with Supplementary_data_sets_table.xlsx containing all the input information (with SMILES) and final test results for all six used data sets, additional info files on GitHub (e.g., for QMvars of data sets (1, 4–6)), and SMILES for all three studied databases: DrugBank_11370.smi, PubChem_72739.smi, COCONUT_406919.smi. The software is and can be programmed in R using the xgboost library by following all instructions in Section 3. The authors supply the important codes in GitHub, although they are not mandatory to reproduce results, but for a better understanding (EM-N_code.R) and to reproduce the most important ones (Test_repr_code.R using FSTI-XGB_1619 model).

## Supplementary Materials

The following supporting information can be downloaded at: https://www.mdpi.com/article/10.3390/molecules29010019/s1, Main Supplementary Documentation File: Supplementary_documentation.pdf—containing Figures S1–S5, Tables S1 and S2 and Supporting Info Sections S1–S9. All the tables and figures are labelled in the supported PDF document. All LogS predictions of public databases are presented in Supplementary_databases_predictions.xlsx in Supplementary Materials.
