# Supplementary material for: Extreme Gradient Boosting Combined with Conformal Predictors for Informative Solubility Estimation
Source: Molecules. 2023 Dec 19;29(1):19. doi: 10.3390/molecules29010019 (PMC10779886; doi:10.3390/molecules29010019)
Supplement: Supplementary file 1 [file molecules-29-00019-s001.zip › Supplementary_documentation.pdf]

Supplementary documentation

Extreme Gradient Boosting combined with Conformal Predictors for Informative Solubility Estimation of Public Databases

Ozren Jovic, Rabah Mouras*

Pharmaceutical Manufacturing Technology Centre Department, Bernal Institute, Department of Chemical Sciences, University of Limerick, Limerick, Republic of Ireland

E-mails: ozren.jovic@ul.ie , rabah.mouras@ul.ie
***Corresponding author, phone number: +353892203838**

Table of contents

Table of contents..2

Supporting Info Tables S1a-b. Comparison between XGB and RF methodologies on smaller data sets (1, 4-6)......3

Supplementary figures S1-S3. Prediction performance of XGB and FSTI-XGB...4

Supporting Info section S1: Selected molecular descriptors using FSTI-XGB for data sets (1, 4-6).........................5

Supporting Info Section S2: Detailed comments on Ref. [25 in main article] that studied curated AqSolDB........11

Supporting Info Section S3: Tables S2a-p: Conformal predictor results with FSTI-XGB and XGB models.........13

Info section S4: CP estimation of the LogS±1 Accuracy on Databases void of experimental LogS..........................30

Supplementary figures S4-S5. Prediction intervals vs. Molecular weights (S4) and FSTI-XGB vs AlogPS............34

Info section S5: Python code for obtaining Padel and rdkit molecular descriptors from smiles.............................35

Info section S6: Calculation of ORCA SMD solvation Gibbs free energy..37

Info section S7: Names and meaning of used QMvars for Methanol data set..38

Info section S8: Names of QMvars of Water-wide, Ethanol and Acetone data sets...39

Info section S9: Calculation of NC measure for any number of calibration samples of certain confidence levels.40

Supporting Info Table S1a. The comparison between XGB and RF methodologies on the remaining four smaller data sets using all disposable variables that passed preprocessing. "P+QM" presents Padel and QM variables.

Dataset, vars type, (number of vars)	set size	RF fine-tuned					XGB				
		RMSE			%LogS±1.0		RMSE			%LogS±1.0	
		CV-set	V-set	RMSEtot	CV-set	V-set	CV-set	V-set	RMSEtot	CV-set	V-set
Water, P+QM (1156)	900	0.8826	0.8077	0.8493	79.4	84.0	0.8566	0.8257	0.8429	83.2	82.2
Ethanol, P+QM(1224)	695	0.7636	0.7863	0.7681	82.6	82.7	0.7135	0.7815	0.7437	89.7	82.0
Acetone, P+QM (1019)	452	0.7331	0.6662	0.7196	84.8	89.0	0.6901	0.7161	0.6953	88.9	83.5
Methanol, P+QM (1108)	135	0.9534	0.7516	0.8896	71.7	83.3	0.9197	0.7735	0.8807	68.7	86.1

Supporting Info Table S1b. Continued to Table S1a.

Dataset, vars type, (number of vars)	total set size	CV-training set size	V-test set size	%LogS±07			
				RF fine-tuned		XGB	
				CV-set	V-set	CV-set	V-set
Water, P+QM (1156)	900	500	400	66.2	69.5	66.0	70.5
Ethanol, P+QM (1224)	695	556	139	66.7	68.3	71.8	67.6
Acetone, P+QM (1019)	452	361	91	69.3	69.2	72.6	69.2
Methanol, P+QM (1108)	135	99	36	56.5	63.9	52.5	63.9

Comments related to Table S1:

(a) The data sets are of smaller set sizes than AqSolDB sets and carry less statistical weight. So in these tables, no statistically significant difference could be established between RF and XGB.

(b) RMSEtot is in very slight favor of XGB, but the overall results are not entirely in favor of XGB, although CV-statistics is mostly advantageous for XGB as CV-sets contain more samples than V-sets, and RF is in no slight advantage when taking into account all validation set statistics.

(c) The task of the paper is to select the better-performing methodology and to use it in modeling the large AquaSolDB data sets. So the higher accent is in comparison of model performances on large AquaSolDB data sets (2-3). That is why, these results were not put into the main article. Too many tables in the Results and Discussion section could lead to confusion. But, these tables can serve as supporting information tables.

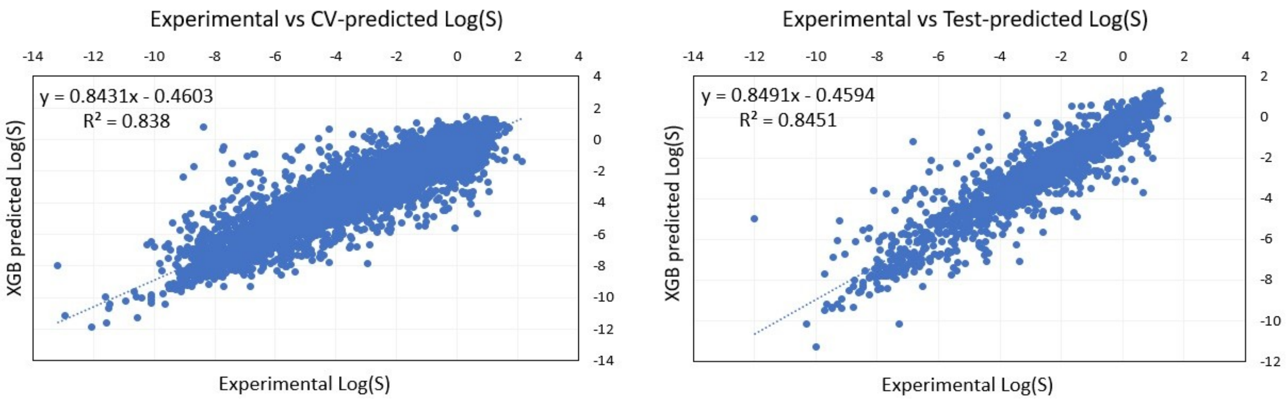

Supplementary Figure S1. Prediction performance of the XGB model (RMSECV = 0.9429, RMSEV = 0.9243) on the data set (2), with a number of descriptors = 1146. max_depth = 5, eta = 0.1, nrounds= 465. 95%, non-normalized half-width = 1.9658.

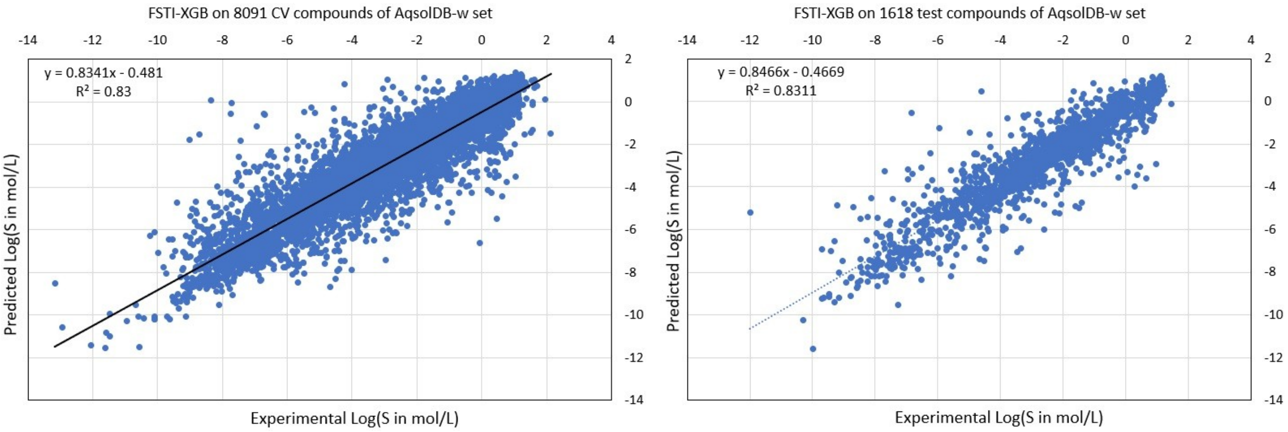

Figure S2. Prediction performance of FSTI-XGB model on AqSolDB-w set (3), with a number of molecular descriptors = 32.

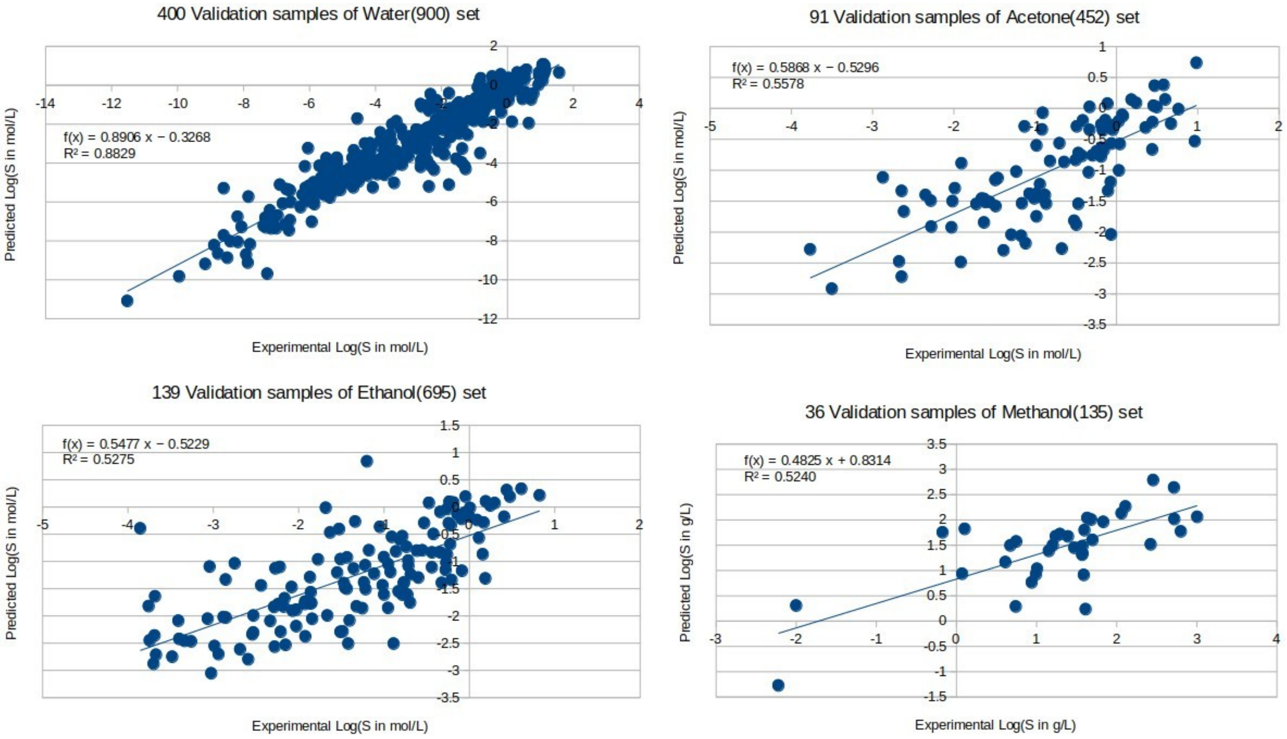

Figure S3. Experimental Log(S) vs ML predicted Log(S) of the final FSTI-XGB models whose performance is shown in Table 5. The top-left figure depicts statistics for 400 test validation samples of the Water data set. The top-right figure depicts statistics for 91 test validation samples. The bottom-left figure depicts statistics for 139 test validation samples from the Ethanol data set. The bottom-right figure displays statistics for 36 test validation samples of the Methanol data set.

Info section S1: Selected molecular descriptors using FSTI-XGB for data sets (1, 4-6)

All selected variables for Methanol (data set (1)) sorted by variable's top-down importance are: XLogP, ΔHfus..kJ.mol.1., MLFER_E, AATS8p, MATS3e, deltaG.aver, AATSC5m, nAtomP, GATS6i, ATSC6i, GATS3p, nAtomLAC, VR1_Dzs, MLFER_L, GATS4s, ATSC2e, GATS4e, ATSC4i, ZMIC2, AATSC5s, AATS1p, MATS5e, ETA_Shape_Y, R_TpiPCTPC, minsCH3, SIC0, minaasC, JGI6, Kier3, AATS3e, SpMax3_Bhm, MATS1m, AMR, VC.5.

Importance metric (from XLogP (Gain=1.403162e-01) to VC.5 (Gain=9.036388e-05)):

```
> xgb.importance(colnames(Fe2), model = model_xgboost)
F2[1,as.numeric(c(xgb.importance(colnames(Fe2), model = model_xgboost)[,1])$Feature)]
    Feature         Gain       Cover   Frequency
 1:       1 1.403162e-01 0.085336443 0.110332750
 2:       2 8.953146e-02 0.041475631 0.071803853
 3:       3 7.595728e-02 0.026979037 0.031523643
 4:       5 6.519174e-02 0.008480640 0.017513135
 5:      22 5.203537e-02 0.035168155 0.028021016
 6:      16 4.500070e-02 0.017305807 0.017513135
 7:      17 4.380952e-02 0.073384040 0.064798599
 8:      34 3.717002e-02 0.037102801 0.029772329
 9:      13 3.626000e-02 0.034214083 0.028021016
10:       4 3.585096e-02 0.024196327 0.028021016
11:       9 3.384537e-02 0.010812816 0.015761821
12:      11 2.981285e-02 0.017279305 0.015761821
13:      33 2.597081e-02 0.031908409 0.029772329
14:      12 2.228739e-02 0.008321628 0.010507881
15:      27 2.159036e-02 0.029920759 0.028021016
16:       7 2.144631e-02 0.049399730 0.036777583
17:      10 2.089681e-02 0.040521559 0.038528897
18:      31 2.066779e-02 0.044496860 0.035026270
19:      19 1.980454e-02 0.012084912 0.014010508
20:      29 1.900009e-02 0.036625765 0.035026270
21:      20 1.834659e-02 0.019584979 0.015761821
22:       6 1.728677e-02 0.003630774 0.010507881
23:      23 1.718512e-02 0.083799327 0.064798599
24:      26 1.716239e-02 0.015768691 0.021015762
25:      24 1.174938e-02 0.030636313 0.031523643
26:      28 1.150608e-02 0.027058543 0.028021016
27:      30 1.034209e-02 0.016113217 0.012259194
28:       8 1.013454e-02 0.020910079 0.019264448
29:      25 8.535835e-03 0.025282909 0.021015762
30:      21 8.158879e-03 0.032650465 0.024518389
31:      15 7.298207e-03 0.047226566 0.040280210
32:      18 4.966952e-03 0.005962950 0.017513135
33:      14 7.912693e-04 0.001457610 0.003502627
34:      32 9.036388e-05 0.004902870 0.003502627
```

All selected variables for AqSolDB-w (data set (2)) sorted by variable's top-down importance are: MolLogP, XLogP, TpiPC, BalabanJ, BertzCT, AATS1i, MolWt, GATS1s, AATSC2e, piPC1, GATS2c, TPSA, piPC3, ZMIC1, Mv, MATS1e, MolMR, AATS7p, AATS3v, AATS6v, GATS1m, MWC3, TPC, MDEO.11, MDEC.33, nAtomP, AATS1v, AATS4v, AATS4m, AATS7v, AATS0v, nAcid.

Importance metric (from MolLogP (Gain=0.649127447) to nAcid (Gain=0.003849456)):

```
F2[1,as.numeric(c(xgb.importance(colnames(Fe2), model = model_xgboost)[,1])$Feature)]
#poredane varijable po važnosti u xgb modelu (gain-u)
```

	Feature	Gain	Cover	Frequency
1:	1	0.649127447	0.094215140	0.131493692
2:	2	0.043994918	0.059402130	0.058162137
3:	3	0.029777947	0.026362912	0.041925525
4:	5	0.017022725	0.043801608	0.049662690
5:	4	0.015159129	0.033720450	0.036398979
6:	11	0.014668722	0.059078591	0.041125129
7:	7	0.014226303	0.029095441	0.040172276
8:	10	0.013521849	0.051966279	0.046651675
9:	21	0.013183448	0.045232768	0.041048900
10:	6	0.012572506	0.014527762	0.008918703
11:	29	0.012407293	0.027277208	0.041849297
12:	14	0.012243212	0.042115428	0.034531387
13:	13	0.011358522	0.013187537	0.012958799
14:	8	0.011234262	0.041757134	0.038990738
15:	9	0.010915963	0.014014717	0.024126234
16:	26	0.010547425	0.044385082	0.037885429
17:	23	0.008575546	0.035416883	0.031215459
18:	25	0.008389927	0.021781108	0.020086138
19:	16	0.008185082	0.030303684	0.028585585
20:	19	0.007987183	0.022106026	0.025231543
21:	30	0.007739308	0.043629886	0.027175363
22:	17	0.007435576	0.016949940	0.013492396
23:	28	0.007414535	0.016261708	0.013682967
24:	22	0.007179346	0.019670979	0.017456264
25:	15	0.006929127	0.026282584	0.025917597
26:	20	0.006659118	0.015445488	0.014140336
27:	24	0.005981572	0.020555222	0.017189465
28:	18	0.005619250	0.021833823	0.024393033
29:	32	0.005584264	0.020525135	0.020695964
30:	31	0.005262395	0.016570327	0.016160384
31:	27	0.005246645	0.023469373	0.012539543
32:	12	0.003849456	0.009057648	0.006136372

All selected variables for AqSolDB-n (data set (3)) sorted by variable's top-down importance are: MolLogP, ATS0p, XLogP, ZMIC1, GATS2c, piPC2, MPC7, AATS1i, MDEC-33, piPC3, AATS6v, TpiPC, nH, AATS5p, AATS1e, ATS1m, ZMIC2, TWC, piPC6, MPC8, AATS4v, MolMR, piPC1, piPC10, Mi, piPC4

Importance metric (from MolLogP (Gain=0.730166750) to piPC4 (Gain=0.002875328)):

```
F2[1,as.numeric(c(xgb.importance(colnames(Fe2), model = model_xgboost)[,1])$Feature)]
#poredane varijable po važnosti u xgb modelu (gain-u)
```

	Feature	Gain	Cover	Frequency
1:	1	0.730166750	0.071531492	0.127491166
2:	3	0.032449507	0.040030807	0.053851590
3:	2	0.032043385	0.090402646	0.075901060
4:	4	0.029045112	0.037983252	0.045371025
5:	19	0.015952347	0.078169430	0.086643110
6:	5	0.011825711	0.017753437	0.029964664
7:	7	0.011618177	0.017339910	0.023038869
8:	17	0.010922525	0.055408736	0.049469965
9:	6	0.010195707	0.038797398	0.036325088
10:	9	0.010026923	0.020064883	0.021342756
11:	15	0.009369433	0.036523719	0.040282686
12:	14	0.009262002	0.034577514	0.019505300
13:	23	0.008424132	0.026007714	0.023180212
14:	12	0.008359439	0.060476230	0.050318021
15:	20	0.008145935	0.069419399	0.050883392
16:	24	0.007414872	0.041138006	0.034911661
17:	10	0.007378082	0.068505338	0.048904594
18:	22	0.006903413	0.019179506	0.019505300
19:	11	0.006812002	0.025357545	0.024028269
20:	26	0.005685699	0.008783018	0.012579505
21:	21	0.005358526	0.030618177	0.036466431
22:	13	0.005356312	0.029404847	0.023886926
23:	8	0.005262050	0.016485129	0.011307420
24:	18	0.004808029	0.013344718	0.009187279
25:	25	0.004338605	0.037641436	0.031378092
26:	16	0.002875328	0.015055714	0.014275618

All selected variables for Water (data set (4)) sorted by variable's top-down importance are: MolLogP, XLogP, CrippenLogP, CrippenMR, SpMax1_Bhm, GGI5, ETA_Eta_F, MP (QM), AATS6v, MDEC.33, SpMax2_Bhm, ALogp2, SpMax3_Bhm, Mi, ETA_EtaP_F, ZMIC5, LipoaffinityIndex, GATS7v, MIC1, ATSC1m, AATS1i, minHBa, MDEO.12, AATSC2s, AATS2i, ETA_Beta_s, GGI9, piPC10, HOMO (QM), ATS6v, SsNH2, ZMIC2, piPC9.

Importance metric (from MolLogP (Gain=0.5957405562) to piPC9 (Gain=0.0008917595)):

```
> xgb.importance(colnames(Fe2), model = model_xgboost)
    Feature         Gain       Cover Frequency
 1:       1 0.5957405562 0.041492850    0.0808
 2:       2 0.0797850938 0.118110835    0.0832
 3:       6 0.0368367821 0.027798840    0.0312
 4:       3 0.0358869542 0.012664423    0.0292
 5:       5 0.0309868099 0.014254450    0.0192
 6:       4 0.0298700294 0.014731204    0.0292
 7:       9 0.0217689134 0.010273043    0.0112
 8:      13 0.0143036842 0.066380445    0.0692
 9:      11 0.0132755837 0.015058339    0.0232
10:      14 0.0119804803 0.021073560    0.0224
11:      16 0.0109634784 0.027200361    0.0268
12:      17 0.0108251695 0.045788712    0.0484
13:       8 0.0086144576 0.010724438    0.0128
14:      10 0.0084249320 0.041031311    0.0272
15:      21 0.0082337719 0.038632323    0.0312
16:      24 0.0068299606 0.042808996    0.0308
17:      12 0.0066918660 0.047566397    0.0404
18:      26 0.0064013159 0.011193585    0.0176
19:      23 0.0057683168 0.018091309    0.0256
20:      22 0.0057030912 0.025970436    0.0380
21:      19 0.0056855997 0.018727826    0.0136
22:      29 0.0055507291 0.041906206    0.0384
23:      32 0.0054619625 0.024953529    0.0200
24:      33 0.0051644279 0.069699974    0.0456
25:      25 0.0045017347 0.034866471    0.0332
26:      20 0.0041492324 0.007577352    0.0096
27:      18 0.0038544550 0.024664433    0.0176
28:      15 0.0035618200 0.006486903    0.0080
29:      28 0.0035105811 0.063897772    0.0512
30:      31 0.0033424438 0.024882523    0.0236
31:      27 0.0031170037 0.014759100    0.0104
32:       7 0.0023170034 0.014355887    0.0260
33:      30 0.0008917595 0.002376164    0.0052
```

In the Ethanol model (data set (5)) (P+QMvars) sorted top-down descriptors (in importance) are: MP (QM), SpMAD_Dt, SsOH, ALogp2, SpAbs_Dzp, XLogP, SN1_Dzi, BertzCT, Most_neg (QM), GATS4e, SpMax_Dt, MLFER_L, SpMin6_Bhp. SpMAD_Dzp, AATSC3v, AATSC4i, nHsNH2, LUMO (QM), SpMax3_Bhm, maxHBd, GATS2c, minHBint5, IC2, AATS7m, SpMax2_Bhe, SpMax5_Bhe_ nBondsS3, GATS7cm SpMax4_Bhm, WTPT.3, MATS1e, maxsOH, SpMax_D.

Importance metric (from MP (Gain=0.195185733) to SpMax_D (Gain=0.001676379)):

```
> xgb.importance(colnames(Fe2), model = model_xgboost)
    Feature        Gain       Cover   Frequency
 1:       1 0.195185733 0.082503750 0.100326264
 2:       2 0.077769877 0.021516130 0.032626427
 3:       3 0.063826109 0.029010410 0.037520392
 4:       6 0.043027343 0.058696607 0.069331158
 5:       7 0.038916382 0.007803385 0.017128874
 6:       9 0.034173458 0.028819492 0.024469821
 7:      11 0.033669501 0.042620119 0.026916803
 8:       4 0.033306260 0.016009819 0.026916803
 9:      10 0.032266435 0.041165508 0.039967374
10:      18 0.032096345 0.041192781 0.044861338
11:       5 0.024625277 0.020119096 0.020391517
12:      12 0.023587937 0.032422686 0.023654160
13:      22 0.023577061 0.023685925 0.021207178
14:      17 0.023559088 0.015303726 0.019575856
15:      27 0.023085893 0.073248784 0.054649266
16:      15 0.023084568 0.039101777 0.038336052
17:       8 0.022555972 0.010679273 0.008972268
18:      21 0.022022329 0.030522600 0.035889070
19:      33 0.021597200 0.029410428 0.030995106
20:      29 0.020454306 0.028646757 0.026916803
21:      24 0.020111613 0.024852645 0.030995106
22:      14 0.019454627 0.032486325 0.025285481
23:      26 0.018518149 0.039344213 0.035889070
24:      30 0.018384903 0.044632332 0.032626427
25:      28 0.018171263 0.033234844 0.026101142
26:      20 0.016409926 0.012236920 0.014681892
27:      19 0.015389018 0.008330682 0.015497553
28:      25 0.014826257 0.018009910 0.019575856
29:      23 0.014073632 0.033728806 0.026101142
30:      16 0.011040851 0.017658378 0.018760196
31:      31 0.010882760 0.026089065 0.023654160
32:      32 0.008673547 0.024664757 0.019575856
33:      13 0.001676379 0.012252072 0.010603589
```

All selected variables for Acetone (data set (6)) sorted by variable's top-down importance are: MP (QM), MLFER_L, MLFER_E, GATS1v, AATSC2e, MATS3v, MDEN.22, MLFER_BH, AATSC8p, GATS1p, ETA_Eta_L, MATS1p, TIC2, CIC1, GATS1m, ATSC2m, AATS7i, GATS4s, BCUTw.1l, MATS3p, ATS2m, Asp1 (QM), topoDiameter, nHBAcc, SHBint7, MLFER_S, MATS2m, ATSC5m, GATS7v, Area3 (QM), SpMAD_Dt.

Importance metric (from MP (Gain=0.252155261) to SpMAD_Dt (Gain=0.002075200)):

	Feature	Gain	Cover	Frequency
1:	1	0.252155261	0.153537606	0.174142480
2:	2	0.073710469	0.021201671	0.029023747
3:	3	0.070871613	0.014679683	0.010554090
4:	4	0.053659427	0.009355823	0.029023747
5:	7	0.040549505	0.062063074	0.063324538
6:	13	0.039640065	0.045716429	0.036939314
7:	6	0.035333931	0.020805768	0.023746702
8:	5	0.034939810	0.023900066	0.018469657
9:	17	0.033812719	0.089088693	0.068601583
10:	9	0.030806800	0.060739923	0.047493404
11:	12	0.028912694	0.037381620	0.036939314
12:	20	0.026480536	0.028619651	0.031662269
13:	8	0.024659070	0.009574612	0.010554090
14:	15	0.024467803	0.052790598	0.044854881
15:	19	0.023159066	0.014002480	0.013192612
16:	18	0.020957193	0.020045216	0.026385224
17:	21	0.019359079	0.033589281	0.042216359
18:	29	0.018382835	0.044747507	0.055408971
19:	31	0.017508261	0.039757040	0.044854881
20:	11	0.016760833	0.016596689	0.015831135
21:	16	0.016717786	0.018399092	0.015831135
22:	26	0.016445067	0.039767459	0.034300792
23:	22	0.016231313	0.013627413	0.010554090
24:	10	0.013237422	0.004573727	0.005277045
25:	28	0.010916826	0.011564548	0.010554090
26:	25	0.009674339	0.024639780	0.023746702
27:	23	0.009090862	0.015044331	0.010554090
28:	30	0.008104322	0.023618766	0.018469657
29:	24	0.006958091	0.007657606	0.013192612
30:	14	0.004421801	0.035422939	0.026385224
31:	27	0.002075200	0.007490910	0.007915567

Info Section S2: Detailed comments on Ref. [25 in main article] that studied curated AqSolDB

Ref. [25 in the main article] reports the most accurate model with RMSE=0.72 of the test set (335 in size) and RMSEV=0.76 of the validation set (335) among four final models (two linear and two non-linear) obtained using artificial neural networks for non-linear models. But, careful reading of the reference [25] reveals the following differences between our approach and the discussed study:

(1) Ref. [25] used 1674 compounds out of 1818 possible, not one-fragment 1619 compounds of HAC>3 as we considered. The reason they state is "*incapability of calculations of some Dragon descriptors*", although their most accurate model, NN-A, wasn't built on any Dragon descriptors, only on some available rdkit AqSolDB descriptors. The question is why 144 compounds were not included when they could be included. Also, careful inspection of the smiles in their supplementary data "cca3776_Supplement.pdf" reveals ca. 20 compounds among 1674 compounds with HAC≤3, and smiles strings containing dots, meaning that not all compounds are one-fragment molecules. Also, our CV result is based on all 1399 training samples, while they used 335 of them as an optimizing test set.

(2) They separated the 1674 compounds into the training (1004), test (335, used for optimization), and final validation set (335). At the same time, they state in their paper that the models with the highest quality indicators (RMSE, R2, Q2F3, CCC) of the validation set were selected. However, the use of the external validation set's accuracy statistics for model selection makes that data set not a "final validation set", as its performance is then also used in model optimization as the other two data sets (training and test) were used in the optimization of the "best" model.
We cite these parts in their paper:

[QUOTE]
1. "*The most similar compounds that were located on the same neuron were then split in training, test and validation datasets.*"

2. *The methodology applied in this work relies on division of the data into training, test and validation set of compounds. Here, we have to take care that the compounds in the validation set, which serves for the final validation of the models, is separated from the rest of compounds at very beginning, prior to any data curation. Then the test set is selected as described, and it serves for internal intermediate testing during the model development and optimization. This standard procedure has proven to be the most robust against potential overfitting.*

3. *The criteria used for selection of the best regression models were several validation parameters, which are RMSE, R2, Q2F3, CCC.[47–49] The models with the highest quality indicators of the validation set were selected.*

4. During optimization process hundreds of models were generated, but only the best four models were selected and represented in this study (models NN-A, Q-A, NN-D, Q-D) for two sets of descriptors (A: AqSol 17 descriptors and D: Dragon Randić-like 94 descriptors) and two modelling

methods (NN for neural networks and Q for MLR). The QSARINS and CP-ANNatNIC software are well known and frequently used tools for linear and non-linear QSAR models.[43,45] The best models were chosen by using Root Mean Square Error for training set (RMSETR) and validation set (RMSEV) as the optimization value criteria.
[END of QUOTE]

Our comment: Given all the above at the same time, then there is no "final validation set" (i.e. no external test set), but three different data sets in optimization and selection of the model for each of the four methodologies. Besides, regarding point (1) above, that is making compound data sets mutually too similar, and since it didn't fall under AD rules, it should have been externally validated with an additional test set, but it wasn't. Otherwise, it is a question of how robust and applicable the models can be when used for extrapolation to external databases in which compounds do not fall within the same neuron.

(3) Their second-best model using the same artificial neural networks but on Dragon descriptors (instead of AquaSolDb descriptors) obtained RMSE of the test and validation set of 1.07 and 0.96, respectively. There is a prominent gap in the final result between their two best models (the top model has RMSEtot of 0.74 LogS, and the second 1.00 LogS) although they are both obtained with the same ANN method (just a slight difference in optimization criterion). Dragon descriptors are different variables, but well-known and explained [https://vcclab.org/lab/edragon/] [https://doi.org/10.3389/fchem.2022.852893]. This gap in performance between their two best models is hardly expected for a so large dataset, in our opinion, but the authors did not comment on it thoroughly. We obtained very similar RMSEtot between our XGB and FSTI-XGB on AquaSolDB data sets, although there is an extreme difference in the number of variables between XGB and FSTI-XGB. In addition, four different solubility models on 3664 water data sets also achieved a very close range of cross-validation RMSE statistics of 0.84-0.873 LogS [REF 41 in the main article]. We explained two objective reasons why AqSolDB-n obtained RMSEtot of 0.71 LogS and AqSolDB-w 0.97 LogS - they are two very different compound data sets. It is possible to obtain large differences in performance on the same data set with the same method, but different descriptors, although such a data set is either relatively small (our Methanol set had only 99 training and 36 test compounds (see Table 3)) or the used molecular descriptors for the less performing model should be weakly informative, which is unusual that it might be the case for 22 Dragon descriptors.

(4) The authors did not present %LogS±0.7 or %LogS±1.0 accuracy statistics in their results but put many correlation coefficient definitions in their article. We didn't have to use so many as our R^2(val) was already stronger than theirs, and as the accent of our paper is on %LogS±1.0, to compare results with Ref. [12] and later with results of CPs. If we use too many accuracy definitions in one subsection, that might potentially lead to confusion.

We did not want to put all these points and comments in the main article (especially not citations which cannot be all put like that in the main text).

Info Section S3: Supporting Info Tables S2a-n: Conformal predictor results with FSTI-XGB and XGB models

Table S2a. CP efficiency results, i.e. prediction interval half-widhts, on AqSolDB-w test set for FSTI-XGB model (32 vars. model)

Method		99%		95%		90%		80%		average of all	average of
	β	mean	median	mean	median	mean	median	mean	median	8 statistics	all means
AR	-	3.439	3.439	2.043	2.043	1.489	1.489	1.006	1.006	1.994	1.994
ARS	0	3.193	2.733	1.856	1.588	1.425	1.220	1.033	0.885	1.742	1.877
ARS	0.2	2.906	2.720	1.751	1.639	1.350	1.264	**0.959**	**0.898**	1.686	1.742
ARS	0.5xQ2(σ)	2.905	2.612	**1.717**	**1.544**	1.364	1.226	0.977	0.879	1.653	1.741
ARS	1xQ2(σ)	2.869	2.647	1.730	1.596	1.355	1.250	0.964	0.889	1.663	1.730
ARS	2xQ2(σ)	2.947	2.791	1.768	1.674	1.362	1.290	0.960	0.909	1.713	1.759
ARS	6xQ2(σ)	3.111	3.039	1.863	1.820	1.422	1.389	0.967	0.944	1.819	1.841
ARS	1.0	3.141	3.079	1.881	1.843	1.427	1.399	0.967	0.947	1.836	1.854
ARSS	0	3.182	2.723	1.886	1.614	1.463	1.252	1.051	0.899	1.759	1.896
ARSS	0.2	2.904	2.719	1.760	1.648	1.383	1.295	0.988	0.925	1.703	1.759
ARSS	1.0	3.125	3.063	1.918	1.880	1.441	1.413	1.003	0.983	1.853	1.872
ARSS	0.5xQ2(σ)	2.806	2.524	1.744	1.568	1.390	1.250	0.993	0.893	1.646	1.733
ARSS	1xQ2(σ)	2.882	2.658	1.745	1.610	1.386	1.278	0.987	0.911	1.682	1.750
ARSS	2xQ2(σ)	2.964	2.807	1.789	1.694	1.385	1.311	0.985	0.933	1.734	1.781
ARSS	6xQ2(σ)	3.122	3.049	1.895	1.851	1.438	1.404	0.996	0.972	1.841	1.863
EM-N	0	2.811	2.359	1.830	1.536	1.428	1.198	1.031	0.866	1.632	1.775
EM-N	0.2	2.662	2.332	1.743	1.527	1.356	1.188	0.981	0.859	1.581	1.686
EM-N	1.0	2.720	2.544	1.759	1.645	1.351	1.263	0.969	0.906	1.645	1.700
EM-N	0.5xQ2(σ)	**2.657**	**2.355**	1.735	1.537	1.350	1.196	0.971	0.860	1.583	1.678
EM-N	1xQ2(σ)	2.698	2.461	1.737	1.585	1.347	1.229	0.967	0.882	1.613	1.687
EM-N	2xQ2(σ)	2.732	2.567	1.769	1.662	1.358	1.276	0.969	0.910	1.655	1.707
EM-N	6xQ2(σ)	3.006	2.925	1.859	1.809	1.418	1.380	0.968	0.942	1.788	1.813
EM-N	***0.3xQ2(σ)***	*2.667*	*2.323*	*1.742*	*1.517*	*1.360*	*1.185*	*0.987*	*0.860*	*1.580*	1.689
EM-Log	0	2.983	2.561	1.846	1.584	1.441	1.237	1.050	0.901	1.700	1.830
EM-Log	0.2	2.745	2.478	1.759	1.587	1.345	1.214	0.982	0.886	1.625	1.708
EM-Log	1.0	2.845	2.722	1.798	1.720	1.382	1.323	0.978	0.936	1.713	1.751
EM-Log	0.5xQ2(σ)	2.734	2.461	1.761	1.585	1.350	1.215	0.984	0.886	1.622	1.707
EM-Log	1xQ2(σ)	2.728	2.517	1.753	1.617	**1.342**	**1.238**	0.974	0.899	1.634	1.699
EM-Log	2xQ2(σ)	2.832	2.682	1.783	1.688	1.365	1.293	0.977	0.925	1.693	1.739
EM-Log	6xQ2(σ)	2.981	2.911	1.872	1.828	1.421	1.388	0.978	0.955	1.792	1.813
knn-EuD	0	3.454	2.815	2.167	1.766	1.633	1.331	1.125	0.917	1.901	2.095
knn-EuD	1xQ2(σ)	3.123	2.805	1.968	1.768	1.489	1.768	1.022	0.918	1.858	1.901
knn-EuD	2xQ2(σ)	3.090	2.873	1.934	1.799	1.461	1.358	1.006	0.935	1.807	1.873
knn-EuD	6xQ2(σ)	3.134	3.036	1.963	1.902	1.473	1.427	0.996	0.965	1.862	1.892

*Q2(σ, ARS)=Q2(σ, ARSS)=0.1370
**Q2(σ, EM-N)=0.5586
***Q2(σ, EM-log)=0.3774
****Q2(σ, kNN-EuD)=1.8687

Table S2b. CP error rate results, i.e. misclassification rates, on AqSolDB-w test set for FSTI-XGB model (32 vars. model)

Error rates	99.00%	95.00%	90.00%	80.00%
AR	1.17%	4.70%	9.70%	20.02%
ARS b=0	0.62%	5.69%	10.32%	19.84%
ARS b=0.2	1.05%	4.82%	10.20%	20.27%
ARS b=0.5	1.24%	4.82%	9.52%	20.52%
ARS b=0.5xQ2(σ)	0.87%	5.50%	10.26%	19.47%
ARS b=1xQ2(σ)	0.99%	5.25%	10.20%	19.90%
ARS b=2xQ2(σ)	1.11%	5.01%	9.89%	20.64%
ARS b=6xQ2(σ)	1.17%	4.82%	9.58%	20.40%
ARS b=1.0	1.17%	4.82%	9.58%	20.77%
ARSS b=0	0.62%	5.39%	10.07%	19.34%
ARSS b=0.2	1.05%	4.76%	9.70%	19.22%
ARSS b=0.5	1.24%	4.70%	9.27%	19.34%
ARSS b=1	1.17%	4.51%	9.33%	19.04%
ARSS b=0.5xQ2(σ)	1.11%	5.32%	9.64%	19.16%
ARSS b=1xQ2(σ)	0.99%	5.19%	9.70%	19.04%
ARSS b=2xQ2(σ)	0.99%	5.19%	9.70%	19.04%
ARSS b=6xQ2(σ)	1.05%	4.64%	9.21%	19.34%
EM-N b=0	1.17%	5.32%	10.51%	20.02%
EM-N b=0.2	1.11%	5.07%	10.82%	20.70%
EM-N b=0.5	0.93%	5.01%	10.14%	19.96%
EM-N b=1.0	1.11%	5.19%	10.14%	20.02%
EM-N b=0.5xQ2(σ)	1.11%	4.82%	10.51%	20.21%
EM-N b=1xQ2(σ)	0.93%	5.13%	10.14%	19.96%
EM-N b=2xQ2(σ)	1.24%	5.13%	10.07%	19.96%
EM-N b=6xQ2(σ)	1.36%	5.01%	9.64%	20.70%
EM-N b=0.3xQ2(σ)	1.11%	5.13%	10.94%	20.64%
EM-LOG b=0	1.05%	5.25%	9.64%	19.65%
EM-LOG b=0.2	1.17%	4.94%	10.32%	20.21%
EM-LOG b=0.5	1.05%	5.01%	10.32%	20.33%
EM-LOG b=1	1.17%	5.01%	10.26%	20.27%
EM-LOG b=0.5xQ2(σ)	1.17%	4.94%	10.32%	20.21%
EM-LOG b=1xQ2(σ)	1.05%	5.07%	10.51%	20.09%
EM-LOG b=2xQ2(σ)	1.17%	5.13%	10.44%	20.15%
EM-LOG b=6xQ2(σ)	1.30%	4.88%	9.77%	20.70%
kNN-EuD b=0	0.93%	4.82%	9.64%	20.46%
kNN-EuD b=1 x Q2(σ)	0.99%	4.76%	9.33%	20.46%
kNN-EuD b=2 x Q2(σ)	0.99%	4.88%	9.46%	20.40%
kNN-EuD b=6 x Q2(σ)	1.36%	4.45%	9.39%	20.33%

*Q2(σ, ARS)=Q2(σ, ARSS)=0.1370
**Q2(σ, EM-N)=0.5586
***Q2(σ, EM-log)=0.3774
****Q2(σ, kNN-EuD)=1.8687

Table S2c. CP efficiency results, i.e. prediction interval half-widhts, on AqSolDB-w test set (1618) for XGB model (1146 vars. model)

Method		99%		95%		90%		80%		average of all	average of
	β	mean	median	mean	median	mean	median	mean	median	8 statistics	all means
AR	-	3.407	3.407	1.964	1.964	1.465	1.465	0.974	0.974	1.953	1.953
ARSS	0	2.969	2.613	1.813	1.596	1.417	1.247	1.022	0.899	1.697	1.805
ARSS	0.2	2.806	2.641	1.752	1.649	1.350	1.271	0.966	0.909	1.668	1.719
ARSS	0.5	2.922	2.824	1.801	1.741	1.388	1.341	0.957	0.925	1.737	1.767
ARSS	1	3.050	2.991	1.845	1.809	1.409	1.381	0.966	0.947	1.800	1.818
EM-N	0	2.759	2.318	1.748	1.469	1.362	1.144	0.994	0.835	1.579	1.716
EM-N	***0.2***	*2.591*	*2.273*	*1.648*	*1.445*	*1.299*	*1.139*	*0.948*	*0.831*	*1.522*	1.622
EM-N	0.5	2.584	2.348	1.643	1.493	1.288	1.171	0.926	0.842	1.537	1.610
EM-N	1.0	2.645	2.476	1.673	1.567	1.304	1.221	0.924	0.865	1.584	1.637
EM-N	0.3xQ2(σ)	2.601	2.268	1.665	1.451	1.305	1.138	0.953	0.830	1.526	1.631
EM-N	0.5xQ2(σ)	2.610	2.314	1.633	1.448	1.291	1.145	0.939	0.833	1.527	1.618
EM-N	1.0xQ2(σ)	**2.595**	**2.367**	**1.644**	**1.500**	**1.284**	**1.171**	**0.928**	**0.846**	1.542	1.613
EM-EXP	0	2.985	2.245	1.873	1.408	1.456	1.095	1.053	0.792	1.613	1.842
EM-EXP	0.2	2.962	2.286	1.842	1.422	1.430	1.103	1.034	0.798	1.610	1.817
EM-EXP	0.5	2.879	2.291	1.811	1.441	1.411	1.123	1.018	0.810	1.598	1.780
EM-EXP	1.0	2.816	2.327	1.792	1.481	1.395	1.153	0.997	0.824	1.598	1.750

Q2(σ, ARSS)=0.1662
Q2(σ, EM-N)=0.5461
Q2(σ, EM-EXP)=1.7264

Table S2d. CP error rate results, i.e. misclassification rates, on AqSolDB-w test set (1618) for XGB model (1146 vars. model)

Error rates	99.00%	95.00%	90.00%	80.00%
AR	0.80%	5.13%	9.70%	19.16%
ARSS b=0	0.93%	5.31%	9.33%	18.29%
ARSS b=0.2	0.74%	5.32%	9.64%	19.10%
ARSS b=0.5	0.80%	5.32%	9.64%	19.16%
ARSS b=1	0.80%	5.19%	9.77%	18.79%
EM-N b=0	1.17%	6.37%*	10.69%	20.02%
EM-N b=0.2 (0.366xQ2(σ))	0.87%	6.24%	11.06%	19.96%
EM-N b=0.5	0.74%	6.18%	10.82%	19.84%
EM-N b=1.0	0.87%	6.12%	10.38%	19.90%
EM-N b=0.3*Q2(σ)**	0.99%	6.30%	11.19	20.21%
EM-N b=0.5*Q2(σ)	0.80%	6.30%	10.94%	19.96%
EM-N b=1*Q2(σ)	0.74%	6.30%	11.00%	19.72%
EM-EXP-Lev b=0***	0.80%	5.81%	10.82%	19.96%
EM-EXP-Lev b=0.2	0.93%	5.87%	11.00%	19.96%
EM-EXP-Lev b=0.5	1.05%	6.00%	11.06%	20.21%
EM-EXP-Lev b=1.0	1.05%	5.93%	10.57%	20.27%

Q2(σ, ARSS)=0.1662
Q2(σ, EM-N)=0.5461
Q2(σ, EM-EXP)=1.7264

Table S2e. CP efficiency results, i.e. prediction interval half-widhts, on AqSolDB-n internal test set (220), FSTI-XGB model

Method		99%		95%		90%		80%		average of all	average of
	β	mean	median	mean	median	mean	median	mean	median	8 statistics	all means
AR	-	2.335	2.335	1.534	1.534	1.176	1.176	0.783	0.783	1.457	1.457
ARSS	0	2.197	2.031	1.386	1.281	1.070	0.989	0.803	0.742	1.312	1.364
ARSS	0.2	2.018	1.955	1.328	1.286	1.069	1.035	0.754	0.731	1.272	1.292
ARSS	0.5	2.077	2.041	1.392	1.368	1.085	1.066	0.766	0.753	1.319	1.330
ARSS	0.3xQ2(σ)	1.998	1.881	1.329	1.251	1.059	0.996	0.786	0.740	1.255	1.293
ARSS	0.5xQ2(σ)	1.960	1.859	1.322	1.254	1.038	0.985	0.777	0.737	1.242	1.274
ARSS	1xQ2(σ)	1.979	1.902	1.321	1.270	1.042	1.002	0.759	0.729	1.251	1.275
ARSS	1.5xQ2(σ)	2.017	1.954	1.329	1.288	1.070	1.037	**0.755**	**0.731**	1.273	1.293
ARSS	2xQ2(σ)	1.994	1.942	1.346	1.311	1.088	1.060	0.757	0.737	1.279	1.296
ARSS	3xQ2(σ)	2.034	1.994	1.381	1.354	1.092	1.071	0.765	0.750	1.305	1.318
ARSS	1.0	2.12	2.100	1.440	1.427	1.109	1.098	0.772	0.765	1.354	1.360
ARS	0	2.200	2.034	1.383	1.280	1.079	0.997	0.809	0.748	1.316	1.368
ARS	0.2	2.023	1.959	1.305	1.264	1.040	1.007	0.764	0.740	1.263	1.283
ARS	0.5	2.108	2.072	1.368	1.345	1.073	1.055	0.780	0.767	1.321	1.332
ARS	0.3xQ2(σ)	2.011	1.892	1.333	1.254	1.061	0.999	0.774	0.728	1.257	1.295
ARS	0.5xQ2(σ)	1.952	1.852	1.287	1.221	1.043	0.989	0.771	0.731	1.231	1.263
ARS	1xQ2(σ)	2.035	1.956	1.293	1.242	1.035	0.995	0.761	0.732	1.256	1.281
ARS	1.5xQ2(σ)	2.022	1.959	1.305	1.264	1.038	1.006	0.765	0.741	1.263	1.283
ARS	2xQ2(σ)	1.999	1.947	1.342	1.306	1.048	1.020	0.774	0.754	1.274	1.291
ARS	3xQ2(σ)	2.053	2.012	1.356	1.329	1.069	1.047	0.776	0.761	1.300	1.314
ARS	1.0	2.166	2.146	1.414	1.401	1.097	1.086	0.776	0.768	1.357	1.363
EM-N	0	2.185	2.016	1.352	1.248	1.095	1.011	0.825	0.761	1.312	1.364
EM-N	0.2	1.939	1.832	1.284	1.213	1.050	0.992	0.772	0.729	1.226	1.261
EM-N	0.5	1.840	1.768	1.296	1.245	1.039	0.999	*0.755*	*0.726*	1.209	1.233
EM-N	1.0	1.930	1.880	1.345	1.310	1.059	1.031	0.762	0.742	1.257	1.274
EM-N	0.5xQ2(σ)	1.905	1.804	***1.267***	***1.199***	1.047	0.992	0.763	0.723	1.213	1.246
EM-N	***1xQ2(σ)***	***1.831***	***1.757***	*1.285*	*1.233*	***1.034***	***0.992***	*0.758*	*0.727*	*1.202*	1.227
EM-N	1.5xQ2(σ)	1.883	1.822	1.318	1.275	1.042	1.008	0.762	0.737	1.231	1.251
EM-N	2xQ2(σ)	1.924	1.872	1.336	1.299	1.054	1.025	0.765	0.744	1.252	1.270
EM-N	3xQ2(σ)	1.976	1.935	1.376	1.347	1.046	1.025	0.761	0.745	1.276	1.290
EM-N	6xQ2(σ)	2.036	2.012	1.426	1.409	1.084	1.071	0.777	0.768	1.323	1.331
EM-N	0.3xQ2(σ)	1.954	1.836	1.303	1.225	1.047	0.984	0.782	0.735	1.233	1.272
EM-Log	0	2.302	2.065	1.441	1.292	1.171	1.050	0.859	0.770	1.369	1.443
EM-Log	0.2	1.865	1.740	1.352	1.262	1.089	1.016	0.785	0.733	1.230	1.273
EM-Log	0.5	1.915	1.830	1.367	1.307	1.060	1.013	0.766	0.732	1.249	1.277
EM-Log	1.0	1.985	1.930	1.409	1.370	1.084	1.053	0.775	0.753	1.295	1.313
EM-Log	0.5xQ2(σ)	1.911	1.774	1.357	1.260	1.090	1.012	0.797	0.740	1.243	1.289
EM-Log	1xQ2(σ)	1.916	1.811	1.345	1.271	1.063	1.004	0.775	0.733	1.240	1.275
EM-Log	1.5xQ2(σ)	1.915	1.830	1.365	1.304	1.062	1.015	0.767	0.733	1.249	1.277
EM-Log	2xQ2(σ)	1.920	1.848	1.375	1.324	1.059	1.020	0.774	0.745	1.258	1.282
EM-Log	3xQ2(σ)	1.982	1.926	1.409	1.370	1.086	1.055	0.776	0.754	1.295	1.313
EM-Log	6xQ2(σ)	2.059	2.025	1.433	1.410	1.099	1.081	0.777	0.764	1.331	1.342

Q2(σ, ARS)=Q2(σ, ARSS)=0.1350
Q2(σ, EM-N)=0.4651
Q2(σ, EM-LOG)=0.3275

Table S2f CP error rate results, i.e. misclassification rates, on Water internal test set (220), FSTI-XGB model

Error rates	99.00%	95.00%	90.00%	80.00%
AR	0%	2.27%	4.54%	20%
ARS b=0	0%	2.73%	4.55%	20%
ARS b=0.2	0%	1.36%	5.45%	17.27%
ARS b=0.5	0%	2.27%	4.55%	18.18%
ARS b=0.3xQ2(σ)	0%	1.82%	4.09%	17.73%
ARS b=0.5xQ2(σ)	0%	1.36%	4.55%	17.27%
ARS b=1xQ2(σ)	0%	1.82%	5.91%	18.18%
ARS b=1.5xQ2(σ)	0%	1.36%	5.45%	17.27%
ARS b=2xQ2(σ)	0%	1.36%	5.45%	17.27%
ARS b=3xQ2(σ)	0%	1.36%	4.55%	18.64%
ARS b=1.0	0%	2.27%	5%	20%
ARSS b=0	0%	1.82%	4.55%	16.36%
ARSS b=0.2	0%	1.36%	4.55%	18.18%
ARSS b=0.5	0%	1.82%	4.55%	19.55%
ARSS b=1	0%	2.27%	5%	20%
ARSS b=0.3xQ2(σ)	0%	1.82%	4.09%	17.27%
ARSS b=0.5xQ2(σ)	0%	1.36%	5.45%	18.64%
ARSS b=1xQ2(σ)	0%	1.36%	5.45%	18.64%
ARSS b=1.5xQ2(σ)	0%	1.36%	4.55%	18.18%
ARSS b=2xQ2(σ)	0%	1.36%	5%	19.09%
ARSS b=3xQ2(σ)	0%	1.36%	4.55%	18.64%
EM-N b=0	0.45%	4.09%	10.45%	19.09%
EM-N b=0.2	0%	4.09%	9.09%	15.91%
EM-N b=0.5	0%	4.55%	7.27%	17.27%
EM-N b=1.0	0%	3.18%	6.82%	16.82%
EM-N b=0.5xQ2(σ)	0%	5%	9.09%	15.91%
EM-N b=1xQ2(σ)	0%	4.55%	7.27%	17.27%
EM-N b=1.5xQ2(σ)	0%	3.18%	6.82%	16.82%
EM-N b=2xQ2(σ)	0%	3.18%	6.82%	16.82%
EM-N b=3xQ2(σ)	0%	3.18%	6.36%	18.18%
EM-N b=6xQ2(σ)	0%	2.73%	5.91%	19.09%
EM-N b=0.3xQ2(σ)	0.45%	4.09%	9.55%	15.91%
EM-LOG b=0	0.91%	3.64%	9.09%	17.27%
EM-LOG b=0.2	0.45%	3.64%	7.73%	15.91%
EM-LOG b=0.5	0.45%	3.18%	6.36%	16.81%
EM-LOG b=1	0%	3.18%	6.36%	17.73%
EM-LOG b=0.5xQ2(σ)	0.45%	3.64%	8.64%	15.45%
EM-LOG b=1xQ2(σ)	0.45%	3.64%	6.81%	16.36%
EM-LOG b=1.5xQ2(σ)	0.45%	3.18%	6.36%	16.36%
EM-LOG b=2xQ2(σ)	0.45%	3.18%	6.36%	16.36%
EM-LOG b=3xQ2(σ)	0%	3.18%	6.36%	17.73%
EM-LOG b=6xQ2(σ)	0%	3.18%	5.91%	18.18%

Q2(σ, ARS)=Q2(σ, ARSS)=0.1350
Q2(σ, EM-N)=0.4651
Q2(σ, EM-LOG)=0.3275

Table S2g. CP efficiency results, i.e. prediction interval half-widhts, on Water-900 external test set (900) of AqSolDB-n CPs, FSTI-XGB model

Method		99%		95%		90%		80%		average of all	average of
	β	mean	median	mean	median	mean	median	mean	median	8 statistics	all means
AR	-	2.335	2.335	1.534	1.534	1.176	1.176	0.783	0.783	1.457	1.457
ARS	0	2.172	2.078	1.366	1.307	1.065	1.019	0.799	0.764	1.321	1.351
ARS	0.2	2.012	1.976	1.298	1.275	1.035	1.016	0.760	0.747	1.265	1.276
ARS	0.5	2.102	2.082	1.364	1.351	1.070	1.060	0.778	0.771	1.322	1.329
ARS	0.3xQ2(σ)	1.991	1.924	1.319	1.275	1.051	1.015	0.766	0.740	1.259	1.282
ARS	***0.5xQ2(σ)***	***1.935***	***1.878***	***1.276***	***1.238***	*1.033*	*1.003*	*0.764*	*0.742*	*1.234*	1.252
ARS	1xQ2(σ)	2.021	1.76	1.284	1.256	1.028	1.006	0.756	0.740	1.253	1.272
ARS	1.5xQ2(σ)	2.011	1.975	1.298	1.274	1.033	1.014	0.761	0.748	1.275	1.276
ARS	2xQ2(σ)	1.990	1.960	1.336	1.316	1.043	1.027	0.770	0.759	1.273	1.285
ARS	3xQ2(σ)	2.046	2.022	1.351	1.336	1.065	1.053	0.774	0.765	1.306	1.309
ARS	1.0	2.117	2.105	1.438	1.430	1.107	1.101	0.771	0.767	1.355	1.358
ARSS	0	2.169	2.075	1.368	1.309	1.056	1.010	0.793	0.758	1.317	1.347
ARSS	0.2	2.007	1.971	1.321	1.297	1.064	1.044	0.750	0.737	1.274	1.286
ARSS	0.5	2.071	2.051	1.388	1.374	1.082	1.071	0.764	0.756	1.320	1.326
ARSS	1.0	2.117	2.105	1.438	1.430	1.107	1.101	0.771	0.767	1.355	1.358
**ARSS**	**0.5xQ2(σ)**	1.943	1.886	1.310	1.271	**1.029**	**0.999**	0.770	0.748	1.245	1.263
ARSS	0.3xQ2(σ)	1.978	1.911	1.316	1.272	1.048	1.013	0.778	0.752	1.259	1.280
ARSS	1xQ2(σ)	1.966	1.922	1.312	1.283	1.036	1.013	0.754	0.737	1.253	1.267
ARSS	1.5xQ2(σ)	2.006	1.971	1.322	1.298	1.065	1.046	**0.751**	**0.738**	1.275	1.286
ARSS	2xQ2(σ)	1.985	1.956	1.340	1.320	1.083	1.067	0.753	0.742	1.281	1.290
ARSS	3xQ2(σ)	2.027	2.004	1.377	1.361	1.089	1.076	0.763	0.754	1.306	1.314
EM-N	0	2.345	2.127	1.451	1.316	1.175	1.067	0.885	0.803	1.396	1.464
EM-N*	0.2	2.041	1.903	1.351	1.259	1.105	1.030	0.813	0.757	1.282	1.328
EM-N*	0.5	1.908	1.816	1.344	1.279	1.078	1.025	0.783	0.745	1.247	1.278
EM-N	1.0	1.978	1.913	1.379	1.333	1.085	1.049	0.781	0.755	1.284	1.306
EM-N*	0.5xQ2(σ)	2.001	1.871	1.330	1.244	1.100	1.028	0.802	0.750	1.266	1.308
EM-N*	1xQ2(σ)	1.901	1.805	1.334	1.267	1.074	1.020	0.787	0.747	1.242	1.274
EM-N*	1.5xQ2(σ)	1.942	1.863	1.359	1.303	1.075	1.031	0.785	0.753	1.264	1.290
EM-N*	2xQ2(σ)	1.974	1.906	1.370	1.323	1.081	1.044	0.785	0.758	1.280	1.303
EM-N*	3xQ2(σ)	2.015	1.962	1.403	1.366	1.067	1.039	0.776	0.756	1.298	1.315
EM-N	6xQ2(σ)	2.059	2.028	1.442	1.420	1.097	1.080	0.786	0.774	1.336	1.346
EM-N*	0.3xQ2(σ)	2.067	1.914	1.379	1.277	1.108	1.026	0.827	0.766	1.296	1.345
EM-Log	0	2.613	2.332	1.635	1.459	1.329	1.186	0.975	0.870	1.550	1.638
EM-Log*	0.2	2.029	1.881	1.471	1.363	1.185	1.098	0.854	0.792	1.334	1.385
EM-Log*	0.5	2.025	1.925	1.445	1.374	1.121	1.065	0.810	0.770	1.317	1.350
EM-Log	1.0	2.058	1.992	1.460	1.414	1.123	1.087	0.803	0.777	1.339	1.361
EM-Log*	0.5xQ2(σ)	2.090	1.928	1.484	1.369	1.192	1.099	0.871	0.804	1.355	1.409
EM-Log*	1xQ2(σ)	2.053	1.929	1.441	1.354	1.139	1.070	0.831	0.780	1.325	1.366
EM-Log*	1.5xQ2(σ)	2.026	1.925	1.444	1.372	1.123	1.067	0.811	0.771	1.317	1.351
EM-Log*	2xQ2(σ)	2.013	1.929	1.443	1.382	1.111	1.064	0.812	0.778	1.317	1.345
EM-Log*	3xQ2(σ)	2.055	1.989	1.462	1.414	1.126	1.090	0.805	0.779	1.340	1.362
EM-Log	6xQ2(σ)	2.104	2.064	1.464	1.436	1.123	1.101	0.794	0.779	1.358	1.371

Q2(σ, ARS)=Q2(σ, ARSS)=0.1350
Q2(σ, EM-N)=0.4651
Q2(σ, EM-LOG)=0.3275

* The methodology did not pass the error rate validation (i.e. misclassification rate was too high above significance margin), t-test, p<0.01.

Table S2h CP error rate results, i.e. misclassification rates, on Water-900 external test set (900) of AqSolDB-n CPs, FSTI-XGB model

Error rates	99.00%	95.00%	90.00%	80.00%
AR	1.44%	6.11%	10.67%	21.78%
ARS b=0	1.78%	7.44%	12.22%	18.56%
ARS b=0.2	1.78%	7.33%	12.11%	20.56%
ARS b=0.5	1.78%	7.56%	12.11%	20.78%
ARS b=0.3xQ2(σ)	1.89%	7.22%	11.89%	20.22%
ARS b=0.5xQ2(σ)	2.33%	7.44%	12.33%	20.22%
ARS b=1xQ2(σ)	1.78%	7.22%	12.11%	20.89%
ARS b=1.5xQ2(σ)	1.78%	7.44%	12.11%	20.56%
ARS b=2xQ2(σ)	2.33%	7.22%	12.33%	20.56%
ARS b=3xQ2(σ)	2%	7.44%	11.89%	20.89%
ARS b=1.0	1.78%	7.56%	12.11%	20.78%
ARSS b=0	1.78%	7.44%	12.22%	19.00%
ARSS b=0.2	1.89%	7.11%	11.22%	20.78%
ARSS b=0.5	2.00%	7.22%	11.89%	21.22%
ARSS b=1	2%	7.11%	11.44%	21.56%
ARSS b=0.3xQ2(σ)	2%	7.22%	11.89%	20.22%
**ARSS b=0.5xQ2(σ)**	2.22%	7.22%	**12.67%**	20.22%
ARSS b=1xQ2(σ)	2.11%	7.11%	11.89%	20.89%
ARSS b=1.5xQ2(σ)	1.89%	7.11%	11.22%	20.78%
ARSS b=2xQ2(σ)	2.33%	7.11%	11.22%	21.11%
ARSS b=3xQ2(σ)	2%	7.11%	11.89%	21.22%
EM-N b=0	1.78%	7.67%	11.22%	18.11%
EM-N b=0.2	2.22%	*8.44%	11.67%	20.22%
EM-N b=0.5	*2.56%	*8.11%	11.78%	22%
EM-N b=1.0	2.22%	7.33%	11.89%	22%
EM-N b=0.5xQ2(σ)	2.44%	*8.56%	11.67%	20.67%
EM-N b=1xQ2(σ)	*2.67%	*8.33%	12%	21.89%
EM-N b=1.5xQ2(σ)	2.33%	*8.22%	11.78%	22.22%
EM-N b=2xQ2(σ)	2.22%	7.56%	12%	22%
EM-N b=3xQ2(σ)	2.33%	7.11%	12.67%	22.11%
EM-N b=6xQ2(σ)	2.22%	6.67%	12.56%	21.33%
EM-N b=0.3xQ2(σ)	2.33%	*8.22%	11.33%	19.89%
EM-LOG b=0	1.67%	7.56%	12.33%	17.89%
EM-LOG b=0.2	2.89%	*8.44%	12.22%	20.11%
EM-LOG b=0.5	*2.67%	7.89%	12.33%	21.78%
EM-LOG b=1	2.33%	7%	12.11%	21.67%
EM-LOG b=0.5xQ2(σ)	*2.89%	*8.22%	12.44%	19.56%
EM-LOG b=1xQ2(σ)	2.44%	*8.44%	12%	20.78%
EM-LOG b=1.5xQ2(σ)	*2.67%	8%	12.33%	21.67%
EM-LOG b=2xQ2(σ)	*2.78%	7.56%	12.6%	21.67%
EM-LOG b=6xQ2(σ)	2.00%	6.89%	12.44%	21.67%

* The methodology did not pass the error rate validation (i.e. misclassification rate was too high above significance margin), t-test, $p<0.01$.

```
t.test(as.vector(cbind(t(rep(1,200)),t(rep(0,700)))),as.vector(cbind(t(rep(1,180)),t(rep(0,720)))))

        Welch Two Sample t-test

data:  as.vector(cbind(t(rep(1, 200)), t(rep(0, 700)))) and as.vector(cbind(t(rep(1, 180)), t(rep(0, 720))))
t = 1.1549, df = 1795.3, p-value = 0.2483
alternative hypothesis: true difference in means is not equal to 0
95 percent confidence interval:
 -0.01551575  0.05996019
sample estimates:
mean of x mean of y
```

0.2222222 0.2000000

```
> t.test(as.vector(cbind(t(rep(1,114)),t(rep(0,786)))),as.vector(cbind(t(rep(1,90)),t(rep(0,810)))))

        Welch Two Sample t-test

data:  as.vector(cbind(t(rep(1, 114)), t(rep(0, 786)))) and as.vector(cbind(t(rep(1, 90)), t(rep(0, 810))))
t = 1.7851, df = 1779.2, p-value = 0.07442
alternative hypothesis: true difference in means is not equal to 0
95 percent confidence interval:
 -0.002632368  0.055965701
sample estimates:
mean of x mean of y
0.1266667 0.1000000

> t.test(as.vector(cbind(t(rep(1,72)),t(rep(0,828)))),as.vector(cbind(t(rep(1,45)),t(rep(0,855)))))

        Welch Two Sample t-test

data:  as.vector(cbind(t(rep(1, 72)), t(rep(0, 828)))) and as.vector(cbind(t(rep(1, 45)), t(rep(0, 855))))
t = 2.5848, df = 1718.2, p-value = 0.009825
alternative hypothesis: true difference in means is not equal to 0
95 percent confidence interval:
 0.007236116 0.052763884
sample estimates:
mean of x mean of y
     0.08      0.05

> t.test(as.vector(cbind(t(rep(1,77)),t(rep(0,823)))),as.vector(cbind(t(rep(1,45)),t(rep(0,855)))))

        Welch Two Sample t-test

data:  as.vector(cbind(t(rep(1, 77)), t(rep(0, 823)))) and as.vector(cbind(t(rep(1, 45)), t(rep(0, 855))))
t = 3.0065, df = 1696.6, p-value = 0.002682
alternative hypothesis: true difference in means is not equal to 0
95 percent confidence interval:
 0.01235985 0.05875126
sample estimates:
 mean of x  mean of y
0.08555556 0.05000000


> t.test(as.vector(cbind(t(rep(1,24)),t(rep(0,876)))),as.vector(cbind(t(rep(1,9)),t(rep(0,891)))))

        Welch Two Sample t-test

data:  as.vector(cbind(t(rep(1, 24)), t(rep(0, 876)))) and as.vector(cbind(t(rep(1, 9)), t(rep(0, 891))))
t = 2.6391, df = 1497.7, p-value = 0.0084
alternative hypothesis: true difference in means is not equal to 0
95 percent confidence interval:
 0.00427877 0.02905456
sample estimates:
 mean of x  mean of y
0.02666667 0.01000000
```

Table S2i. CP efficiency results, i.e. prediction interval half-widhts, on Water-900 test set (400) for FSTI-XGB model

Method	β	99% mean	99% median	95% mean	95% median	90% mean	90% median	80% mean	80% median	average of all 8 statistics	average of all means
AR	-	2.367	2.367	1.496	1.496	1.230	1.230	0.918	0.918	1.503	1.503
ARSS	0	2.611	2.391	1.603	1.468	1.303	1.194	0.988	0.905	1.558	1.626
ARSS	**0.2**	*2.226*	*2.143*	***1.445***	***1.391***	*1.193*	*1.149*	*0.898*	*0.864*	*1.414*	1.441
ARSS	0.5	2.294	2.247	1.512	1.481	1.171	1.147	0.889	0.871	1.452	1.467
ARSS	1.0	2.314	2.287	1.510	1.493	1.180	1.167	0.908	0.898	1.470	1.478
ARSS	0.5xQ2(σ)	2.257	2.126	1.521	1.433	1.242	1.170	0.910	0.858	1.440	1.483
ARSS	1.0xQ2(σ)	2.243	2.144	1.473	1.408	1.216	1.162	0.904	0.864	1.427	1.459
ARS	0	2.665	2.441	1.661	1.521	1.307	1.197	0.956	0.876	1.578	1.647
ARS	0.2xQ2(σ)	2.370	2.201	1.573	1.461	1.242	1.153	0.924	0.858	1.473	1.527
ARS	0.4xQ2(σ)	2.219	2.082	1.565	1.468	1.234	1.158	0.897	0.841	1.433	1.479
ARS	0.5xQ2(σ)	**2.177**	**2.050**	1.536	1.447	1.240	1.168	0.899	0.847	1.421	1.463
ARS	0.6xQ2(σ)	2.184	2.065	1.527	1.444	1.230	1.163	0.903	0.854	1.421	1.461
ARS	0.7xQ2(σ)	2.211	2.097	1.528	1.449	1.229	1.166	0.908	0.861	1.431	1.469
ARS	1.0xQ2(σ)	2.282	2.181	1.500	1.434	1.221	1.167	0.984	0.854	1.453	1.497
EM-N	0	2.520	2.328	1.683	1.555	1.321	1.220	0.985	0.910	1.565	1.627
EM-N	0.2	2.359	2.223	1.569	1.479	1.241	1.169	0.919	0.866	1.478	1.522
EM-N	0.5	2.343	2.244	1.477	1.414	1.183	1.133	0.922	0.883	1.450	1.481
EM-N	1.0	2.396	2.326	1.472	1.429	**1.155**	**1.121**	0.940	0.913	1.469	1.491
EM-Log	0	2.676	2.597	1.755	1.703	1.330	1.290	1.013	0.983	1.668	1.694
EM-Log	0.2	2.246	2.200	1.593	1.560	1.219	1.194	0.911	0.892	1.477	1.492
EM-Log	0.5	2.311	2.279	1.513	1.493	1.189	1.173	0.917	0.905	1.473	1.483
EM-Log	1	2.305	2.284	1.469	1.456	1.193	1.182	0.922	0.914	1.466	1.472
EM-EXP	0	2.737	2.227	1.740	1.415	1.374	1.118	1.054	0.857	1.565	1.726
EM-EXP	0.2	2.688	2.229	1.686	1.397	1.340	1.111	1.031	0.855	1.542	1.686
EM-EXP	0.5	2.652	2.249	1.645	1.395	1.294	1.097	1.004	0.852	1.524	1.649
EM-EXP	1.0	2.619	2.283	1.636	1.427	1.281	1.116	0.995	0.867	1.528	1.633
knn-EuD	0	2.557	2.322	1.720	1.562	1.294	1.175	0.929	0.843	1.550	1.625
knn-EuD	1xQ2(σ)	2.506	2.383	1.596	1.517	1.234	1.174	0.890	0.847	1.518	1.557
knn-EuD	2xQ2(σ)	2.500	2.417	1.539	1.488	1.205	1.165	**0.879**	**0.850**	1.505	1.531
knn-EuD	6xQ2(σ)	2.424	2.389	1.468	1.447	1.181	1.164	0.897	0.884	1.482	1.493

Q2(σ, ARS)=Q2(σ, ARSS)=0.1448
Q2(σ, EM-N)=0.5506
Q2(σ, EM-LOG)=0.3922
Q2(σ, EM-EXP)=1.7264

Table S2j. CP error rate results, i.e. misclassification rates, on Water-900 test set (400) for FSTI-XGB model

Error rates	99.00%	95.00%	90.00%	80.00%
AR	2.75%**	7.00%	12.75%	20.50%
ARSS b=0	1.25%	5.00%	9.25%	18.50%
ARSS b=0.2 (1.4xQ2(σ))	2.25%	6.75%	10.00%	20.50%
ARSS b=0.5	2.50%	6.00%	12.50%	21.25%
ARSS b=1	2.50%	6.00%	13.75%*	20.25%
ARSS b=0.5xQ2(σ)	2.00%	6.75%	9.00%	20.50%
ARSS b=1xQ2(σ)	2.00%	6.75%	9.00%	20.50%
ARS b=0	1.25%	4.75%	9.25%	19.50%
ARS b=0.2xQ2(σ)	1.75%	5.50%	9.75%	20.50%
ARS b=0.4xQ2(σ)	2.00%	5.75%	9.00%	20.50%
ARS b=0.5xQ2(σ)	2.00%	6.25%	8.75%	20.75%
ARS b=0.6xQ2(σ)	2.00%	6.25%	8.75%	20.50%
ARS b=0.7xQ2(σ)	2.00%	6.25%	8.75%	20.50%
ARS b=1xQ2(σ)	2.00%	6.50%	8.75%	20.75%
EM-N b=0	2.00%	6.50%	12.25%	21.25%
EM-N b=0.2	2.25%	6.75%	12.25%	21.50%
EM-N b=0.5	2.00%	7.50%	12.50%	21.25%
EM-N b=1.0	2.00%	6.50%	12.25%	21.25%
EM-LOG b=0	1.75%	6.50%	12.00%	20.25%
EM-LOG b=0.2	2.00%	5.75%	12.00%	22.50%
EM-LOG b=0.5	2.25%	7.25%	12.00%	21.00%
EM-LOG b=1	2.25%	7.5%***	12.00%	20.50%
EM-EXP b=0	2.00%	7.00%	12.75%	21.75%
EM-EXP b=0.2	2.00%	7.25%	13.00%	21.75%
EM-EXP b=0.5	2.00%	7.5%***	13.00%	21.50%
EM-EXP b=1.0	2.00%	7.5%***	13.00%	20.75%
kNN-EuD b=0	2.75**	6.50%	11.25%	21.50%
kNN-EuD b=1 x Q2(σ)	2.50%	5.50%	11.75%	22.25%
kNN-EuD b=2 x Q2(σ)	2.50%	6.25%	12.50%	22.00%
kNN-EuD b=6 x Q2(σ)	2.50%	7.00%	14.25%****	20.75%

Q2(σ, ARS)=Q2(σ, ARSS)=0.1448
Q2(σ, EM-N)=0.5506
Q2(σ, EM-LOG)=0.3922
Q2(σ, EM-EXP)=1.7264

*

```
> t.test(t,h,var.equal=TRUE)

	Two Sample t-test

data:  t and h
t = 1.6401, df = 798, p-value = 0.1014
alternative hypothesis: true difference in means is not equal to 0
95 percent confidence interval:
 -0.007381926  0.082381926
sample estimates:
mean of x mean of y 
   0.1375    0.1000 
```

**

```
> t.test(t,h,var.equal=TRUE)
data:  t and h
t = 1.8261, df = 798, p-value = 0.06821
alternative hypothesis: true difference in means is not equal to 0
95 percent confidence interval:
 -0.001311386  0.036311386
sample estimates:
mean of x mean of y 
```

0.0275 0.0100

***
> t.test(t,h,var.equal=TRUE)

data: t and h
t = 1.4607, df = 798, p-value = 0.1445
alternative hypothesis: true difference in means is not equal to 0
95 percent confidence interval:
-0.008595542 0.058595542
sample estimates:
mean of x mean of y
0.075 0.050

****
Two Sample t-test

data: t and h
t = 1.8429, df = 798, p-value = 0.06571
alternative hypothesis: true difference in means is not equal to 0
95 percent confidence interval:
-0.002767565 0.087767565
sample estimates:
mean of x mean of y
0.1425 0.1000

Table S2k. CP efficiency results, i.e. prediction interval half-widhts, on Ethanol test set (139) for FSTI-XGB model

Method		99%		95%		90%		80%		average of all 8 statistics	average of all means
	β	mean	median	mean	median	mean	median	mean	median		
AR	-	2.034	2.034	1.361	1.361	1.040	1.040	**0.732**	**0.732**	1.292	1.292
ARSS	0	2.266	2.039	1.435	1.291	1.141	1.027	0.815	0.734	1.344	1.414
ARSS	0.2	2.097	2.003	1.343	1.282	1.080	1.031	0.742	0.709	1.286	1.316
ARSS	0.5	2.035	1.985	1.346	1.313	1.042	1.017	0.738	0.720	1.275	1.290
ARSS	1.0	2.034	2.006	1.350	1.331	1.031	1.017	0.737	0.727	1.279	1.288
ARSS	0.5xQ2(σ)	2.201	2.051	1.348	1.256	1.062	0.990	0.771	0.718	1.300	1.346
ARSS	1.0xQ2(σ)	2.153	2.041	1.340	1.270	1.070	1.015	0.754	0.715	1.295	1.329
EM-N	0	2.366	2.007	1.479	1.250	1.163	0.986	0.892	0.756	1.362	1.475
EM-N	0.2	2.056	1.831	1.418	1.263	1.062	0.946	0.813	0.724	1.264	1.337
EM-N	***0.5***	*1.887*	*1.742*	*1.382*	*1.276*	***1.014***	***0.936***	*0.787*	*0.727*	*1.219*	1.268
EM-N	1.0	**1.832**	**1.737**	1.385	1.313	1.020	0.967	0.778	0.738	1.221	1.254
EM-Log	0	2.340	2.009	1.493	1.282	1.167	1.002	0.871	0.748	1.364	1.468
EM-Log	0.2	2.040	1.856	1.367	1.243	1.017	0.925	0.806	0.734	1.249	1.308
EM-Log	0.5	1.961	1.847	1.385	1.304	1.014	0.955	0.779	0.734	1.247	1.285
EM-Log	1.0	1.934	1.863	1.357	1.307	1.033	0.995	0.764	0.736	1.249	1.272
EM-Log	1.1xQ2(σ)	1.998	1.853	1.402	1.301	1.024	0.950	0.792	0.735	1.257	1.304
ARS	0	2.248	2.023	1.399	1.256	1.163	1.047	0.823	0.741	1.338	1.408
ARS	0.2	2.048	1.956	1.321	1.261	1.044	0.997	0.763	0.729	1.265	1.294
ARS	0.5	2.013	1.963	1.356	1.323	1.039	1.014	0.763	0.745	1.277	1.293
ARS	1.0	1.990	1.962	1.354	1.335	1.023	1.008	0.770	0.760	1.275	1.284
ARS	0.5xQ2(σ)	2.136	1.990	**1.318**	**1.228**	1.061	0.988	0.783	0.729	1.279	1.325
ARS	1.0xQ2(σ)	2.085	1.977	1.320	1.252	1.054	0.999	0.775	0.734	1.275	1.309

Q2(σ, ARS)=Q2(σ, ARSS)=0.1510
Q2(σ, EM-N)=0.4516
Q2(σ, EM-LOG)=0.3012

Table S2l. CP error rate results, i.e. misclassification rates, on Ethanol test set (139) for FSTI-XGB model

Error rates	99.00%	95.00%	90.00%	80.00%
AR	2.16%	7.19%	16.55%	30.93%
ARSS b=0	2.16%	7.91%	14.39%	30.22%
ARSS b=0.2	2.16%	7.19%	10.79%	33.81%**
ARSS b=0.5	2.16%	7.19%	15.11%	31.65%*
ARSS b=1	1.44%	7.19%	15.83%	30.93%
ARSS b=0.5*Q2(σ)	2.16%	7.91%	12.95%	32.37%
ARSS b=1*Q2(σ)	2.16%	7.91%	10.79%	32.37%
EM-N b=0	2.16%	8.63%	16.55%	25.18%
EM-N b=0.2	2.16%	7.19%	14.39%	30.93%
EM-N b=0.5 (1.107xQ2(σ))	2.88%	6.47%	13.67%	30.21%
EM-N b=1.0	3.60%	6.47%	13.67%	29.50%
EM-LOG b=0	2.88%	11.51%	17.99%	25.18%
EM-LOG b=0.2	2.88%	9.35%	16.55%	27.34%
EM-LOG b=0.5	2.88%	7.19%	12.95%	29.50%
EM-LOG b=1.0	2.88%	7.19%	12.95%	29.50%
EM-LOG b=1.107*Q2(σ)	2.88%	7.19%	12.95%	30.22%
ARS b=0	2.16%	7.91%	14.39%	29.50%
ARS b=0.2	2.16%	7.19%	12.95%	32.37%
ARS b=0.5	2.16%	7.19%	15.11%	29.50%
ARS b=1	2.16%	7.19%	15.83%	30.22%
ARS b=0.5*Q2(σ)	2.16%	7.91%	12.95%	31.65%
ARS b=1*Q2(σ)	2.16%	7.91%	12.95%	30.94%

Q2(σ, ARS)=Q2(σ, ARSS)=0.1510
Q2(σ, EM-N)=0.4516
Q2(σ, EM-LOG)=0.3012

*

```
> t.test(t,h,var.equal=TRUE)

Two Sample t-test

data:  t and h
t = 2.2017, df = 276, p-value = 0.02851
alternative hypothesis: true difference in means is not equal to 0
95 percent confidence interval:
 0.01218632 0.21802951
sample estimates:
mean of x mean of y
0.3165468 0.2014388
```

**

```
> t.test(t,h,var.equal=TRUE)

Two Sample t-test

data:  t and h
t = 2.589, df = 276, p-value = 0.01013
alternative hypothesis: true difference in means is not equal to 0
95 percent confidence interval:
 0.03275724 0.24062406
sample estimates:
mean of x mean of y
0.3381295 0.2014388
```

Table S2m. CP efficiency results, i.e. prediction interval half-widhts, on Acetone test set (91) for FSTI-XGB model

Method		99%		95%		90%		80%		average of all	average of
	β	mean	median	mean	median	mean	median	mean	median	8 statistics	all means
AR	-	1.801	1.801	1.344	1.344	1.047	1.047	0.741	0.741	1.233	1.233
ARSS	0	2.220	2.024	1.424	1.299	1.170	1.067	0.833	0.760	1.350	1.412
ARSS	0.2	**1.797**	**1.737**	1.302	1.259	1.087	1.051	0.739	0.714	1.211	1.231
ARSS	0.5	1.810	1.778	1.323	1.300	1.064	1.046	0.742	0.729	1.224	1.235
ARSS	1.0	1.801	1.784	1.324	1.311	1.063	1.053	0.740	0.733	1.226	1.232
ARSS	0.5xQ2(σ)	1.912	1.793	1.362	1.278	1.089	1.022	0.788	0.739	1.248	1.288
ARSS	1.0xQ2(σ)	1.858	1.769	1.319	1.256	1.079	1.028	0.748	0.713	1.221	1.251
EM-N	0	2.100	1.959	1.394	1.301	1.061	0.990	0.744	0.695	1.281	1.325
EM-N	0.2	2.051	1.954	1.290	1.229	1.033	0.985	0.728	0.693	1.245	1.276
EM-N	0.5	2.001	1.936	1.243	1.202	1.006	0.974	0.705	0.682	1.219	1.239
EM-N	1.0	1.941	1.899	1.241	1.214	1.009	0.987	0.704	0.689	1.211	1.224
EM-N	*0.75xQ2(σ)*	*2.025*	*1.801*	*1.260*	*1.211*	***1.001***	***0.962***	*0.712*	*0.685*	*1.207*	1.250
EM-N	1.5xQ2(σ)	1.976	1.922	**1.240**	**1.206**	1.006	0.979	**0.701**	**0.682**	1.214	1.231
EM-Log	0	2.198	2.096	1.469	1.400	1.166	1.111	0.802	0.765	1.376	1.409
EM-Log	0.2	2.006	1.948	1.300	1.263	1.069	1.038	0.761	0.739	1.266	1.284
EM-Log	0.5	1.967	1.931	1.311	1.287	1.033	1.014	0.743	0.729	1.252	1.264
EM-Log	1.0	1.917	1.895	1.278	1.263	1.011	0.999	0.723	0.715	1.225	1.232
EM-Log	0.75xQ2(σ)	1.997	1.942	1.299	1.263	1.052	1.023	0.755	0.734	1.258	1.276
EM-Log	1.5xQ2(σ)	1.976	1.937	1.313	1.287	1.036	1.016	0.748	0.733	1.256	1.268
ARS	0	2.219	2.023	1.445	1.317	1.141	1.041	0.857	0.782	1.353	1.416
ARS	0.2	1.869	1.807	1.314	1.270	1.069	1.033	0.747	0.722	1.229	1.250
ARS	0.5	1.906	1.873	1.307	1.284	1.060	1.042	0.752	0.739	1.245	1.256
ARS	1.0	1.958	1.939	1.348	1.335	1.042	1.032	0.739	0.732	1.266	1.272
ARS	0.5xQ2(σ)	2.036	1.910	1.428	1.340	1.080	1.013	0.778	0.730	1.289	1.331
ARS	1.0xQ2(σ)	1.934	1.842	1.390	1.324	1.069	1.018	0.745	0.709	1.254	1.285

Q2(σ, ARS)=Q2(σ, ARSS)=0.1040
Q2(σ, EM-N)=0.4428
Q2(σ, EM-LOG)=0.2991

Table S2n. CP error rate results, i.e. misclassification rates, on Acetone test set (91) for FSTI-XGB model

Error rates	99.00%	95.00%	90.00%	80.00%
AR	1.10%	6.59%	14.29%	28.57%*
ARSS b=0	0.00%	6.59%	10.99%	23.08%
ARSS b=0.2	0	5.49%	14.29%	24.18%
ARSS b=0.5	0	5.49%	14.29%	24.18%
ARSS b=1	0	5.49%	14.29%	24.18%
ARSS b=0.5*Q2(σ)	0	5.49%	14.29%	21.98%
ARSS b=1*Q2(σ)	0	5.49%	13.19%	24.18%
EM-N b=0	0	4.40%	12.09%	29.67%
EM-N b=0.2	0	3.30%	12.09%	31.87%**
EM-N b=0.5	0	5.49%	15.38%	30.77%
EM-N b=1.0	0	5.49%	15.38%	30.77%
EM-N b=0.75*Q2(σ)	0	4.40%	15.38%	30.77%
EM-N b=1.5*Q2(σ)	0	5.49%	15.38%	30.77%
EM-LOG b=0	1.10%	2.20%	8.79%	24.18%
EM-LOG b=0.2	1.10%	4.40%	9.89%	26.37%
EM-LOG b=0.5	0	3.30%	13.19%	29.67%
EM-LOG b=1.0	0	6.59%	15.38%	30.77%
EM-LOG b=0.75*Q2(σ)	1.10%	4.40%	12.09%	28.57%
EM-LOG b=1.5*Q2(σ)	0	3.30%	13.19%	29.67%
ARS b=0	0	6.59%	10.99%	23.08%
ARS b=0.2	0	4.40%	13.19%	24.18%
ARS b=0.5	0	6.59%	14.29%	24.18%
ARS b=1	0	5.49%	15.38%	28.57%
ARS b=0.5*Q2(σ)	0	4.40%	14.29%	22.00%
ARS b=1*Q2(σ)	0	2.20%	13.19%	24.18%

Q2(σ, ARS)=Q2(σ, ARSS)=0.1040
Q2(σ, EM-N)=0.4428
Q2(σ, EM-LOG)=0.2991

*

```
>  t.test(vect_telta_80,vect_pred_y_80,var.equal=TRUE)

 Two Sample t-test

data:  vect_telta_80 and vect_pred_y_80
t = -1.3847, df = 180, p-value = 0.1679
alternative hypothesis: true difference in means is not equal to 0
95 percent confidence interval:
 -0.21318732  0.03736314
sample estimates:
mean of x mean of y
0.1978022 0.2857143
```

**

```
> t.test(vect_telta_80,vect_pred_y_80,var.equal=TRUE)

 Two Sample t-test

data:  vect_telta_80 and vect_pred_y_80
t = -1.8707, df = 180, p-value = 0.06302
alternative hypothesis: true difference in means is not equal to 0
95 percent confidence interval:
 -0.248386417  0.006628176
sample estimates:
mean of x mean of y
0.1978022 0.3186813
```

Table S2o. CP efficiency results, i.e. prediction interval half-widhts, on Methanol test set (36) for FSTI-XGB model

Method		99%		95%		90%		80%		average of all	average of
	β	mean	median	mean	median	mean	median	mean	median	8 statistics	all means
AR	-	2.392	2.392	1.351	1.351	1.194	1.194	0.810	0.810	1.437	1.437
ARSS	0	7.794	7.686	1.569	1.547	1.275	1.258	1.073	1.058	2.908	2.928
ARSS	0.2	2.828	2.811	1.349	1.340	1.209	1.202	0.865	0.859	1.558	1.563
ARSS	0.5	2.561	2.552	1.367	1.363	1.187	1.183	0.854	0.851	1.490	1.492
ARSS	1.0	2.485	2.480	1.377	1.375	1.157	1.155	0.831	0.830	1.461	1.463
ARSS	0.5xQ2(σ)	3.976	3.937	1.355	1.342	1.214	1.202	0.973	0.963	1.870	1.880
ARSS	1.0xQ2(σ)	3.155	3.132	1.345	1.335	1.199	1.191	0.887	0.881	1.641	1.647
ARSS	2.0xQ2(σ)	2.657	2.643	1.340	1.333	1.218	1.212	0.862	0.857	1.515	1.519
ARSS	3.0xQ2(σ)	2.587	2.578	1.359	1.354	1.198	1.194	0.848	0.845	1.495	1.498
ARSS	6.0xQ2(σ)	2.502	2.496	1.382	1.379	1.161	1.159	0.836	0.834	1.469	1.470
ARS	0	7.812	7.703	1.600	1.578	1.242	1.225	1.047	1.033	2.905	2.925
ARS	0.2	2.835	2.817	1.300	1.292	1.079	1.072	0.856	0.851	1.513	1.518
ARS	0.5	2.491	2.482	1.324	1.320	**1.041**	**1.038**	0.833	0.831	1.420	1.422
ARS	1.0	2.417	2.413	1.337	1.334	1.071	1.069	0.808	0.807	1.407	1.408
ARS	2.0xQ2(σ)	2.602	2.589	1.309	1.303	1.055	1.050	0.840	0.836	1.448	1.452
ARS	3.0xQ2(σ)	2.517	2.507	1.320	1.315	1.045	1.041	0.834	0.831	1.426	1.429
ARS	6.0xQ2(σ)	2.433	2.428	1.334	1.331	1.058	1.056	0.814	0.812	1.408	1.410
ARS	10xQ2(σ)	2.394	2.391	1.341	1.339	1.066	1.065	**0.802**	**0.801**	1.400	1.401
EM-N	0	2.192	2.112	1.514	1.458	1.285	1.237	0.891	0.858	1.443	1.471
EM-N	0.2	**1.938**	**1.886**	1.448	1.409	1.172	1.140	0.841	0.819	1.332	1.350
EM-N	0.5	2.034	1.994	1.339	1.313	1.181	1.158	0.841	0.825	1.336	1.349
EM-N	***1.0***	*2.117*	*2.090*	***1.232***	***1.216***	*1.147*	*1.132*	*0.837*	*0.826*	*1.325*	1.333
EM-N	1.5xQ2(σ)	2.084	2.052	1.274	1.254	1.156	1.138	0.842	0.829	1.329	1.339
EM-N	3.0xQ2(σ)	2.164	2.143	1.243	1.231	1.120	1.109	0.849	0.841	1.338	1.344
EM-N	4.0xQ2(σ)	2.193	2.175	1.255	1.246	1.109	1.100	0.848	0.841	1.346	1.351
EM-N	6.0xQ2(σ)	2.226	2.214	1.270	1.262	1.118	1.112	0.826	0.822	1.356	1.360
EM-N	1.0xQ2(σ)	2.035	1.996	1.336	1.311	1.180	1.157	0.841	0.824	1.335	1.348
EM-N	0.5xQ2(σ)	1.959	1.910	1.440	1.404	1.154	1.125	0.848	0.826	1.333	1.350
EM-Log	0	3.488	3.062	1.944	1.707	1.414	1.241	1.022	0.897	1.847	1.967
EM-Log	0.2	2.111	1.933	1.532	1.403	1.225	1.121	0.929	0.851	1.388	1.449
EM-Log	0.5	2.173	2.048	1.392	1.312	1.191	1.122	0.888	0.837	1.370	1.411
EM-Log	1.0	2.220	2.137	1.305	1.256	1.133	1.090	0.882	0.849	1.359	1.385
EM-Log	1.0xQ2(σ)	2.152	2.009	1.402	1.309	1.217	1.136	0.901	0.841	1.371	1.418
EM-Log	1.5xQ2(σ)	2.181	2.063	1.388	1.313	1.181	1.117	0.888	0.840	1.371	1.410
EM-Log	2.0xQ2(σ)	2.201	2.101	1.348	1.286	1.157	1.105	0.887	0.846	1.366	1.398
EM-Log	3.0xQ2(σ)	2.228	2.150	1.291	1.246	1.128	1.089	0.873	0.842	1.356	1.380
EM-Log	6.0xQ2(σ)	2.264	2.218	1.286	1.260	1.096	1.074	0.838	0.821	1.357	1.371

Q2(σ, ARS)=Q2(σ, ARSS)=0.1388
Q2(σ, EM-N)=0.5058
Q2(σ, EM-LOG)=0.3735

Table S2p. CP error rate results, i.e. misclassification rates, on Methanol test set (36) for FSTI-XGB model

Error rates	99.00%	95.00%	90.00%	80.00%
AR	0.00%	11.11%	11.11%	30.56%
ARSS b=0	0.00%	2.78%	8.33%	11.11%
ARSS b=0.2	0.00%	5.56%	8.33%	16.67%
ARSS b=0.5	0.00%	8.33%	11.11%	19.44%
ARSS b=1	0.00%	8.33%	11.11%	25.00%
ARSS b=0.5*Q2(σ)	0.00%	5.56%	8.33%	16.67%
ARSS b=1*Q2(σ)	0.00%	5.56%	8.33%	16.67%
ARSS b=2*Q2(σ)	0.00%	5.56%	8.33%	16.67%
ARSS b=3*Q2(σ)	0.00%	8.33%	8.33%	22.22%
ARSS b=6*Q2(σ)	0.00%	8.33%	11.11%	22.22%
ARS b=0	0.00%	2.78%	8.33%	13.89%
ARS b=0.2	0.00%	5.56%	13.89%	16.67%
ARS b=0.5	0.00%	8.33%	13.89%	22.22%
ARS b=1	0.00%	8.33%	11.11%	30.56%
ARS b=2*Q2(σ)	0.00%	8.33%	13.89%	22.22%
ARS b=3*Q2(σ)	0.00%	8.33%	13.89%	22.22%
ARS b=6*Q2(σ)	0.00%	8.33%	13.89%	30.56%
ARS b=10*Q2(σ)	0.00%	8.33%	11.11%	30.56%
EM-N b=0	0.00%	2.78%	13.89%	22.22%
EM-N b=0.2	0.00%	5.56%	13.89%	22.22%
EM-N b=0.5	0.00%	5.56%	11.11%	22.22%
EM-N b=1.0	0.00%	8.33%	8.33%	22.22%
EM-N b=1.5*Q2(σ)	0.00%	8.33%	11.11%	22.22%
EM-N b=3*Q2(σ)	0.00%	8.33%	11.11%	22.22%
EM-N b=4*Q2(σ)	0.00%	8.33%	11.11%	22.22%
EM-N b=6*Q2(σ)	0.00%	8.33%	11.11%	25.00%
EM-N b=1*Q2(σ)	0.00%	5.56%	11.11%	22.22%
EM-N b=0.5*Q2(σ)	0.00%	5.56%	13.89%	22.22%
EM-LOG b=0	0.00%	2.78%	8.33%	16.67%
EM-LOG b=0.2	2.78%	5.56%	8.33%	19.44%
EM-LOG b=0.5	0.00%	5.56%	8.33%	22.22%
EM-LOG b=1.0	0.00%	8.33%	8.33%	22.22%
EM-LOG b=1.0*Q2(σ)	2.78%	5.56%	8.33%	22.22%
EM-LOG b=1.5*Q2(σ)	0.00%	5.56%	8.33%	22.22%
EM-LOG b=2*Q2(σ)	0.00%	8.33%	8.33%	22.22%
EM-LOG b=3*Q2(σ)	0.00%	8.33%	8.33%	22.22%
EM-LOG b=6*Q2(σ)	0.00%	8.33%	11.11%	25.00%

Q2(σ, ARS)=Q2(σ, ARSS)=0.1388
Q2(σ, EM-N)=0.5058
Q2(σ, EM-LOG)=0.3735

```
> t.test(as.vector(cbind(t(rep(1,7)),t(rep(0,29)))),as.vector(cbind(t(rep(1,11)),t(rep(0,25)))),var.equal=TRUE)

        Two Sample t-test

data:  as.vector(cbind(t(rep(1, 7)), t(rep(0, 29)))) and as.vector(cbind(t(rep(1, 11)), t(rep(0, 25))))
t = -1.0824, df = 70, p-value = 0.2828
alternative hypothesis: true difference in means is not equal to 0
95 percent confidence interval:
 -0.31584879  0.09362657
sample estimates:
mean of x mean of y
0.1944444 0.3055556
```

```
> t.test(t,h,var.equal=TRUE)

        Two Sample t-test

data:  t and h
t = 0.35167, df = 70, p-value = 0.7261
alternative hypothesis: true difference in means is not equal to 0
95 percent confidence interval:
 -0.1297572  0.1853128
sample estimates:
mean of x mean of y
0.1388889 0.1111111

> t.test(t,h,var.equal=TRUE)

        Two Sample t-test

data:  t and h
t = 0.4578, df = 70, p-value = 0.6485
alternative hypothesis: true difference in means is not equal to 0
95 percent confidence interval:
 -0.09323802  0.14879357
sample estimates:
 mean of x  mean of y
0.08333333 0.05555556

> t.test(t,h,var.equal=TRUE)

        Two Sample t-test

data:  t and h
t = 1, df = 70, p-value = 0.3208
alternative hypothesis: true difference in means is not equal to 0
95 percent confidence interval:
 -0.02762325  0.08317881
sample estimates:
 mean of x  mean of y
0.02777778 0.00000000
```

Info section S4: CP estimation of the LogS±1 Accuracy on Databases void of experimental

LogS Calculation of Drugbank (total 11370 compounds, 11130 within AD)

Part 1. Calculation of FSTI-XGB-ARSS model for AqSolDB-n (Aq-n of 10,413 compounds) :

median 80% prediction interval:
> median(pred_y_half_width_80perc_AD2)
[1] 0.8878234 - Comment: so half of 10413 compounds have LogS half-width narrower and the other half wider error margin than 0.8878234 LogS a7 80% conf. level
> quantile(pred_y_half_width_80perc_AD2) # This is actually percentile analysis
      0%   25%   50%   75%   100%
0.4703049 0.7858999 0.8878234 1.0080010 1.8880263
> quantile(pred_y_half_width_80perc_AD2,c(0.74))
     74%
1.002856
> quantile(pred_y_half_width_80perc_AD2,c(0.73))
      73% - 73rd percentile has 0.9977111 LogS half-width
0.9977111 Conclusion: 73% of comps have LogS±1 accuracy >80%

> quantile(pred_y_half_width_80perc_AD2,c(0.736))
0.9999013

0.73* length(pred_y_half_width_80perc_AD2)/11370 =
= 66.9% of total Drugbank are within 1logS at 80% conf. level #Comment: This is to explain how I obtained 67% of Upper left Drugbank dark green part of pie -it takes into account 11370 compounds. It should NOT be confused with AqSolDB-n AD selected compounds (10413 compounds) which make 73.8% (Table 8).

> quantile(pred_y_half_width_**90perc**_AD2,c(0.1,0.2,0.3,0.4,0.45)) Comment: this is 90% conf. level analysis
     10%   20%   30%   40%   45%
1.002190 1.080600 1.141776 1.201261 1.228098
> quantile(pred_y_half_width_**90perc**_AD2,c(0.09,0.2,0.3,0.4,0.45))
       9%   20%   30%   40%   45%
0.9933634 1.0805998 1.1417762 1.2012606 1.2280976
> quantile(pred_y_half_width_90perc_AD2,c(0.08,0.09,0.1))
       8%   **9%**   10%
0.9799835 **0.9933634** 1.0021904 Conclusion: 9% of comps have LogS±1 accuracy >90%

> median(pred_y_half_width_70perc_AD2)
[1] 0.7095413
> quantile(pred_y_half_width_70perc_AD2)
       0%   25%   50%   75%   100%
0.3758639 0.6280849 0.7095413 0.8055863 1.5088955
> quantile(pred_y_half_width_70perc_AD2,c(0.9,0.95,0.96,0.97))
      90%   95%   **96%**   97%
0.9072591 0.9777004 **0.9968375** 1.0223545 Conclusion: 96% of comps have LogS±1 accuracy > 70% (i.e. the weakest 4% have accuracy < 70%)
pred_y_half_width_92perc_AD2<-st_d_y_AD2_2*(sort((abs(telta-B1)/STDEV_CPcv_2),decreasing=TRUE)
[(length(B1)+1) %/% (100/8)]) *fact90
> quantile(pred_y_half_width_92perc_AD2,c(0.02,0.025,0.03,0.04,0.05,0.06))
       2%   2.5%   3%   **4%**   5%   6%
0.9221746 0.9489805 0.9760705 **1.0066086** 1.0267221 1.0433343 Conclusion: 4% of comps have LogS±1 accuracy >92%
> quantile(pred_y_half_width_**95perc**_AD2,c(0.01,0.015,0.02))
       1%   **1.5%**   2%
0.9583413 **1.0079200** 1.0479614 Conclusion: 1.5% of comps have LogS±1 accuracy >95%

Acc. of 0-1.5% comps >95%
Acc. of 1.5%-4% comps >92%

Acc. of 4%-9% comps >90% (average between 90 and 92 = 91)
So, 5% (9%-4%=5%) of AqSolDB-n have estimated acc. of 91% LogS±1
Acc. of 9%-73% comps >80% (85% average acc.)
So, 64% (73%-9%=64%) of AqSolDB-n have estimated acc. of 85% acc.
Accc. of 73%-96% comps > 70% (average between 80 and 70 = 75%)
So, 23% (96%-73%=23%) of AqSolDB-n have estimated acc. of 75% acc.
96-100% < 70%
4% above 92% acc. and 4% below 70% acc. are approximated to be disregarded.
> (0.91*0.05+0.85*0.64+0.75*0.23)/(0.05+0.64+0.23)
[1] 0.8282609 That is accuracy of the AqSolDB-n (having 10413 comps out of 11130)

Part 2. Calculation of FSTI-XGB-EM-N model for AqSolDB-w (Aq-w of 717 compounds):

median(pred_y_half_width_80perc_AD2)
1.478717
mean(pred_y_half_width_80perc_AD2)
1.492991
> pred_y_half_width_633perc<- error_te_DB*(sort(alfa_value,decreasing=TRUE)[length(B1) %/% (100/367)])
*fact633
> mean(pred_y_half_width_80perc)/mean(pred_y_half_width_633perc)
[1] 1.49263
mean AqSolDB-w at 90% conf. is 1.492991.
So, 1.49263 ≈ 1.492991
So, 63.3% is estimated accuracy of AqSolDB-w (having 717 comps out of 11130)

Part 3. Final calculation Aq-n + Aq-w

Therefore
> (10413*0.8283 + 717*0.633)/(10413+717)

[1] 0.81571 (i.e. 81.57%) - total accuracy of all Drugbank comps with AD (11130)

Overview of the overall Drugbank accuracy statistics:
717 – 63.7% acc.
4% <70% acc.   4%   70% > %logS ±1
23% - 75% acc.   23%   70% < %logS ±1 < 80% (average 75% acc.)
64% - 85%   64%   80% < %logS ±1 < 90% (average 85% acc.)
9% > 90% acc.   9%   %logS ±1 > 90%
5% ca. 91% acc.   4%   %logS ±1 > 92% (average for 5% comps is 91% acc.)
4% >92% acc.

Calculation of PubChem (total 72739 compounds, 71808 within AD)

Part 1. Calculation of FSTI-XGB-ARSS model for AqSolDB-n (Aq-n of 68,535 compounds):

> median(pred_y_half_width_80perc_AD2) # 80% conf. level
[1] 0.8851128
> quantile(pred_y_half_width_80perc_AD2)
0%   25%   50%   75%   100%
0.4731928 0.7946685 0.8851128 0.9978667 2.0995683 Conclusion: 75% of comps have LogS±1 accuracy >80%

> quantile(pred_y_half_width_90perc_AD2,c(0.06,0.2,0.3,0.4,0.45))
6%   20%   30%   40%   45%
1.000498 1.099471 1.152448 1.203032 1.228786 Conclusion: 6% of comps have LogS±1 accuracy >90%

median(pred_y_half_width_70perc_AD2)
[1] 0.707375
> quantile(pred_y_half_width_**70perc**_AD2,c(0.9,0.95,0.96,0.97))

90% 95% **96%** 97%
0.9024929 0.9789299 1.0039394 1.0341793 Conclusion: 96% of comps have LogS±1 accuracy > 70%
pred_y_half_width_91perc_AD2<-st_d_y_AD2_2*(sort((abs(telta-B1)/STDEV_CPcv_2),decreasing=TRUE)
[(length(B1)+1) %/% (100/9)]) *fact91
> quantile(pred_y_half_width_91perc_AD2,c(0.01,0.02,0.03,0.035,0.04))
1% 2% 3% 3.5% 4%
0.9390752 0.9735583 0.9943581 1.0029352 1.0112464 Conclusion: 3.5% of comps have LogS±1 accuracy > 91% - but that wouldn't affect significantly our result.

0-6% >90%
6%-75% >80%
75%-96% >70%
69% comps have an average of 85% acc.
21% have an average of 75% acc.
(0.69*0.85+0.21*0.75)/(0.69+0.21)=82.7% - That is the accuracy of the AqSolDB-n

Part 2. Calculation of FSTI-XGB-EM-N model for AqSolDB-w (Aq-w of 3273 compounds):

> median(pred_y_half_width_80perc_AD2) # 80% conf. level
[1] 1.7788
> mean(pred_y_half_width_80perc_AD2) # 80% conf. level
[1] 1.790132
As mean 80% pred. int. of AqSolDB-w equals 1.790132:

> pred_y_half_width_553perc<- error_te_DB*(sort(alfa_value,decreasing=TRUE)[length(B1) %/% (1000/447)])
*fact553
> mean(pred_y_half_width_80perc)/mean(pred_y_half_width_553perc)
[1] 1.78805
1.790132 ≈1.78805
So, 55.3% LogS±1 is estimated accuracy of AqSolDB-w (having 3273 comps out of 72739)

Part 3. Final calculation Aq-n + Aq-w

(68535*0.827+3273*0.553)/(68535+3273)
[1] 0.8145111 (i.e. 81.45%) - total accuracy rate LogS±1 of all PubChem compounds within AD (71808)

Calculation of COCONUT (total 406,919 compounds, 390338 within AD)

Part 1. Calculation of FSTI-XGB-ARSS model for AqSolDB-n (Aq-n of 356,277 compounds):

> median(pred_y_half_width_80perc_AD2)
[1] 0.9180074

> quantile(pred_y_half_width_80perc_AD2,c(0.6,0.65,0.66,0.67,0.7))
60% 65% 66% 67% 70%
0.9638526 0.9897033 0.9950762 1.0005594 1.0176978 Conclusion: 67% of comps have LogS±1 accuracy >80%

> 0.67*356277/406919
[1] 0.586617
58.7% total COCONUT crystal structures are within 1 logS unit with 80% conf. level (Table 8)

> quantile(pred_y_half_width_90perc_AD2,c(0.02,0.045,0.05))
2% 4.5% 5%
0.9508365 0.9990918 1.0063742 Conclusion: 4.5% of comps have LogS±1 accuracy >90%

> quantile(pred_y_half_width_70perc_AD2,c(0.9,0.93,0.936,0.94,0.95))
90% 93% 93.6% 94% 95%
0.9511727 0.9897025 0.9989621 1.0056015 1.0228496 Conclusion: 93.6% of comps have LogS±1 accuracy >70%

quantile(pred_y_half_width_68perc_AD2,c(0.9,0.91,0.92,0.95,0.96,0.965,0.97))
90% 91% 92% 95% 96% 96.5% 97%
0.9046278 0.9154991 0.9276716 0.9727973 0.9919681 1.0027882 1.0147560 Conclusion: 96% of comps have LogS±1 accuracy >68%

acc. of 0-4.5% comps > 90%
So, 4.5% of AqSolDB-n have acc. of LogS±1 >90%
acc. of 4.5%-67% comps > 80% (average between 90% and 80% = 85%)
So, 4.5%-67%, i.e. 62.5% (as 67%-4.5%=62.5%) of AqSolDB-n have estimated acc. of LogS±1 = 85%
acc. of 67%-93.6% comps >70% (average between 80% and 70% = 75%)
So, 67%-93.6%, i.e. 26.6% (as 93.6%-67%=26.6%) of AqSolDB-n have estimated acc. of LogS±1 = 75%
acc. of 93.6%-96% comps >68% (average between 70% and 68% = 69%)
So, 93.6%-96%, i.e. 2.4% (as 96%-93.6%=2.4%) of AqSolDB-n have estimated acc. of LogS±1 = 69%

(0.625*0.85+0.266*0.75+0.024*0.69)/(0.96-0.045) = 0.816733 (81.7%) – That is the accuracy of the AqSolDB-n (having 356277 comps

Part 2. Calculation of FSTI-XGB-EM-N model for AqSolDB-w (Aq-w of 34,061 compounds):

median(pred_y_half_width_80perc)
1.491423
mean(pred_y_half_width_80perc)
1.54571

> mean(pred_y_half_width_80perc/mean(pred_y_half_width_**617perc**)
[1] 1.545277
1.54571 ≈ 1.545277
So, AqSolDB-w accuracy rate is 61.7%

Part 3. Final calculation Aq-n + Aq-w
> 34061/390338
[1] 0.08726027 – fraction of AqSolDB-w compounds
> (390338-34061)/390338
[1] 0.9127397 – fraction of AqSolDB-n compounds

> 0.617*0.08726+0.8167*0.91274

[1] 0.7992742 (79.93%) total accuracy rate rate LogS±1 of 390338 COCONUT compounds within AD (96% of total 406919 compounds)

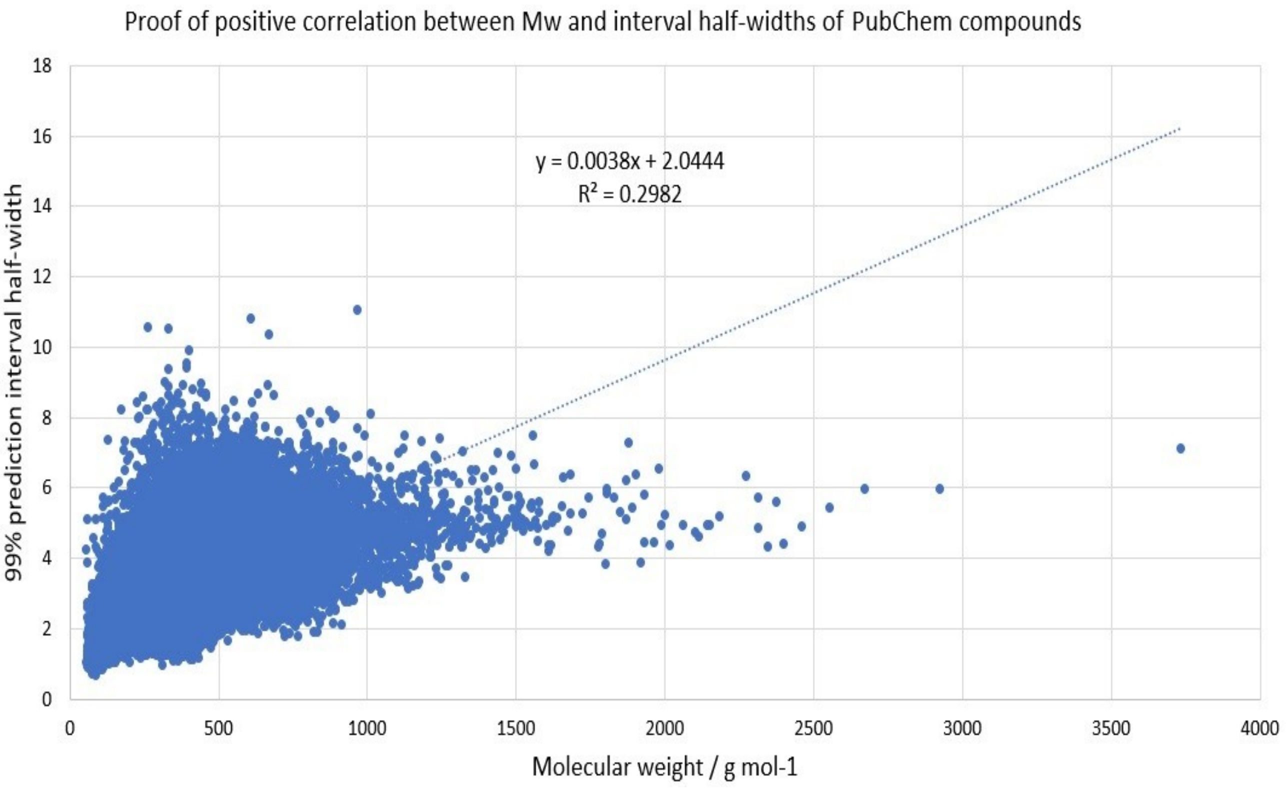

Figure S4. Prediction interval half-widths and Molecular weight of PubChem database. Prediction intervals are obtained using FSTI-XGB-EM-N of the AqSolDB-n data set.

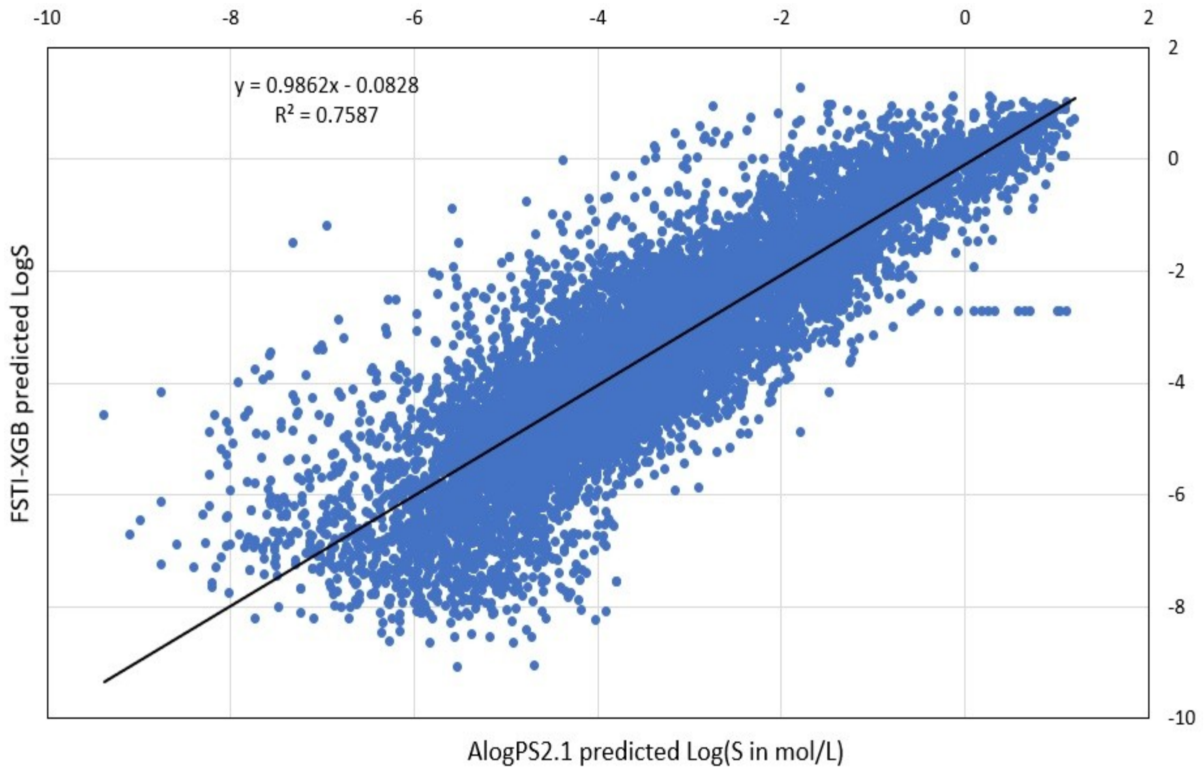

Figure S5. AlogPS2.1 [32] predicted and FSTI-XGB predicted solubilities of the Drugbank database on 10,861 molecular cases. For completeness, RMSE between FSTI-XGB and AlogPS2.1 values = 0.9645 Log(S).

Info section S5: Python code for obtaining Padel and rdkit molecular descriptors from smiles

Obtaining_mol_descriptors:

AFTER SMILES ARE COPY-PASTED FROM LARGEST CONTIGUOUS FRAGMENT OF ANY DATA SET (i.e. sheet) of Supplementary_data_sets_table.xlsx to a new file and then the new file is named as "something".smi. Then "something".smi is put into the same directory where the python3 program is launched below

```
#Padel descriptors:

import numpy as np
import pandas
import pubchempy as pcp
from pubchempy import get_compounds, Compound
import requests
from padelpy import from_smiles
import sys
from padelpy import padeldescriptor

# https://github.com/ecrl/padelpy options for padeldescriptor

padeldescriptor(mol_dir='"something".smi', d_2d=True, maxruntime=30000, detectaromaticity=True, removesalt=True, retain3d=True, retainorder=True, d_file='{file and its location to write Padel descriptors}.csv')

#rdkit descriptors:

import numpy as np

import rdkit

from rdkit import Chem

from rdkit.Chem import Descriptors

import pandas as pd

from rdkit.Chem import AllChem
num_lines=sum(1 for line in open('"something".smi'))

baseData = np.arange(1,1)

j=0

for i in range(num_lines):

   with open('"something".smi') as f:

      line=f.read().split('\n')[i]

      mol = Chem.MolFromSmiles(line)

      desc_MolWt = Descriptors.MolWt(mol)

      desc_MolLogP = Descriptors.MolLogP(mol)
```

```
    desc_MolMR = Descriptors.MolMR(mol)

    desc_HeavyAtomCount = Descriptors.HeavyAtomCount(mol)

    desc_NumHAcceptors = Descriptors.NumHAcceptors(mol)

    desc_NumHDonors = Descriptors.NumHDonors(mol)

    desc_NumHeteroatoms = Descriptors.NumHeteroatoms(mol)

    desc_NumRotatableBonds = Descriptors.NumRotatableBonds(mol)

    desc_NumValenceElectrons = Descriptors.NumValenceElectrons(mol)

    desc_NumAromaticRings = Descriptors.NumAromaticRings(mol)

    desc_NumSaturatedRings = Descriptors.NumSaturatedRings(mol)

    desc_NumAliphaticRings = Descriptors.NumAliphaticRings(mol)

    desc_RingCount = Descriptors.RingCount(mol)

    desc_TPSA = Descriptors.TPSA(mol)

    desc_LabuteASA = Descriptors.LabuteASA(mol)

    desc_BalabanJ = Descriptors.BalabanJ(mol)

    desc_BertzCT = Descriptors.BertzCT(mol)

    row
=[desc_MolWt,desc_MolLogP,desc_MolMR,desc_HeavyAtomCount,desc_NumHAcceptors,desc_NumHDonors,desc_
NumHeteroatoms,desc_NumRotatableBonds,desc_NumValenceElectrons,desc_NumAromaticRings,desc_NumSaturate
dRings,desc_NumAliphaticRings,desc_RingCount,desc_TPSA,desc_LabuteASA,desc_BalabanJ,desc_BertzCT]

    if (j==0):

      baseData=row

    else:

      baseData=np.vstack([baseData,row])

    j=j+1

columnNames=["MolWt","MolLogP","MolMR","HeavyAtomCount","NumHAcceptors",
"NumHDonors","NumHeteroatoms","NumRotatableBonds","NumValenceElectrons","NumAromaticRings","NumSatur
atedRings","NumAliphaticRings","RingCount","TPSA","LabuteASA","BalabanJ","BertzCT"]

descriptors = pd.DataFrame(data=baseData,columns=columnNames)

pd.DataFrame(descriptors).to_csv("{file and its location to write rdkit descriptors}.csv")
```

Info section S6: Calculation of ORCA SMD solvation Gibbs free

energy For two different conformations of a compound:

```
############ Input ORCA file name as "orca solv_En_1.txt"

! B97-D3 def2-TZVP tightopt
%maxcore 4000

* XYZFILE 0 1 md_1.xyz

$new_job
! B97-D3 def2-TZVP tightopt
%maxcore 4000

%cpcm smd true
SMDsolvent "Methanol"
end

* XYZFILE 0 1 solv_En_1.xyz

############ END of ORCA file


############Commands in Terminal:

#!/bin/bash

orca solv_En_1.txt>solv_En_1.out.txt

grep -B 3 "OPTIMIZATION RUN DONE" solv_En_1.out.txt  >   Rezultati.txt
grep 'FINAL' Rezultati.txt > Rez.csv
cat Rez.csv | awk '{print $5}' > Rez_col.csv

R

A<-read.csv('Rez_col.csv',header=FALSE)
t<-t(A)
t<-as.vector(t)
write.csv(paste0("Delta G equals:   ",(t[2]-t[1])*627.5),"Final_result.csv")
```

############ End of commands in Terminal, energy in kcal/mol is given in the final file. Do the same for a different conformation, and take the average and the minimum energy as two QM variables.

Info section S7: Names and meaning of used QMvars for Methanol data set

"MW..g.mol.1." - Molecular weight of the whole API (all fragments)

"ΔHfus..kJ.mol.1." - Fusion enthalpy, values taken from pharmacopoeia

"Tm..K." - Melting point, values taken from pharmacopoeia

"Donor" - number of donor atoms of the whole API (all fragments)

"Acceptor" - number of acceptor atoms of the whole API (all fragments)

"deltaG.minE.in.vac" - Solvation Gibbs free energy in methanol using explicit model in ORCA DFT*, minimum value of one or two calculated largest fragments conformations.

"IntraHbond.count" - number of intramolecular H-bonds

"deltaG.aver" - Solvation Gibbs free energy in methanol using explicit model in ORCA DFT*, average value of one or two calculated largest fragments conformations.

"XlogP3.pubchem" - Values taken from PubChem as "Computed by XLogP3 3.0 (PubChem release 2021.05.07)"

"deltaG.MW" - is division of "deltaG.minE.in.vac" by "MW..g.mol.1..1"

"MW..g.mol.1..1" - Molecular weight of the API's largest contiguous fragment

"Donor.1" - number of donor atoms of the API's largest contiguous fragment

"Acceptor.1" - number of donor atoms of the API's largest contiguous fragment

* See input file for calculation of Solvation Gibbs free energy in methanol using XYZ file of API's largest contiguous fragment conformation: orca_solv_G_input.txt in Info section 6.

Info section S8: Names of QMvars of Water-wide, Ethanol and Acetone data sets

With reference to Ref. 12, we give names for 41 used physicochemical descriptors 'QMvars':

MW = Molecular Weight
MP = Experimental melting point in Deg C
Volume = Molar volume in cm^3 mol^-1
E0_gas = Gas Phase Zero Point Energy in Ha
G_gas = Gas Phase Gibbs Energy in Ha
E0_solv = Solution Phase Zero Point Energy in Ha
G_solv = Solution Phase Gibbs Energy in Ha
DeltaE0_sol = E0_solv - E0_gas
DeltaG_sol = G_solv - G_gas
HF_E0_gas = Gas Phase Zero Point Energy in Ha using HF SMD protocol
HF_G_gas = Gas Phase Gibbs Energy in Ha using HF SMD protocol
HF_E0_solv = Solution Phase Zero Point Energy in Ha using HF SMD protocol
HF_G_solv = Solution Phase Gibbs Energy in Ha using HF SMD protocol
HF_DeltaE0_sol = E0_solv - E0_gas using HF SMD protocol
HF_DeltaG_sol = G_solv - G_gas using HF SMD protocol
gas_dip = Gas phase dipole
solv_dip = Solution phase dipole
HOMO = HOMO energy in eV
LUMO = LUMO energy in eV
LsoluHsolv = LUMO of molecule - HOMO of solvent
LsolvHsolu = LUMO of solvent - HOMO of solute
SASA = Solvent Accessible Surface Area
O_charges = Sum of charges on oxygen atoms
C_charges = Sum of charges on carbon atoms
Most_neg = Charge of most negative atom
Most_pos = Charge of most positive atom
Het_charges = Sum of charges on non-carbon or non-hydrogen atoms
N_atoms = Number of atoms
Area1 = Area of 1st shadow projection
Area2 = Area of 2nd shadow projection (perpendicular to 1st)
Area3 = Area of 3rd shadow projection (perpendicular to 1st and 2nd)
Asp1 = Aspect Ratio of 1st shadow projection
Asp2 = Aspect Ratio of 2nd shadow projection (perpendicular to 1st)
Asp3 = Aspect Ratio of 3rd shadow projection (perpendicular to 1st and 2nd)
No_regions = Number of regions of high/low charges on molecular surface
Tot_charge = Total high/low charge area on molecular surface
Neg_charge = Total low charge area on molecular surface
Pos_charge = Total high charge area on molecular surface
Big_area = Area of biggest region of high/low charge on molecular surface
Big_charge = Average charge of biggest area of high/low charge on molecular surface
Big_std = Standard deviation of charge of biggest area of high/low charge on molecular surface

Info section S9: Calculation of NC measure for any number of calibration samples of certain confidence levels

To obtain an NC measure that equals exactly 99%, 95%, or 90% confidence level, one would need to have 100xl -1 number of samples in the calibration. That would imply that all data sets without such a number of calibration samples cannot be used with CP regressor for estimation of the mentioned confidence levels. We dissent with such implication. If, in our case, we have a data set of 8091 examples (i.e. samples), the closest top-down ordered NC measures would be the 80th and 81st examples. So 80th top-down sorted NC measure would represent (8091-80+1)/(8091+1)% confidence, i.e. means 0,99011% confidence level, while 81st sample would be (8091-81+1)/(8091+1)%, i.e., 0.98999% confidence level. Neither of these two is exactly 99%. But a 99% confidence level would be vicinal to these two points. In that, case, one can select any of these two NC measures by multiplying it by a linear factor *f*. The *f* is defined according to the 99% confidence intercept of the line spanned between the points (α_{81},0.98999%) and (α_{80},0.99011%), and as (x1,y1) and (x2,y2), respectively. The general equations for the case of any confidence level (c), number of calibration samples in the data set (B), and determined NC-s (all α values) for the whole calibration set are below:

```
const = 100*(c+0.01) # for c as confidence level (e.g. 0.99, 95%, etc..)
y2 = ( B - (((B+1) %/% const)-1) ) / (B+1)
y1 = (B - ((B+1) %/% const))/(B+1)
x2 = (sort(α,decreasing=TRUE)[(B+1) %/% const])
x1 = (sort(α,decreasing=TRUE)[((B+1) %/% const)+1])

f=((((1-(1/const))-y1)*(x2-x1)/(y2-y1))+x1)/sort(α,decreasing=TRUE)[(B+1)%/%const]
```

These equations were applied for all studied data sets and all confidence levels in relation to CP, except data sets (1,3) as Methanol and AqSolDB-n had 99 and 1399 training (i.e., calibration) samples for cross-conformal prediction, respectively. So, data sets (1,3) do not need this adjustment.

The rationale for this, is that between two vicinal points, a linearly estimated value of α for set confidence can be approximated for the case of enough high number of calibration samples in the data set. This is because any non-linear curve can be linearly presented at its narrow range of the curve (e.g., between 0.9898% and 0.9902%).
